# Core-size and geometry *versus* toxicity in small amino terminated PAMAM dendrimers†

**DOI:** 10.1039/d4ra02020k

**Published:** 2024-09-10

**Authors:** Claus Bøge Hansen, Anna Janaszewska, Monika Dąbrzalska, Monika Marcinkowska, Barbara Klajnert-Maculewicz, Jørn Bolstad Christensen

**Affiliations:** a Department of Chemistry, Faculty of Science, University of Copenhagen Thorvaldsensvej 40 DK-1871 Frederiksberg Denmark jbc@chem.ku.dk; b Department of General Biophysics, Faculty of Biology and Environmental Protection, University of Lodz 141/143 Pomorska Street 90-236 Lodz Poland

## Abstract

A series of 6 small PAMAM dendrimers (G0-dendrimers) differing in the size, polarity and flexibility has been synthesized. The toxicity has been investigated in three different human cancer cell lines (HeLa, MCF-7, THP-1) and the endothelial skin cell line HMEC-1 in order to evaluate their potential as vehicles for drug delivery.

## Abstract 2

The toxicity of 6 small dendrimers has been investigated in three different human cancer cell lines (HeLa, MCF-7, THP-1) and the endothelial skin cell line HMEC-1 in order to evaluate their potential as vehicles for drug delivery.

## Graphical abstract

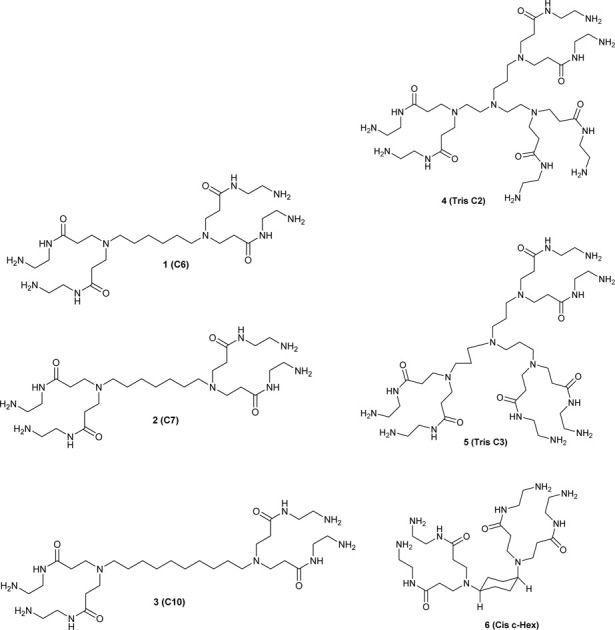

## Introduction

Dendrimers, which are well-defined synthetic macromolecules with a tree-like branched structure, are beginning to find their place in the big family of nanoparticles that one day may be the next generation of drugs in nanomedicine.^1^ PAMAM dendrimers are the workhorse for most applications of dendrimers in nanomedicine due to their commercial availability and synthesis that is deceptively simple. However, the majority of PAMAM dendrimers studied have either a 1,2-ethanediamine (EDA) or 1,4-butanediamine (DAB) as cores and there are only a few studies on the properties of PAMAM dendrimers possessing different cores. This is interesting because the first PAMAM dendrimers reported had an ammonia-core.^2^ The concept of trivalent cores has since been studied by Jayaraman^3^ (polyether amines) and Peng and coworkers as a way of getting flexible dendrimers that are excellent carriers for siRNA.^4–8^ The interplay between flexibility and binding affinities for siRNA and DNA was studied by the groups of Danani^9–11^ and Pricl^6,7,10,12^ and has been shown to be a very important parameter to consider, when designing dendrons and dendrimers for exo-complexation of larger molecules.

The guest–host chemistry of a series of PAMAM dendrimers with cores from C2 up to C12 was studied by the groups of Turro and Tomalia^13^ using Nile red as a hydrophobic reporter dye. They reported the formation of supramolecular complexes between hydrophobic core dendrimers and the detergent SDS. Hydrophobicity can be used as a parameter to increase transfection efficiency by having fatty acid amides or lipids on the surface of PAMAM dendrimers^14–17^ or by using PAMAM dendrimers having a lipophilic core as reported by Cheng and coworkers.^18^ They reported that the cytotoxicity decreased as the length of the core increased in a series of G4 PAMAMs with 1,2-ethanediamine (C2), 1,6-hexanediamine (C6) or 1,12-dodecanediamine (C12) cores. Binding of G4 PAMAM dendrimers (64 surface groups) with different linear hydrophobic cores to albumin as a model protein was studied by Diallo *et al.*^19^ They found that increasing the length of the hydrophobic core led to decreased binding to albumin, which is interesting since the immunotoxicity of amino terminated DAB-core PAMAM dendrimers is due to binding to IgM in the bloodstream.^20^

In the present study, we investigated the influence of different cores on the toxicity of small PAMAM dendrimers, which could be used as carriers of DNA, siRNA or as multivalent drugs displaying drug molecules at the surface. For a recent example on a multivalent antibiotic see Christensen and coworkers.^21^ Small dendrimers are expected to have a wider biodistribution due to their smaller size while keeping the multivalency and they should be more easily excreted than larger dendrimers. Additionally, their synthesis involves fewer steps, which means faster synthesis and less problems with defective structures.

## Results and discussion

**Fig. 1:**
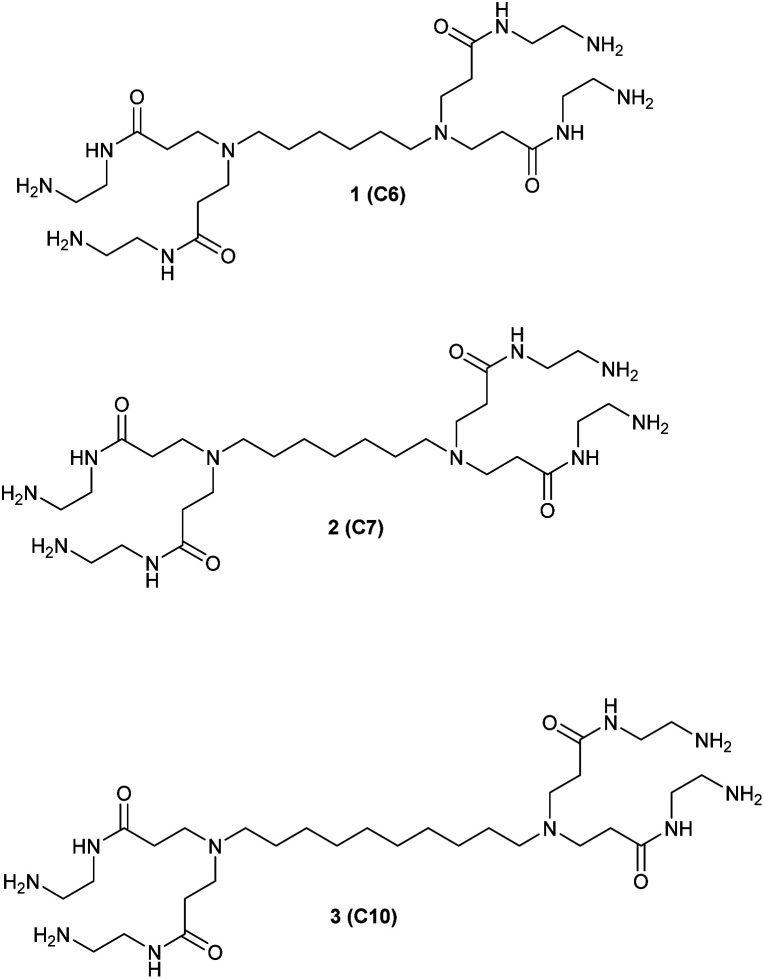
The three straight chain G0 PAMAM dendrimers synthesized and investigated.

**Fig. 2:**
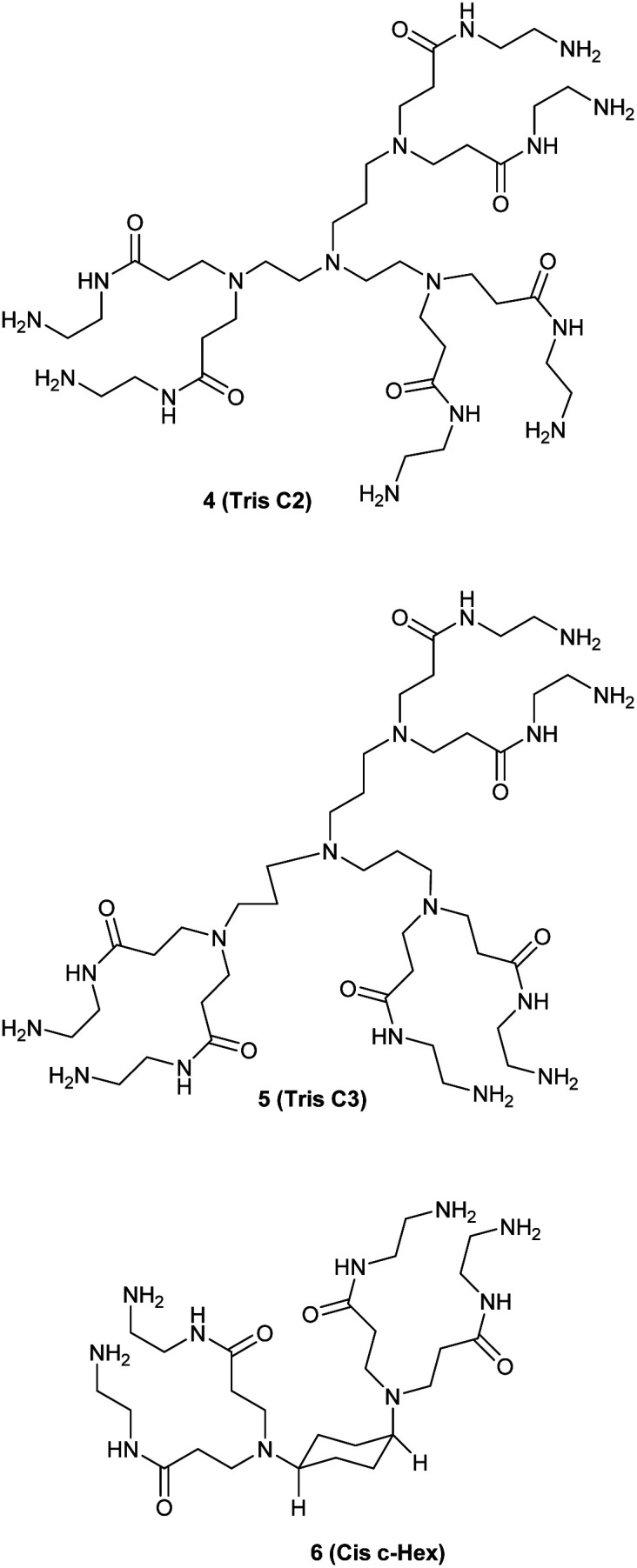
The two triamine-based G0 dendrimers and the *cis*-1,4-cyclohexanediamine cored G0.

We studied the G0-PAMAM dendrimers shown in Fig. 1 and 2.

**Scheme 1:**
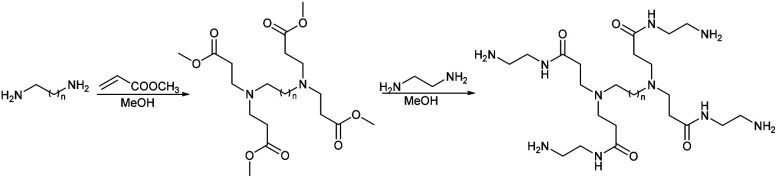
Divergent synthesis of dendrimers.

**Fig. 3:**
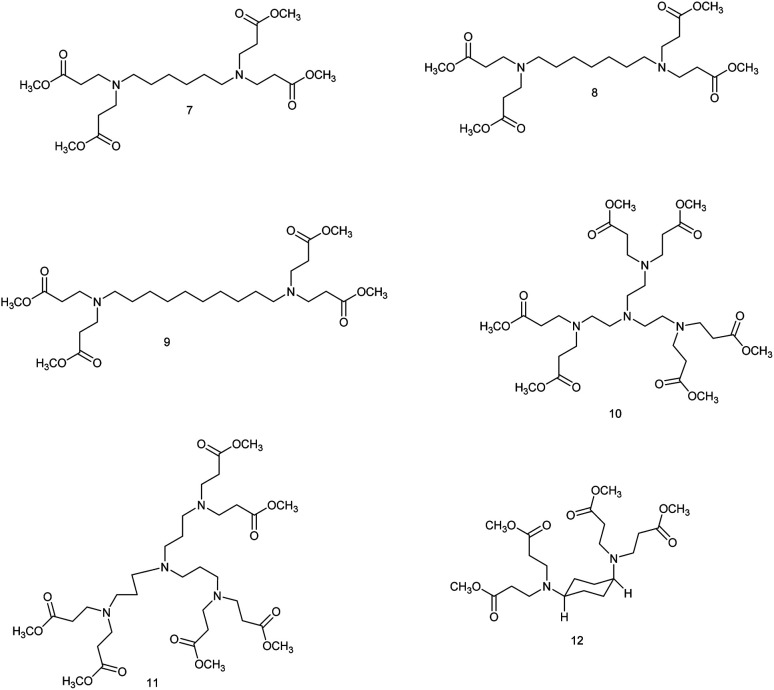
The intermediate G 0.5 dendrimers.

The dendrimers 1–3 (Fig. 1) all have a tetravalent core but differ in the chain length of the core (C6, C7 and C10). The dendrimers 4 & 5 (Fig. 2) have trivalent cores based on tris(2-aminoethyl)- and tris(3-aminopropyl)amine respectively, which should give more flexible dendrimers^3,4^ and dendrimer 6 has *cis*-1,4-diaminocyclohexane as the core, which reduces the flexibility of the dendrimer considerably because the two focal-point are locked in a *cis*-conformation (Fig. 2). Dendrimer 6 is therefore expected to be a mimic of larger size dendrimers with a 1,4-diaminobutane core (DAB-core). The dendrimers were synthesized by the divergent methodology^1^ (Scheme 1) starting from the commercially available diamines (Fig. 3).

**Fig. 4:**
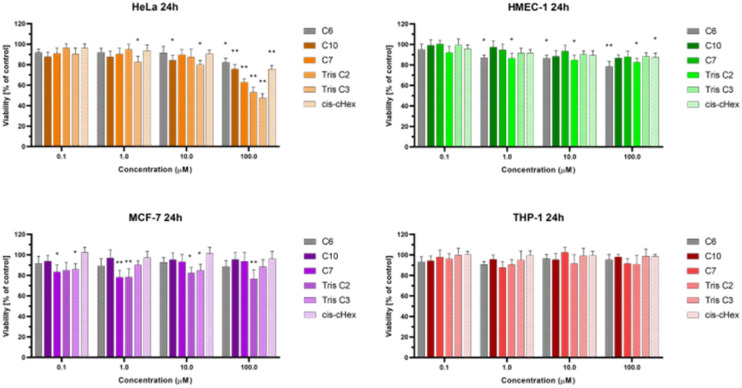
The influence of small PAMAM dendrimers on viability of HeLa, MCF-7, THP-1 cells and non-cancerous HMEC-1 cells after 24 h incubation at 37 °C. Data are presented as a percentage of control (untreated cells) ±standard deviation (SD). *p < 0.05, **p < 0.001 – relative to the control.

**Fig. 5:**
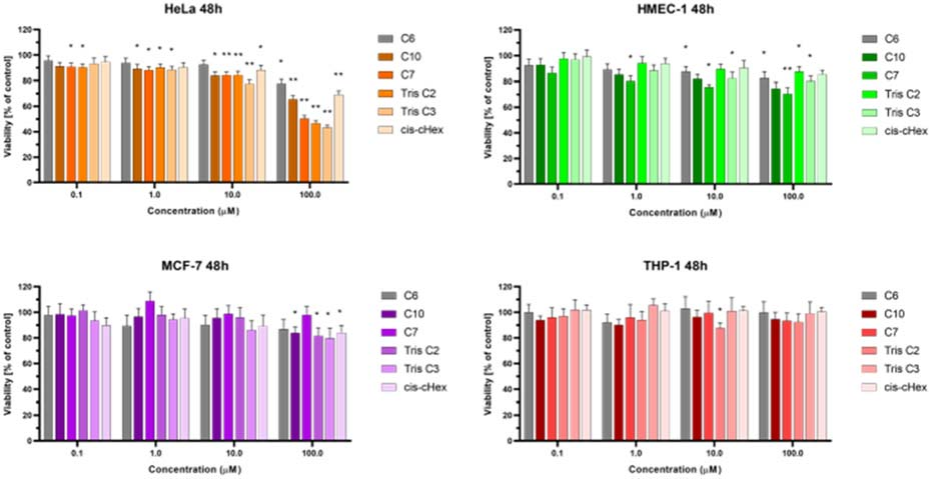
The influence of small PAMAM dendrimers on viability of HeLa, MCF-7, THP-1 cells and non-cancerous HMEC-1 cells after 48 h incubation at 37 °C. Data are presented as a percentage of control (untreated cells) ±standard deviation (SD). *p < 0.05, **p < 0.001 – relative to the control.

We chose three cancer cell lines for the biological studies: HeLa (human epitheloid cervix carcinoma), MCF-7 (human breast adenocarcinoma), THP-1 (human acute monocytic leukemia) and non-cancer human dermal microvascular endothelium HMEC-1 cells to analyze the cytotoxicity of group of small PAMAM dendrimers. In the investigation of potential carriers of anti-cancer drugs or genetic material it is important to test both cancerous and non-cancerous cell lines. The first step was to evaluate the cytotoxicity of six newly synthesized compounds. After 24 and 48 hours of incubation, only C7, Tris C2 and Tris C3 were found to be toxic at the highest used 100 μM concentration, reducing the viability of HeLa cells to 62%, 53%, 47% respectively after 24 hours of incubation (Fig. 4) and to 50%, 46%, 43% after 48 hours of incubation (Fig. 5).

After analyzing the obtained results, a surprising selective toxicity of the mentioned C7, Tris C2 and Tris C3 towards cervix carcinoma (HeLa) cells can be observed. This effect is visible after 24 hours of incubation and deepens by an average of 10% after another 24 hours of incubation with the compounds. At the same time, for the same compounds, the cell viability of the cancer cell lines does not fall below 80%. Interestingly, the cell viability of non-cancerous HMEC-1 cells oscillates around this value even after 48 h of incubation. As the differences between viability of tested cell lines were most significant for the compounds used in the concentration of 100 μM, we chose this concentration for further studies.

The observed effect may depend on a different metabolism, set of cellular transporters and the sensitivity of the cell lines selected for the study. Two of them were derived from solid tumors (HeLa and MCF-7), where MCF-7 is a hormone-dependent cell line. THP-1 is a monocyte cell line isolated from the peripheral blood of a patient with acute monocytic leukemia, while the fourth HMEC-1 cell line is non-cancerous, derived from dermal microvascular endothelium. Previous research has shown a correlation between the generation of dendrimers, their toxicity, and their efficiency in penetrating cells. The higher the generation, the greater the toxicity of the dendrimer, but also the higher the positive charge, facilitating easier interactions with the negatively charged cell membrane.^22^ However, there are also studies showing that dendrimers of lower generations can also be successfully used as carriers for drugs or genetic material.^23^ Therefore, our initial results for the tested dendrimer cores, obtained using the MTT method, confirm the selective toxicity of some of them. This paves the way for their potential future use as drug or genetic material carriers.

**Fig. 6:**
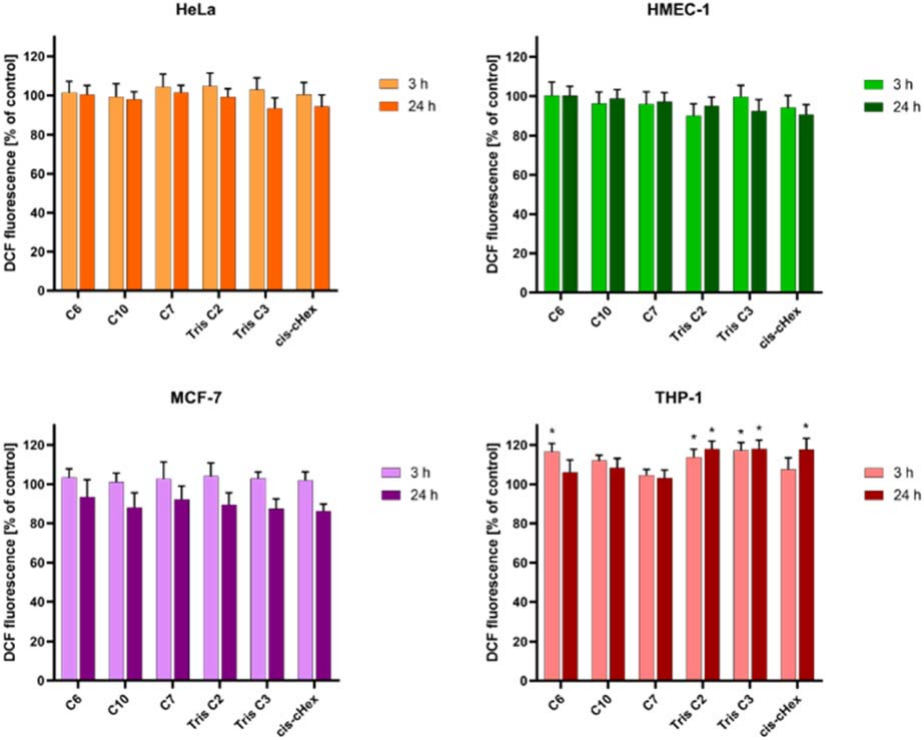
The influence of small PAMAM dendrimers at a concentration of 100 μM on the ROS generation in HeLa, MCF-7, THP-1 cells and non-cancerous HMEC-1 cells after 3 and 24 h incubation at 37 °C. Data are presented as the mean ± standard deviation (SD) of three experiments. *p < 0.05, – relative to the control.

Bearing in mind that toxicity can be also correlated with increased production of ROS and ultimately induction of cell death, and that the interaction of even slightly toxic compounds with mitochondria leads to ROS production, we decided to determine the level of reactive oxygen species and mitochondrial membrane potential for analysed compounds after 3 and 24 hours. The ROS production was assessed by measurement of the highly fluorescent dichlorofluorescein (DCF), a compound formed after the oxidation of non-fluorescent H_2_DCF-DA probe by cytosolic esterases.^24^ Only for the THP-1 suspension cell line, where cells are in contact with the compounds with their entire surface, a minor but statistically significant 16–17% increase in the amount of generated ROS was observed (Fig. 6). Contrary, we did not observe increased ROS production in HeLa cells. The lack of correlation between cytotoxicity tests and ROS production in adherent HeLa cells may be due to the postulated lack of sensitivity of H_2_DCFDA probe directly to singlet oxygen *versus* high sensitivity to peroxy products and peroxy radicals.^25^

**Fig. 7:**
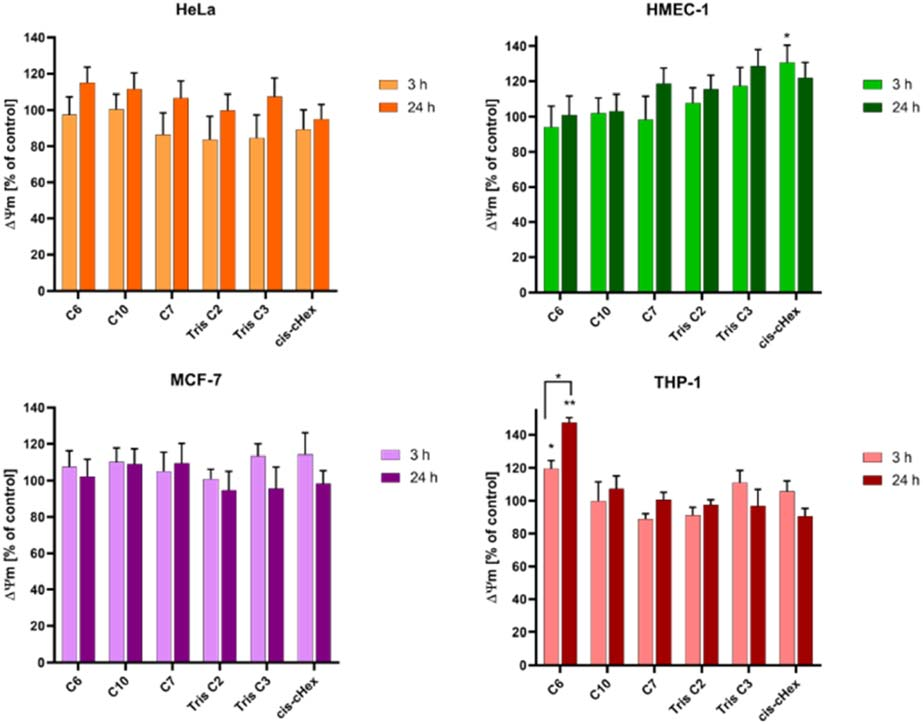
The influence of small PAMAM dendrimers at a concentration of 100 μM on the mitochondrial membrane potential in HeLa, MCF-7, THP-1 cells and HMEC-1 cells after 3 and 24 h incubation at 37 °C. Data are presented as the mean ± standard deviation (SD) of three experiments. *p < 0.05, **p < 0.001 – relative to the control.

Since not only the production of free radicals, but also broadly understood oxidative stress affects the mitochondria, and cell redox homeostasis is a key factor in the modulation of apoptosis,^26,27^ it is very important to check whether the tested compounds can initiate oxidative processes resulting from the production of ROS in cells, such as changes in the mitochondrial potential. Activation of mitochondria as a result of oxidative stress most often leads to changes in the potential of the mitochondrial membrane, changes in its permeability and the formation of apoptotic proteins in the cell. Therefore, it is believed that a change in the mitochondrial membrane potential can lead to cell death by opening channels in the mitochondrial membrane.^28^ Mitochondrial depolarization, *i.e.* a decrease in the potential of the mitochondrial membrane, accompanies the early stages of apoptosis and is usually preceded by hyperpolarization of the mitochondrial membrane by blocking the voltage-dependent anion channel (VDAC) with the Bax peptide^29^ or generating a proton gradient across the inner membrane.^30^ The cationic carbocyanine dye JC1 was used to assess changes in mitochondrial potential. The dye as a monomer emits green fluorescence and, forming aggregates, it shows a wide excitation spectrum and an emission maximum at ∼590 nm. Therefore, the fluorescence ratio of monomers and aggregates is a sensitive marker of mitochondrial membrane potential. After 24 hours of incubation, all analysed compounds showed slight hyperpolarization (Fig. 7) and only values obtained for *cis*-cHex in HMEC-1 cells and C6 in THP-1 cells were statistically significant compared to untreated control cells. This result differs from the results obtained for larger dendrimers. As Mukherjee *et al.*^31^ studies have shown, the amine-terminated dendrimer can reduce the mitochondrial membrane potential in a concentration-dependent manner-the higher the toxicity of the dendrimer, the greater was the depolarization of the mitochondrial membrane.

When analysing the changes in the mitochondrial membrane potential, we did not notice any significant differences in the effect of the tested compounds depending on the cell line. This is because the compounds tested were non-toxic against THP-1, MCF-7 and HMEC-1 cells (in the tested concentration range, cell viability decreased to 80% at most). In the case of HeLa cells, there is most likely different mechanism of cell death involved. Therefore, the next step was to analyse the fraction of apoptotic and necrotic cells.

**Fig. 8:**
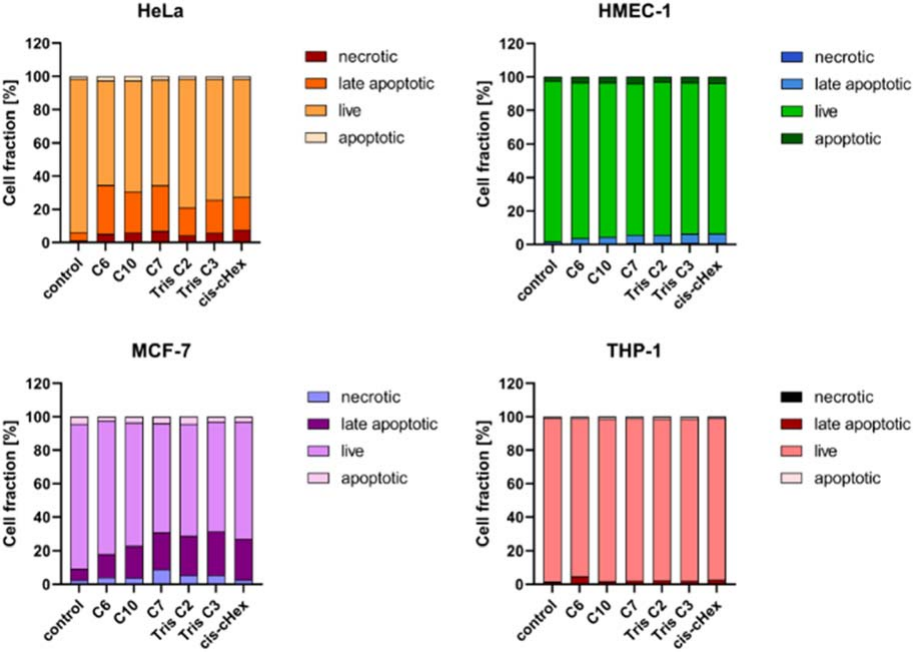
The influence of small PAMAM dendrimers at a concentration of 100 μM on the fractions of apoptotic and necrotic cells in HeLa, MCF-7, THP-1 cells and non-cancerous HMEC-1 cells after 24 h incubation at 37 °C. Data are presented as the mean ± standard deviation (SD) of three experiments.

Apoptosis is defined as programmed cell death, characterized by nuclear chromatin condensation, cell shrinkage, DNA fragmentation, exposure of apoptotic bodies, and phosphatidylserine symmetry changes. In contrast to apoptosis, necrosis is more rapid and is characterized by loss of membrane integrity, cessation of metabolism, and release of cytoplasmic components.^27,32^ Annexin V conjugated fluorescein isothiocyanate (FITC) and propidium iodide (PI) staining was used to estimate the proportion of apoptotic and necrotic cells. Phosphatidylserine located close to the surface of the cell membrane of apoptotic cells can bind to annexin V, while propidium iodide (PI) penetrates into the interior of necrotic cells. Unfortunately, phosphatidylserine in necrotic cells can also be partially bound with annexin V, which is why they have been labelled as a pool of late apoptotic cells. Fig. 8 shows the increased proportion of late apoptotic and necrotic cells in the HeLa and MCF-7 cell lines, which may confirm a different mechanism of action of the tested compounds in these cells.

**Fig. 9:**
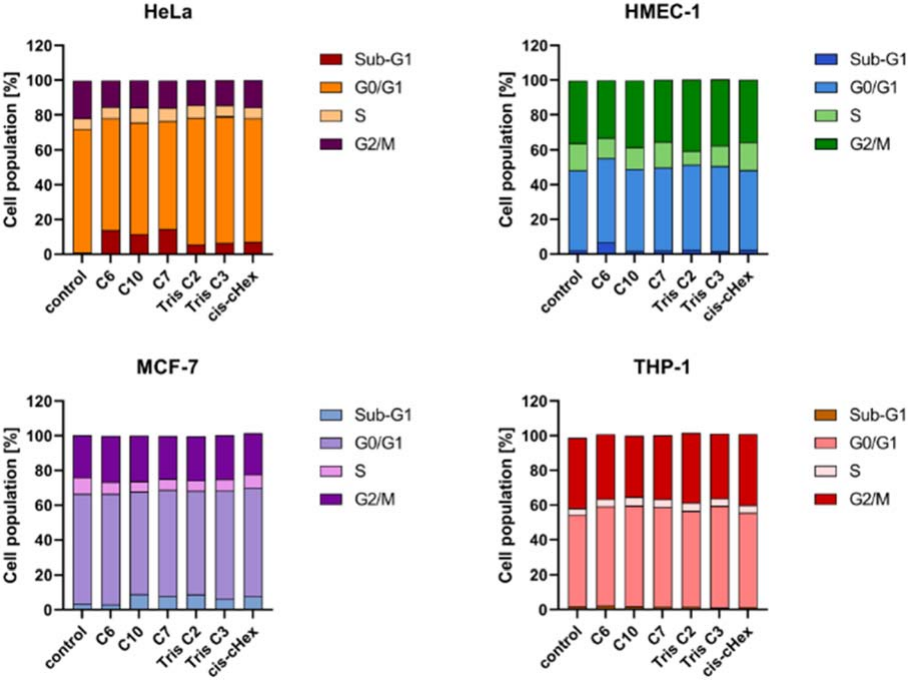
Cell cycle analysis for control (untreated cells) and cells treated with 100 μM of the small PAMAM dendrimers in HeLa, MCF-7, THP-1 cells and HMEC-1 cells after 24 h incubation at 37 °C. Data are presented as the mean ± standard deviation (SD) of three experiments.

Since the analysed compounds have an effect on the induction of late apoptosis or necrosis in two cell lines of adherent cancer cells, but not in suspension cancer cells and non-cancerous cells, we decided to test their effects on the cell cycle, checkpoint arrest and influence on the mitotic process. The cell cycle consists of several phases: G1 – preparation for DNA synthesis, S – phase of DNA synthesis, G2 – preparation for mitosis, M – mitosis in which the cell divides into two similar cells. There are also checkpoints in these phases that ensure genome integrity. The G1 and G2 checkpoints stop the cell cycle when DNA damage is detected at the S phase checkpoint, the cycle stops due to a DNA replication problem and the M phase checkpoint is responsible for stopping the cell when there is a problem with mitotic spindle assembly.^33^Fig. 9 summarizes the cell cycle analysis results for control (untreated) cells and cells treated with analysed compounds.

The tested compounds had no statistically significant effect on THP-1 suspension cells (slight 2–4% cycle arrest in G0/G1 phase) and non-cancerous adherent HMEC-1 cells (4% Tris C2 cycle arrest in G2/M phase and 4% cycle arrest C6 in subphase G1). The results in the form of density plots and histograms of the cell cycle, which are the basis for the analysis of the cycle phase distribution shown in Fig. 9, are available in the ESI material.† However, in the case of adherent cancer cells, they blocked the progression of the HeLa cell cycle in the G2/M phase and the MCF-7 cell cycle in the Sub-G1 phase, inducing apoptosis, observed as 11–14% and 5% of the Sub-G1 population, respectively.

To summarize the obtained results, after 24 h of incubation, C7, Tris C2, and Tris C3 dendrimers at the highest concentration of 100 μM were found to be most toxic only to the HeLa line. However, after 48 h of incubation, a decrease in viability was observed not only in HeLa cells but also in MCF-7 cells. The formation of free radicals, particularly peroxide products and peroxide radicals, was observed to a lesser extent only in the THP-1 line. Statistically significant mitochondrial membrane potential values obtained for *cis*-CHex in HMEC-1 cells and C6 in THP-1 cells indicated that another mechanism is responsible for the cytotoxic effect of the tested compounds on the HeLa line. Analysis of the apoptotic and necrotic cell fractions revealed an increased proportion of late apoptotic and necrotic cells in the HeLa and MCF-7 cell lines, suggesting a different mechanism of action of the tested compounds in these cells compared to THP-1 or HMEC-1 cells. Moreover, analysis of the distribution of cell cycle phases demonstrated differences in the effect of the tested compounds: they blocked the HeLa cell cycle in the G2/M phase and MCF-7 cells in the Sub-G1 phase. These results indicate different mechanisms of action depending on whether the line is a suspension line (THP-1), an adherent cancer cell line (HeLa, MCF-7), or a non-cancer cell line (HMEC1). They also suggest that the toxicity of compounds depends on the rate of cell division (MCF-7 *vs.* HeLa). The longer the cell division time, the later the effects of the compounds are observed. These findings highlight aspects that need to be considered in further in-depth research to elucidate the exact mechanism of the selective toxic effect of the tested compounds on cervical cancer cells.

## Conclusion

A series of small amino terminated (G0) PAMAM dendrimers with different cores has been synthesized, and some of their biological properties have been investigated *in vitro*. The cytotoxicity results obtained were not consistent with predictions based on literature data. Generally, G0 dendrimers are characterized by low cytotoxicity. However, in the case of the tested dendrimers, there is selective toxicity towards cervical cancer cells, which is very surprising. Further research opens the possibility of their potential use as selective carriers of anticancer drugs or genetic material for silencing gene expression in cancer cells.

## Experimental

### General procedure for the full generation dendrimers

#### 3,3′,3′′,3′′′-(Hexane-1,6-diylbis(azanetriyl))tetrakis(*N*-(2-aminoethyl)propanamide) (1)

7.08 g compound 7 (15.4 mmol) dissolved in 45 mL methanol was infused to a solution of 32 mL ethane-1,2-diamine (479.2 mmol) and 20 mL methanol under nitrogen sphere and stirred for a week. The solution was evaporated at 30 °C and 200 mbar, and dried with a nitrogen flow overnight. The resulting oil purified by 3× precipitation from methanol with diethyl ether: dissolved in MeOH (40 mL) and slow addition to diethyl ether (1 L) with stirring followed be decantation of the solvent from the oil. The oil was dried in a nitrogen stream for 3 days. Yield: 7.64 g (87%). Colorless oil. ^1^H-NMR (500 MHz, CD_3_OD): *δ* 1.50–1.55 (br s, 4H), 1.64–1.73 (br s, 4H), 2.55–2.60 (t, 8H, 6.9 Hz), 2.64–2.69 (t, 4H, 7.5 Hz) 2.92–2.99 (m, 16H), 3.44–3.48 (t, 8H, 6.4 Hz). ^13^C-NMR (126 MHz, CD_3_OD): *δ* 27.98, 28.30, 34.53, 42.09, 42.94, 50.88, 54.53, 175.05. ESP-MS: *m*/*z* = 573.46 [M + H^+^]. Calcd 572.80.

#### 3,3′,3′′,3′′′-(Heptane-1,7-diylbis(azanetriyl))tetrakis(*N*-(2-aminoethyl)propanamide) (2)

Yield: 10.2 g (89%) of a transparent oil.

^1^H-NMR (500 MHz, CD_3_OD): *δ* 1.40–1.52 (br s, 6H), 1.59–1.67 (br s, 6H), 2.47–2.55 (t, 8H, 6.9 Hz), 2.58–2.63 (t, 4H, 7.5 Hz), 2.85–2.94 (m, 16H), 3.38–3.42 (t, 8H, 6.4 Hz).

^13^C-NMR (126 MHz, CD_3_OD): *δ* 27.93, 28.61, 30.63, 34.50, 42.09, 42.99, 50.88, 54.54, 175.15.

ESP-MS: *m*/*z* = 587.47 [M + H^+^]. Calcd 586.83.

#### 3,3′,3′′,3′′′-(Decane-1,10-diylbis(azanetriyl))tetrakis(*N*-(2-aminoethyl)propanamide) (3)

6.00 g compound 9 (11.6 mmol) dissolved in 24 mL methanol was infused to a solution of 24 mL ethane-1,2-diamine (359.4 mmol) and 17.5 mL methanol under nitrogen atmosphere and stirred for a week. The solvent was evaporated at 40 °C and 189 mbar followed by oil pump. The resulting oil was thrice dissolved in 40 mL methanol and precipitated in 1 L of diethyl ether. The precipitate was dissolved in 100 mL methanol and evaporated at 40 °C and 200 mbar. The oil was dried in vacuum (oil pump) for 2 days.

Yield: 6.06 g (83%), yellow oil.

^1^H-NMR (500 MHz, CD_3_OD): *δ* 1.32–1.44 (br s, 12H), 1.51–1.58 (m, 4H), 2.41–2.48 (t, 8H, 6.9 Hz), 2.50–2.55 (t, 4H, 7.6 Hz), 2.77–2.88 (m, 16H), 3.30–3.35 (t, 8H, 6.4 Hz).

^13^C-NMR (126 MHz, CD_3_OD): *δ* 27.98, 28.68, 30.73, 30.81, 34.47, 42.05, 42.99, 50.87, 54.62, 175.26.

ESP-MS: *m*/*z* = 629.52 [MH]^+^. Calcd 628.91.

#### 3,3′,3′′,3′′′-((((3-(Bis(3-((2-aminoethyl)amino)-3-oxopropyl)amino)propyl)azanediyl)bis(ethane-2,1-diyl))bis(azanetriyl))tetrakis(*N*-(2-aminoethyl)propanamide) (4)

Yield: 4.22 g (83%) as an yellow oil.

^1^H-NMR (500 MHz, CD_3_OD): *δ* 2.42–2.50 (t, 12H, 6.9 Hz), 2.63–2.69 (br s, 12H), 2.79–2.84 (t, 12H, 6.4 Hz), 2.84–2.90 (t, 12H, 6.9 Hz), 3.30–3.36 (t, 12H, 6.4 Hz).

^13^C-NMR (126 MHz, CD_3_OD): *δ* 34.72, 42.06, 43.00, 51.29, 52.23, 53.71, 175.12, 175.55.

ESP-MS: *m*/*z* = 831.64 [MH^+^], 416.32 [M + 2H^+^]. Calcd 831.13.

#### 3,3′,3′′,3′′′,3′′′′,3′′′′′-((Nitrilotris(propane-3,1-diyl))tris(azanetriyl))hexakis(*N*-(2-aminoethyl)propanamide) (5)

Yield: 4.36 (86%), yellow solid.

^1^H-NMR (500 MHz, CD_3_OD): *δ* 1.64–1.75 (br s, 6H), 2.40–2.47 (t, 12H, 7.0 Hz), 2.47–2.60 (m, 12H), 2.76–2.82 (t, 12H, 6.4 Hz), 2.82–2.87 (t, 12H, 6.9 Hz), 3.30–3.35 (t, 11H, 6.4 Hz).

^13^C-NMR (126 MHz, CD_3_OD): *δ* 24.94, 34.53, 42.06, 42.98, 50.84, 52.68, 53.11, 175.17, 175.35.

ESP-MS: *m*/*z* = 873.69[MH^+^], 437.35 [M + 2H^+^]. Calcd 873.21.

#### 3,3′,3′′,3′′′-(((1*S*,4*S*)-Cyclohexane-1,4-diyl)bis(azanetriyl))tetrakis(*N*-(2-aminoethyl)propanamide) (6)

Yield: 11.6 g (90%) as a yellow oil.

^1^H-NMR (500 MHz, CD_3_OD): *δ* 1.43–1.51 (m, 4H), 1.85–1.96 (m, 4H), 2.37–2.44 (t, 8H, 7.1 Hz), 2.61–2.68 (br s, 2H), 2.75–2.81 (t, 8H, 6.4 Hz), 2.87–2.93 (t, 8H, 7.1 Hz), 3.28–3.33 (t, 8H, 6.4 Hz).

^13^C-NMR (126 MHz, CD_3_OD): *δ* 26.66, 34.89, 42.05, 43.02, 47.11, 57.61, 175.53.

ESP-MS: *m*/*z* = 571.44 [MH^+^]. Calcd 570.78.

### General procedure for the half-generation dendrimers

#### Tetramethyl 3,3′,3′′,3′′′-(hexane-1,6-diylbis(azanetriyl))tetrapropionate (7)

1,6-Diaminohexane (5.52 g; 47.5 mmol) was dissolved in 125 mL methanol and dropwise added to a solution of 25 mL freshly distilled methyl acrylate (275.9 mmol) and 10 mL methanol at 40 °C under a nitrogen atmosphere and stirred for a week at 40 °C. The solution was evaporated at 40 °C and 200 mbar. The resulting oil was purified by column chromatography on Silica gel 60 Å (0.040–0.063 mm) with ethyl acetate as eluent.

Yield: 15.30 g (70%) transparent oil.

^1^H-NMR (500 MHz, CD_3_OD): *δ* 1.28–1.35 (br s, 4H), 1.42–1.51 (br s, 4H), 2.42–2.51 (m, 12H), 2.74–2.81 (t, 8H 7.1 Hz), 3.66–3.70 (s, 12H).

^13^C (126 MHz, CD_3_OD): *δ* 28.03, 28.23, 33.08, 50.27, 51.98, 54.67, 174.39.

ESP-MS: *m*/*z* = 231.15 [M + 2H^+^], 461.29 [MH^+^], calcd: 460.57.

#### Tetramethyl 3,3′,3′′,3′′′-(heptane-1,7-diylbis(azanetriyl))tetrapropionate (8)

Yield: 90%, transparent oil.

^1^H-NMR (500 MHz, CD_3_OD): *δ* 1.31–1.41 (m, 6H), 1.46–1.55 (m, 4H), 2.44–2.54 (m, 12H), 2.79–2.83 (t, 8H, 7.0 Hz), 3.70–3.75 (s, 10H).

^13^C-NMR (126 MHz, CD_3_OD): *δ* 28.01, 28.32, 28.41, 30.23, 30.52, 33.12, 50.32, 52.08 54.81, 174.67.

ESP-MS: *m*/*z* = 497.28 [M + Na^+^], 475.30 [MH^+^]. Calcd 474.60.

#### Tetramethyl 3,3′,3′′,3′′′-(decane-1,10-diylbis(azanetriyl))tetrapropionate (9)

5.55 g (32.2 mmol) Decane-1,10-diamine suspended in 125 mL methanol was dropwise added to a solution of 16 mL methyl acrylate (176.6 mmol) and 8 mL methanol at 40 °C under nitrogen sphere and stirred for a week. The solution was evaporated at 40 °C and 200 mbar. The resulting oil was run through a column of silica gel 60 Å with ethyl acetate as eluent. The fractions was combined and evaporated at 40 °C and 220 mbar. To remove remaining ethyl acetate, the resulting oil was twice dissolved in 100 mL of methanol and evaporated at 40 °C and 200 mbar. The oil was dried in vacuum (oil pump) for 2 days. Yield: 11.8 g (71%), yellow oil. ^1^H-NMR (500 MHz, CD_3_OD): *δ* 1.33 (br s, 12H), 1.49 (m, 4H), 2.38 (t, 8H, *J* = 7 Hz), 2.47 (t, 4H, 7.0 Hz), 2.78–2.79 (m, 20H), 3.26 (t, 8H, *J* = 7 Hz), 3.37 (s, 12H). ^13^C-NMR (126 MHz): *δ* 28.06, 28.43, 30.63, 30.71, 33.12, 50.33, 52.05, 54.82, 174.68. ESP-MS: *m*/*z* = 517.35 [MH]^+^, 539.33 [M + Na]^+^. Calcd 516.68.

#### Tetramethyl 3,3′,3′′,3′′′-((((3-(bis(3-methoxy-3-oxopropyl)amino)propyl)azanediyl)bis(ethane-2,1-diyl))bis(azanetriyl))tetrapropionate (10)

Yield: 27% as an yellow oil.

^1^H-NMR (500 MHz, CD_3_OD): *δ* 2.56–2.62 (t, 12H, 7.0 Hz), 2.65–2.70 (s, 12H), 2.86–2,93 (t, 12H, 7.0 Hz), 3.75–3.80 (s, 18H). ^13^C-NMR (126 MHz, CD_3_OD): *δ* 33.29, 50.85, 52.1, 52.06, 52.56, 54.05, 174.51.

ESP-MS: *m*/*z* = 663.39 [MH^+^]. Calcd 662.78.

#### Hexamethyl 3,3′,3′′,3′′′,3′′′′,3′′′′′-((nitrilotris(propane-3,1-diyl))tris(azanetriyl))hexapropionate (11)

Yield: 64% as an yellow oil.

^1^H-NMR (500 MHz, CD_3_OD): *δ* 1.63–1.71 (t, 6H, 6.7 Hz), 2.50–2.55 (t, 24H, 6.9 Hz), 2.80–2.85 (t, 12H, 7.0 Hz), 3.69–3.76 (s, 18H).

^13^C-NMR (126 MHz, CD_3_OD): *δ* 25.02, 33.17, 50.33, 52.06, 52.98, 53.07, 174.59.

ESP-MS: *m*/*z* = 705.44 [MH^+^], 235.15 [M+ 3H^+^]. Calcd 704.86.

#### Tetramethyl 3,3′,3′′,3′′′-(((1*S*,4*S*)-cyclohexane-1,4-diyl)bis(azanetriyl))tetrapropionate (12)

Yield: 60% as a yellow oil from *cis*-1,4-cyclohexanediamine.

^1^H-NMR (500 MHz, CD_3_OD): *δ* 1.39–1.47 (m, 4H), 1.80–1.89 (m, 4H), 2.47–2.52 (t, 8H, 7.1 Hz), 2.56–2.61 (m, 2H), 2.87–2.93 (t, 8H, 7.1 Hz), 3.69–3.73 (s, 12H).

^13^C-NMR (126 MHz, CD_3_OD): *δ* 26.73, 33.59, 46.75, 52.04, 58.09, 174.96.

ESP-MS: *m*/*z* = 459.28 [M + H. Calcd 458.55].

### Materials for the biological studies

All cell lines used in the research were purchased from ATCC®. Cancer cell lines: THP-1 (ATCC TIB-202 monocyte isolated from peripheral blood from an acute monocytic leukemia patient) was cultured in RPMI-1640 medium, MCF-7 (ATCC HTB-22 human breast adenocarcinoma) cell line in Dulbecco's Modified Eagle Medium (DMEM) enriched with GlutaMAX and HeLa (ATCC CRM-CCL-2 human endothelial cervical cancer) in DMEM enriched with GlutaMAX-1 (ATCC CRL-3243 human dermal microvascular endothelium) cells were grown in an MCDB131 medium supplemented with hydrocortisone (1 μg mL^−1^), l-glutamine (10 mM) and epidermal growth factor (10 ng mL^−1^). The laboratory where the tests were performed is authorized and adapted to work with BSL-2 cell lines.

### Cell culture

All cell lines used in the research were purchased from ATCC®. Cancer cell lines: THP-1 (ATCC TIB-202 monocyte isolated from peripheral blood from an acute monocytic leukemia patient) was cultured in RPMI-1640 medium, MCF-7 (human breast adenocarcinoma) cell line in Dulbecco's Modified Eagle Medium (DMEM) enriched with GlutaMAX and HeLa (ATCC CRM-CCL-2 human endothelial cervical cancer) in DMEM enriched with GlutaMAX. Non-cancerous HMEC-1 (ATCC CRL-3243 human dermal microvascular endothelium) cells were grown in an MCDB131 medium supplemented with hydrocortisone (1 μg mL^−1^), l-glutamine (10 mM) and epidermal growth factor (10 ng mL^−1^). 10% fetal bovine serum (FBS) and streptomycin (100 mg mL^−1^) were added to all cell culture media. Cells were cultured in T-75 culture flasks in the atmosphere containing 5.0% CO_2_ at 37 °C and subcultured every 2–3 days. Cells were used in experiments after obtaining 80–90% confluence. The number of viable cells was determined by the trypan blue exclusion assay using a Invitrogen Countess Automated Cell Counter (Thermo Fisher Scientific, USA).

The laboratory where the tests were performed is authorized and adapted to work with BSL-2 cell lines.

### Cytotoxicity assay

The influence of analyzed dendrimer cores on the adherent cells viability was determined with the use of the MTT assay. Briefly, different concentrations of compounds were added to the 96-well plates containing cells at the density of 1.5 × 10^4^ cells per well in medium. Cells were incubated with compounds in final concentrations: 0.1, 1, 10, 100 μM for 24 and 48 h at 37 °C, 5% CO_2_. After the incubation time cells were washed once with PBS, and 50 μL of a 0.5 mg mL^−1^ solution of MTT in PBS was added to each well and cells were further incubated under normal culture conditions for 3 h. After incubation the MTT solution was removed and the obtained formazan crystals were dissolved in DMSO (100 μL per well). The conversion of the tetrazolium salt (MTT) to a formazan by mitochondrial and cytosolic dehydrogenases is a marker of cell viability. Before the measurement plates were shaken for 1 min and the absorbance at 570 nm was measured on PowerWave HT microplate reader (BioTek, USA).

To estimate the cytotoxic activity of analyzed dendrimer cores on the suspension cells, resazurin assay was performed. Cells were seeded into 96-well black plates at a density of 1.5 × 10^4^ cells per well and treated with increasing concentrations of analyzed compounds (0.1–100 μM) for 24 and 48 hours. Following the incubation, resazurin was added to the culture medium to a final concentration of 12.5 μg mL^−1^ and the plates were incubated at 37 °C in darkness for 2 hours. Fluorescence was measured at 530 nm excitation and 590 nm emission using PowerWave HT microplate reader (BioTek, USA).

### Reactive oxygen species (ROS) assay

Cells were seeded on 96-well black plates at a density of 1.5 × 10^4^ cells per well in 100 μL of an appropriate medium. After treatment with analyzed compounds in concentration 100 μM, cells were incubated for 3 and 24 h, and then stained with 50 μL of 2 μM H2DCFDA for 15 min in growing conditions. Then the dye solution was removed and cells were washed twice with PBS. Fluorescence (*λ*_ex_ = 485 nm, *λ*_em_ = 530 nm) was measured using the PowerWave HT microplate reader (BioTek, USA).

### Mitochondrial membrane potential (Δ*Ψ*_m_) assay

Cells were seeded on 96-well black plates at a density of 1.5 × 104 cells per well in 100 μL of an appropriate medium. After treatment with analyzed compounds in concentration 100 μM, cells were incubated for 3 and 24 h, and then 50 μL of 5 μM JC-1 was added to each well and incubated for 30 min in growing conditions. The dye was removed, cells were washed twice with PBS and then 50 μL of PBS was added to each well. Measurements (ex: 530 nm, em: 590 nm//*λ*_ex_ = 485 nm, *λ*_em_ = 540) were performed using the PowerWave HT microplate reader (BioTek, USA).

### Apoptotic and necrotic cell fraction analysis

Cells were seeded in 24-well transparent plates at a density of 2 × 10^5^ cells per well in 1 mL of an appropriate medium and after 24 hours treated with analyzed compounds at a concentration of 100 μM. After 24 h incubation, cells were trypsinised, and then washed with PBS and suspended in 200 μL of binding buffer (delivered from the producer). The mixture consisting of 5 μL of Annexin V conjugated with FITC and 4 μL of propidium iodide was added to cell suspension. Samples were incubated at room temperature for 20 min in the dark. Measurement of fluorescence intensity was performed by a Becton Dickinson LSR II flow cytometer. The control apoptosis was induced by camptothecin (80 μM) and necrosis was induced by ethanol (data not shown). The data were recorded for a total of 10 000 events per sample.

### Cell cycle analysis

Cells were seeded in 12-well transparent plates at a density of 4 × 10^5^ cells per well in 1 mL of an appropriate medium. After treatment with compounds in concentration 100 μM, cells were trypsinised, collected and fixed in ice-cold 96% ethanol for 24 h. Then, cells were washed with PBS and incubated for 30 min at 37 °C in 500 μL of staining solution containing 10 mM Tris–HCl (pH = 7.5), 5 mM magnesium chloride, 10 μg mL^−1^ propidium iodide and 10 μg mL^−1^ ribonuclease A. After this time samples were analysed by a Becton Dickinson LSRII flow cytometer. The data were recorded for a total of 10 000 events per sample. Data were plotted by using Flowing Software 2.5.1 (Turku Centre for Biotechnology, University of Turku, Finland).

### Statistical analysis

Data was expressed as mean ± SD. Analysis of variance (ANOVA) with the Tukey post hoc test was used for results comparison. All statistics were counted using the Statistica software (StatSoft, Tulsa, USA), and *p* < 0.05 was considered significant.

## Data availability

The raw data are available in the ESI material†

## Author contributions

CBH carried out the synthesis, AJ, MD, MM did the biological work and BK-M, JBC and AJ planned the study and analyzed the results.

## Conflicts of interest

There are no conflicts to declare.

## Supplementary Material

RA-014-D4RA02020K-s001
