# Supplementary material for: Core-size and geometry versus toxicity in small amino terminated PAMAM dendrimers
Source: RSC Adv. 2024 Sep 10;14(39):28684–92. doi: 10.1039/d4ra02020k (PMC11384932; doi:10.1039/d4ra02020k)

Supplementary Information (SI) for RSC Advances.
This journal is © The Royal Society of Chemistry 2024

Supplementary material

Core-size and geometry versus toxicity in small amino terminated PAMAM dendrimers.

Claus Bøge Hansen[a], Anna Janaszewska[b], Monika Dąbrzalska[b], Monika Marcinkowska[b], Barbara Klajnert-Maculewicz[b], Jørn Bolstad Christensen[a*]

[a] *Department of Chemistry, Faculty of Science, University of Copenhagen, Thorvaldsensvej 40, DK-1871 Frederiksberg, Denmark; jbc@chem.ku.dk*
[b] *Department of General Biophysics, Faculty of Biology and Environmental Protection, University of Lodz, 141/143 Pomorska Street, 90-236 Lodz, Poland*

NMR- and ESMS-spectra of the compounds synthesized

Compound 1: Page 2-5

Compound 2: Page 6 – 9

Compound 3: Page 10 – 13

Compound 4: Page 14 – 17

Compound 5: Page 18 – 21

Compound 6: Page 22 – 25

Compound 7: Page 26 – 29

Compound 8: Page 30 – 33

Compound 9: Page 34 – 37

Compound 10: Page 38 – 41

Compound 11: Page 42 – 45

Compound 12: Page 46 – 49

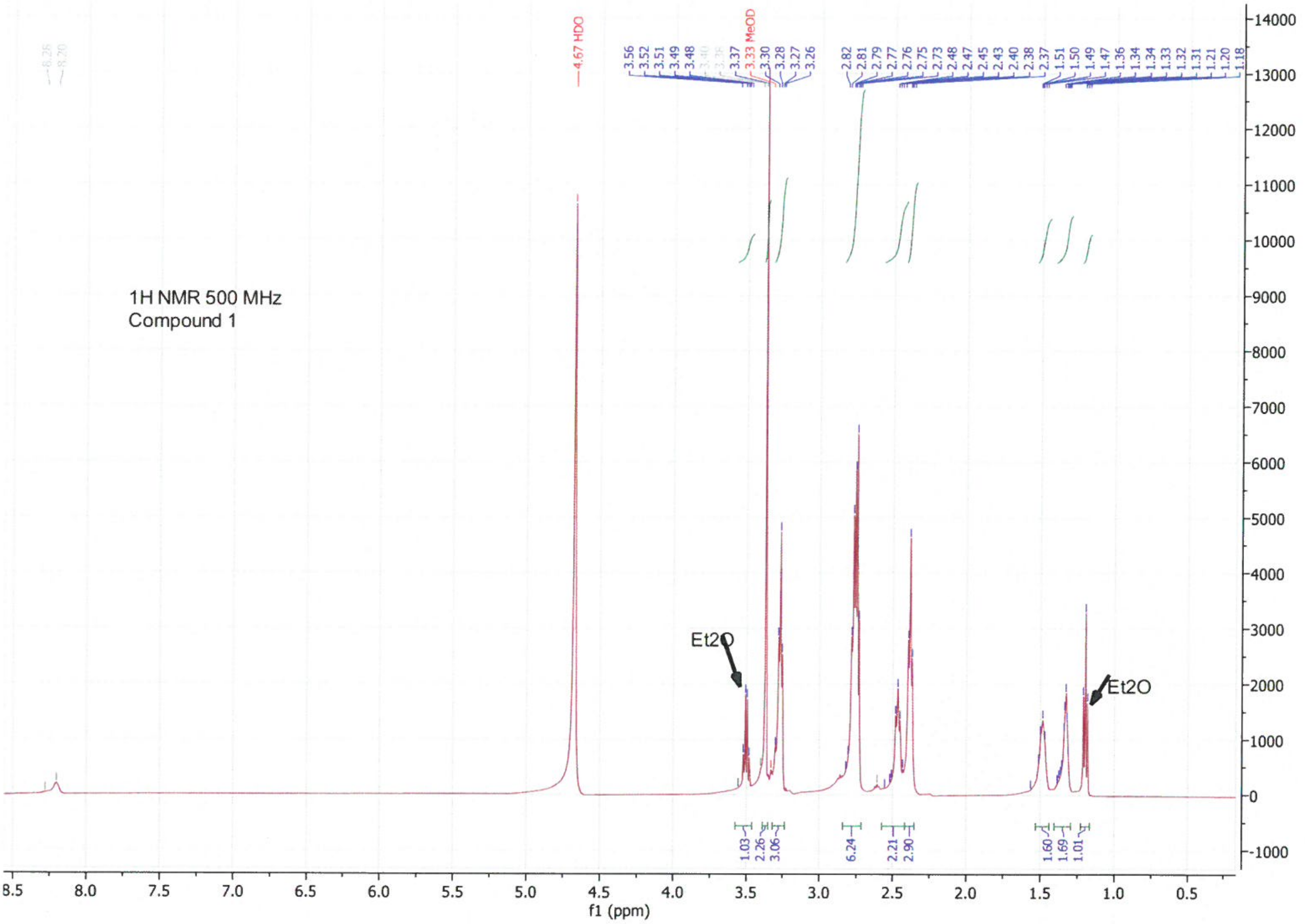
1H NMR 500 MHz
Compound 1
4.67 HDO
3.33 MeOD
Et2O
Et2O
f1 (ppm)

COSY NMR 500 MHz
Compound 1

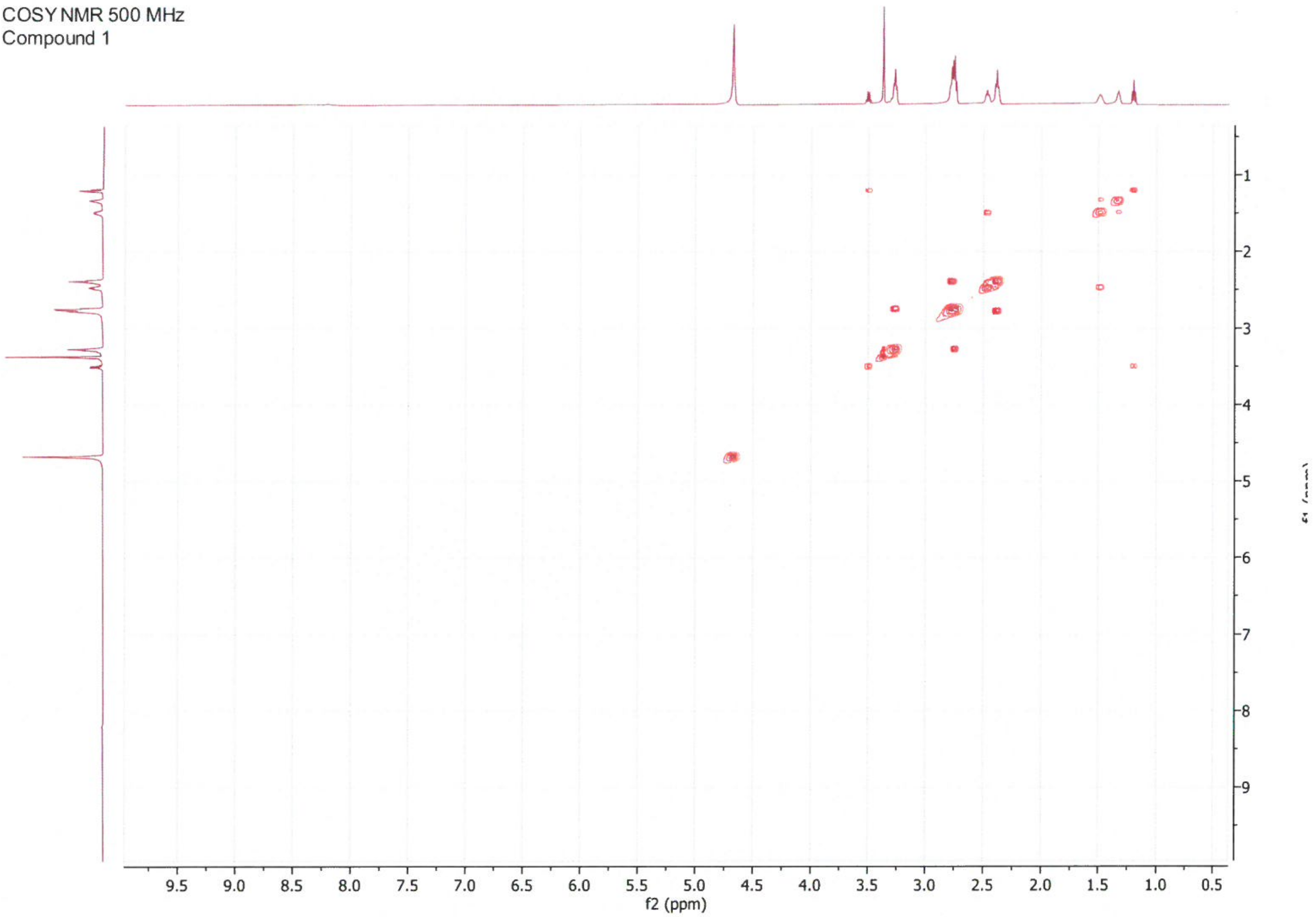

3

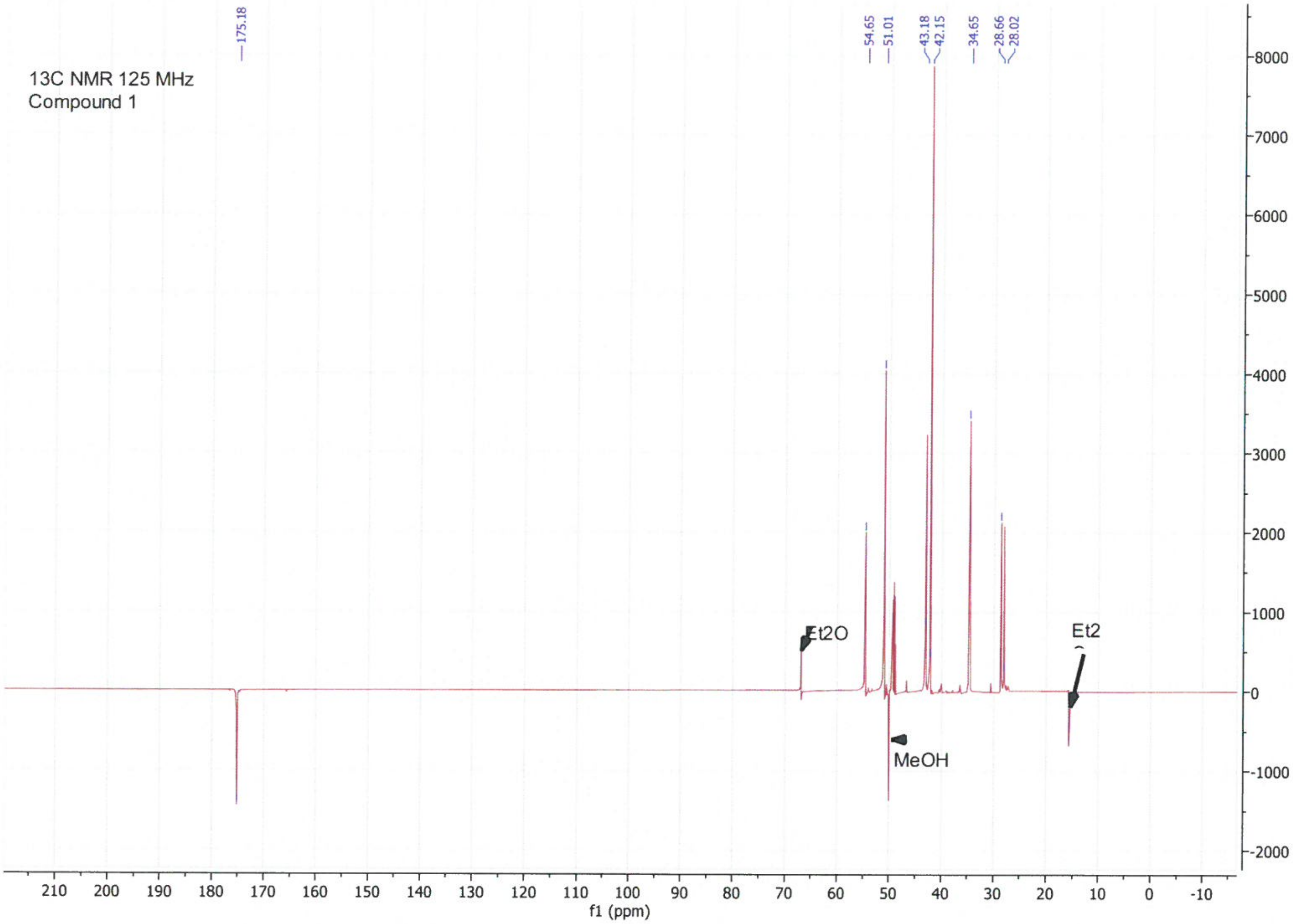
13C NMR 125 MHz
Compound 1
175.18
54.65
51.01
43.18
42.15
34.65
28.66
28.02
Et2O
Et2
MeOH
f1 (ppm)

Compound 1

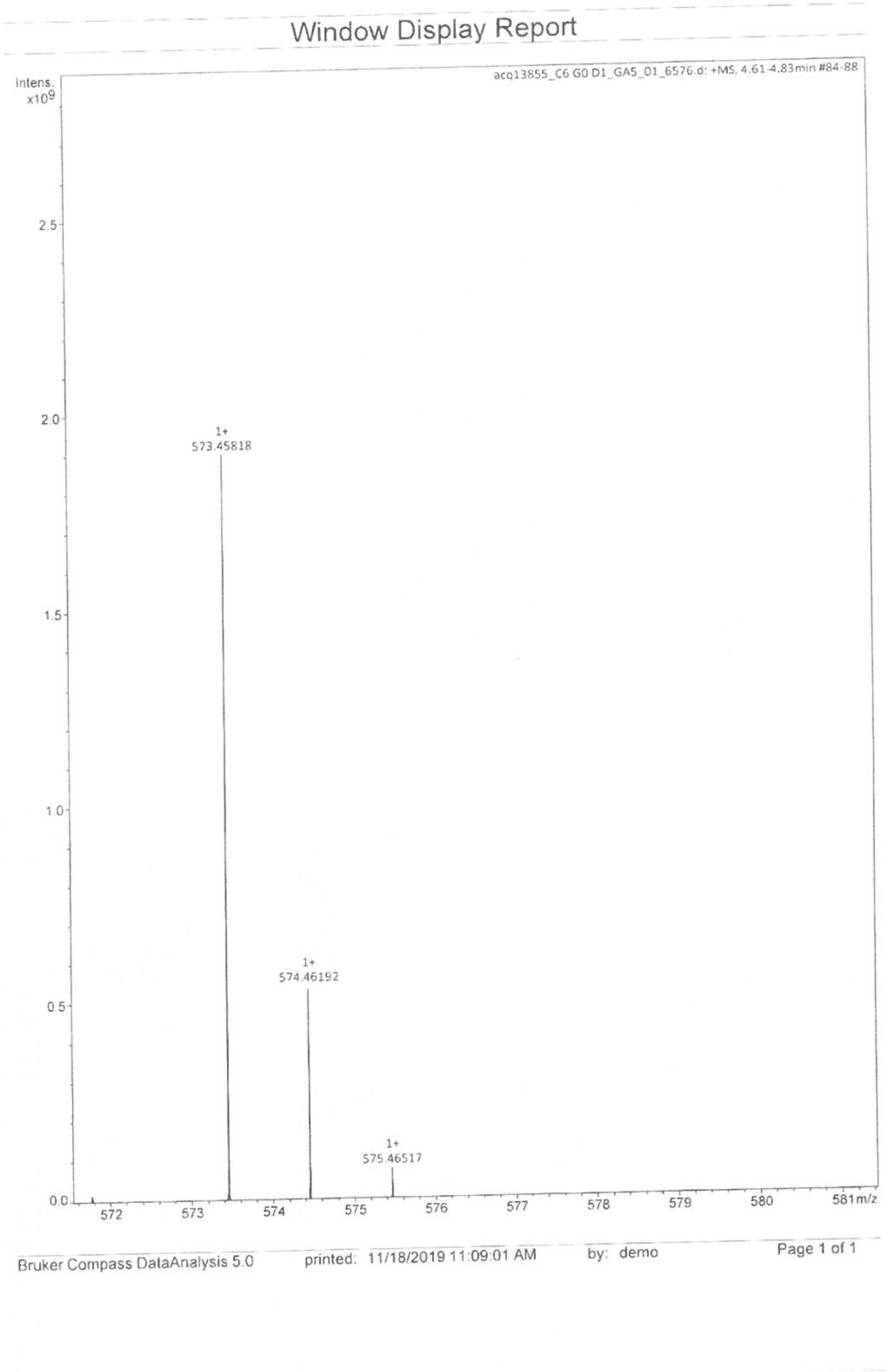
Window Display Report
acq13855_C6 G0 D1_GA5_01_6576.d: +MS, 4.61-4.83min #84-88
Intens. x10^9
1+
573.45818
1+
574.46192
1+
575.46517
m/z
Bruker Compass DataAnalysis 5.0
printed: 11/18/2019 11:09:01 AM
by: demo
Page 1 of 1

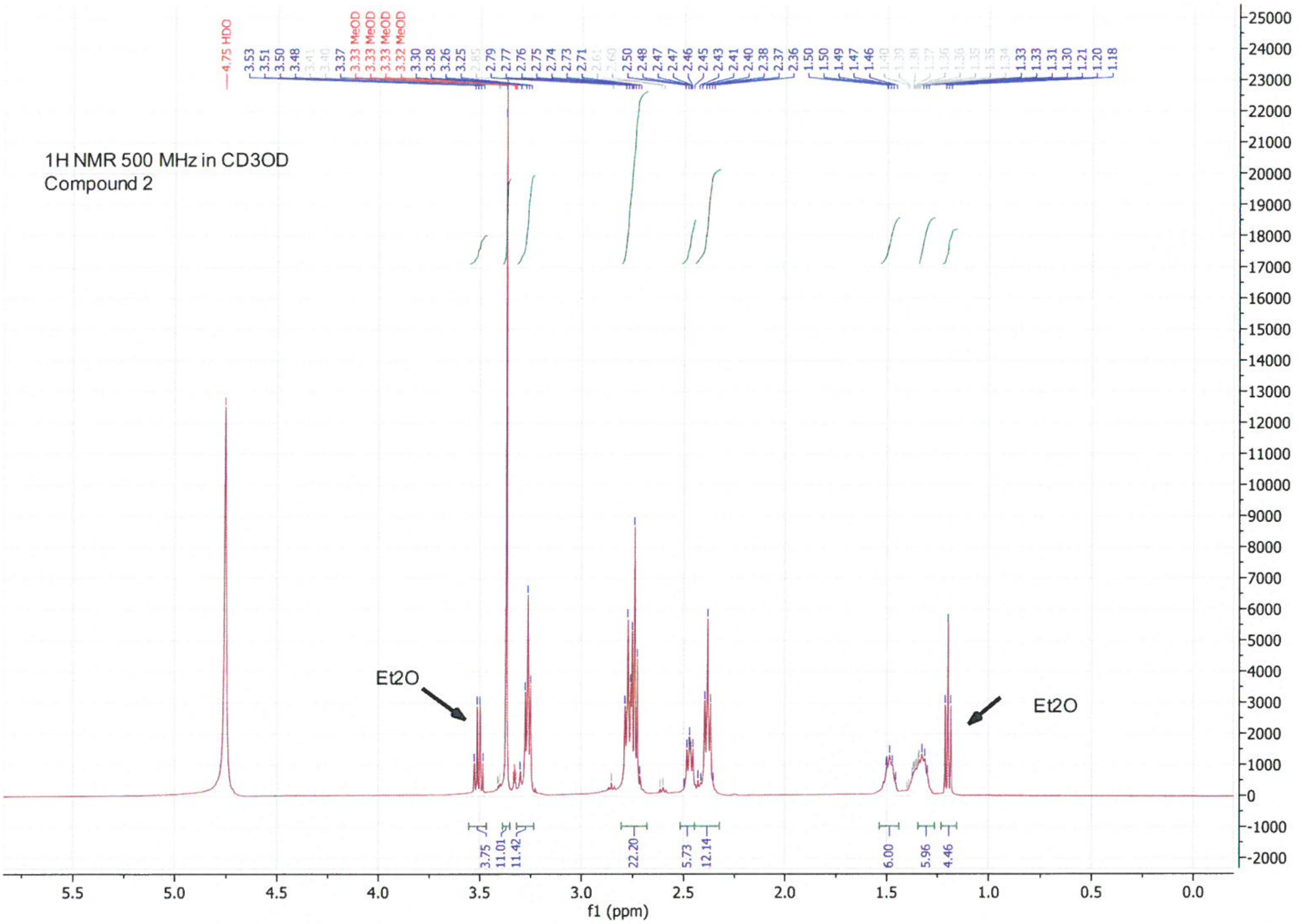
1H NMR 500 MHz in CD3OD
Compound 2
Et2O
Et2O
f1 (ppm)

COSY NMR 500 MHz in CD3OD
Compound 2

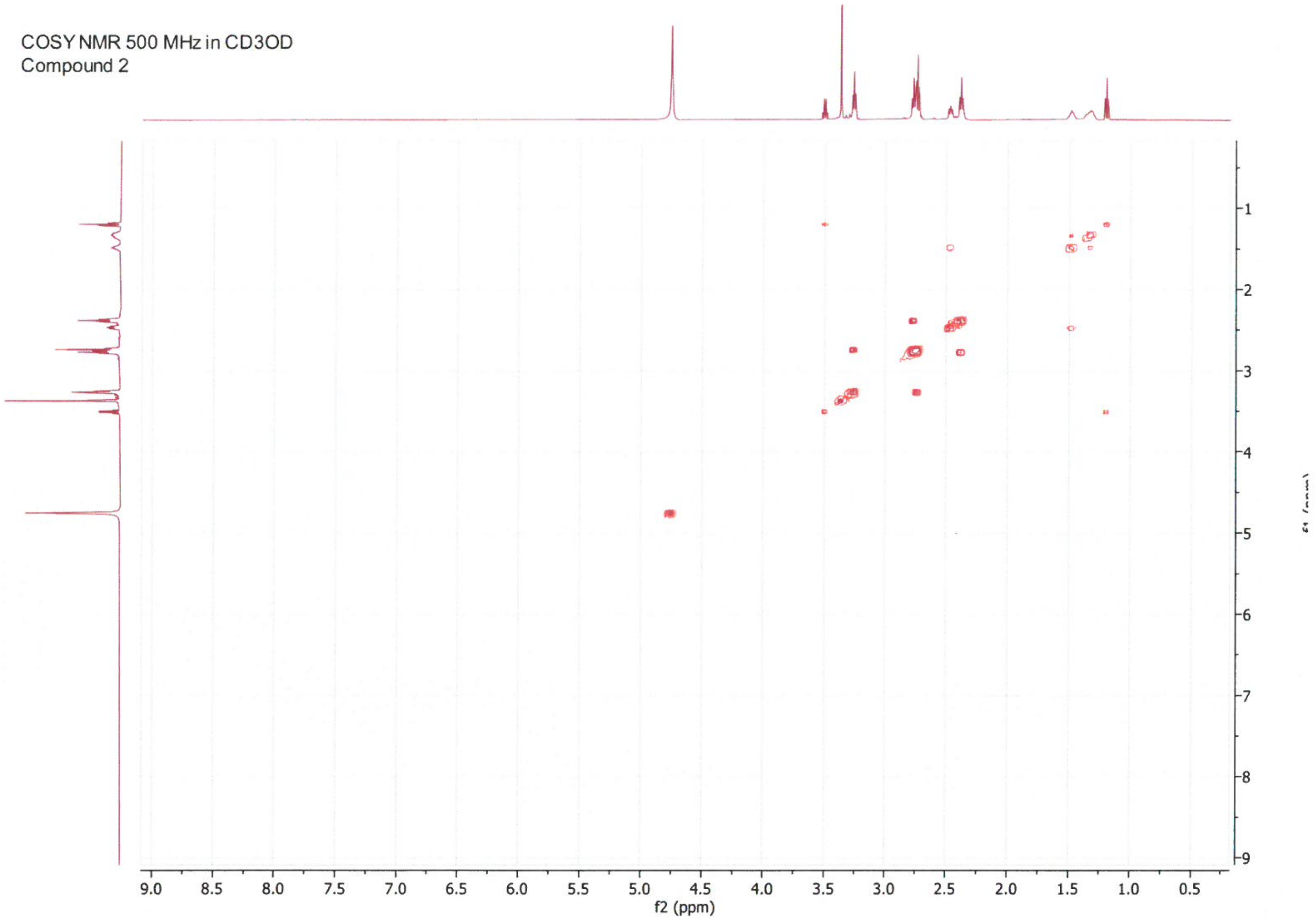

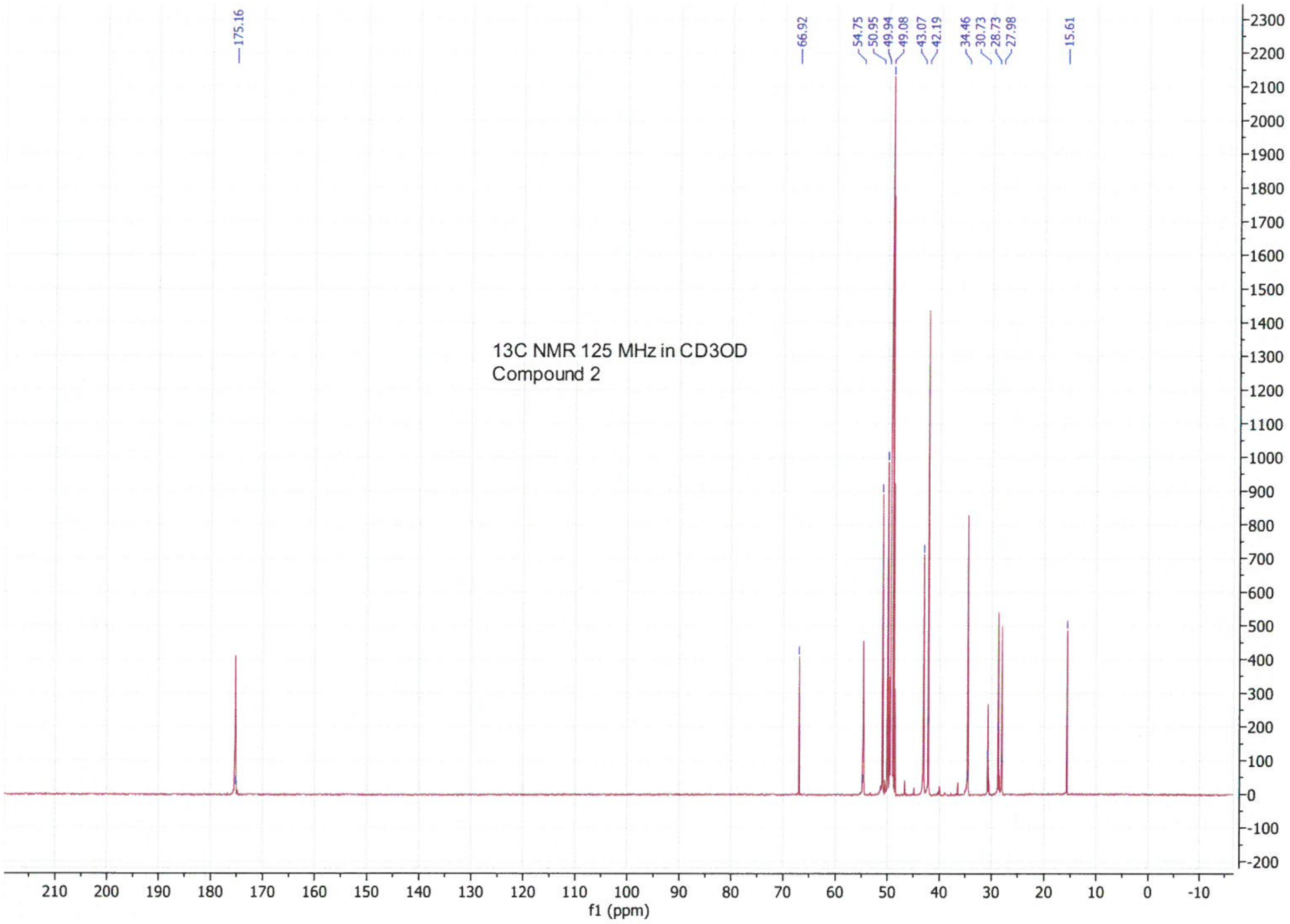
13C NMR 125 MHz in CD3OD
Compound 2
175.16
66.92
54.75
50.95
49.94
49.08
43.07
42.19
34.46
30.73
28.73
27.98
15.61
f1 (ppm)

8

Compound 2

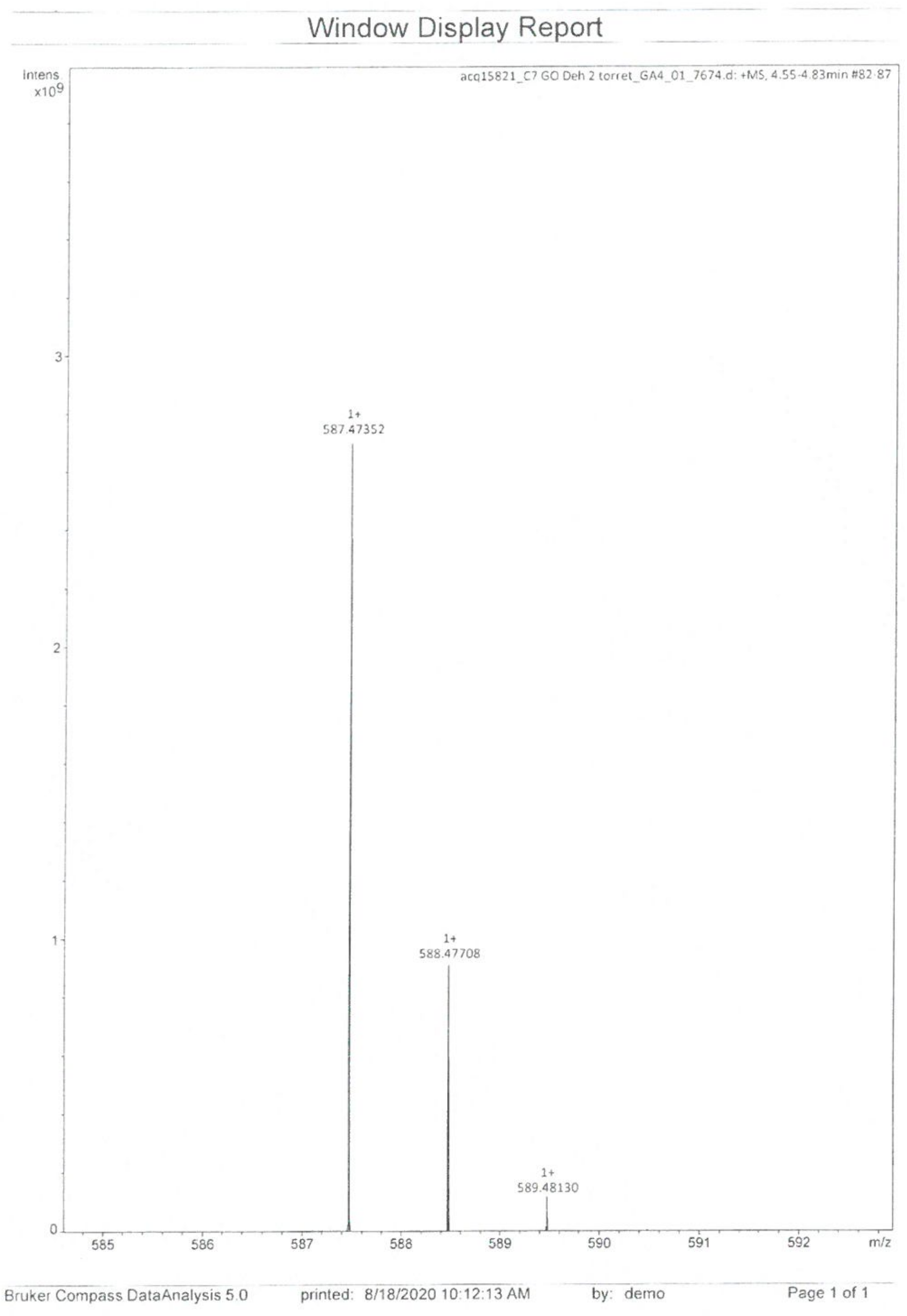
Window Display Report
Intens
x10^9
acq15821_C7 GO Deh 2 torret_GA4_01_7674.d: +MS, 4.55-4.83min #82-87
3
2
1
0
1+
587.47352
1+
588.47708
1+
589.48130
585
586
587
588
589
590
591
592
m/z
Bruker Compass DataAnalysis 5.0
printed: 8/18/2020 10:12:13 AM
by: demo
Page 1 of 1

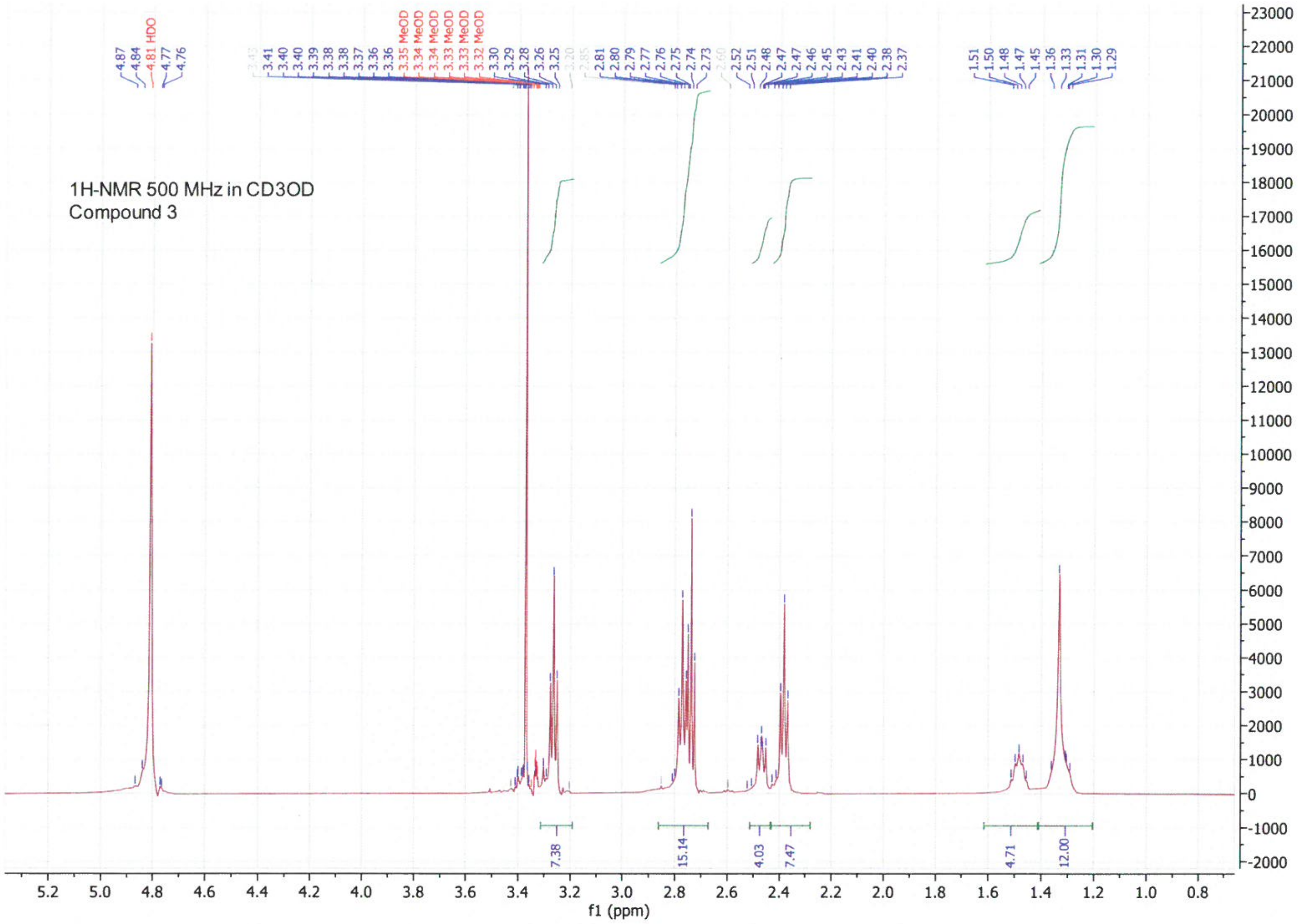

10

COSY NMR 500 MHz in CD3OD
Compound 3

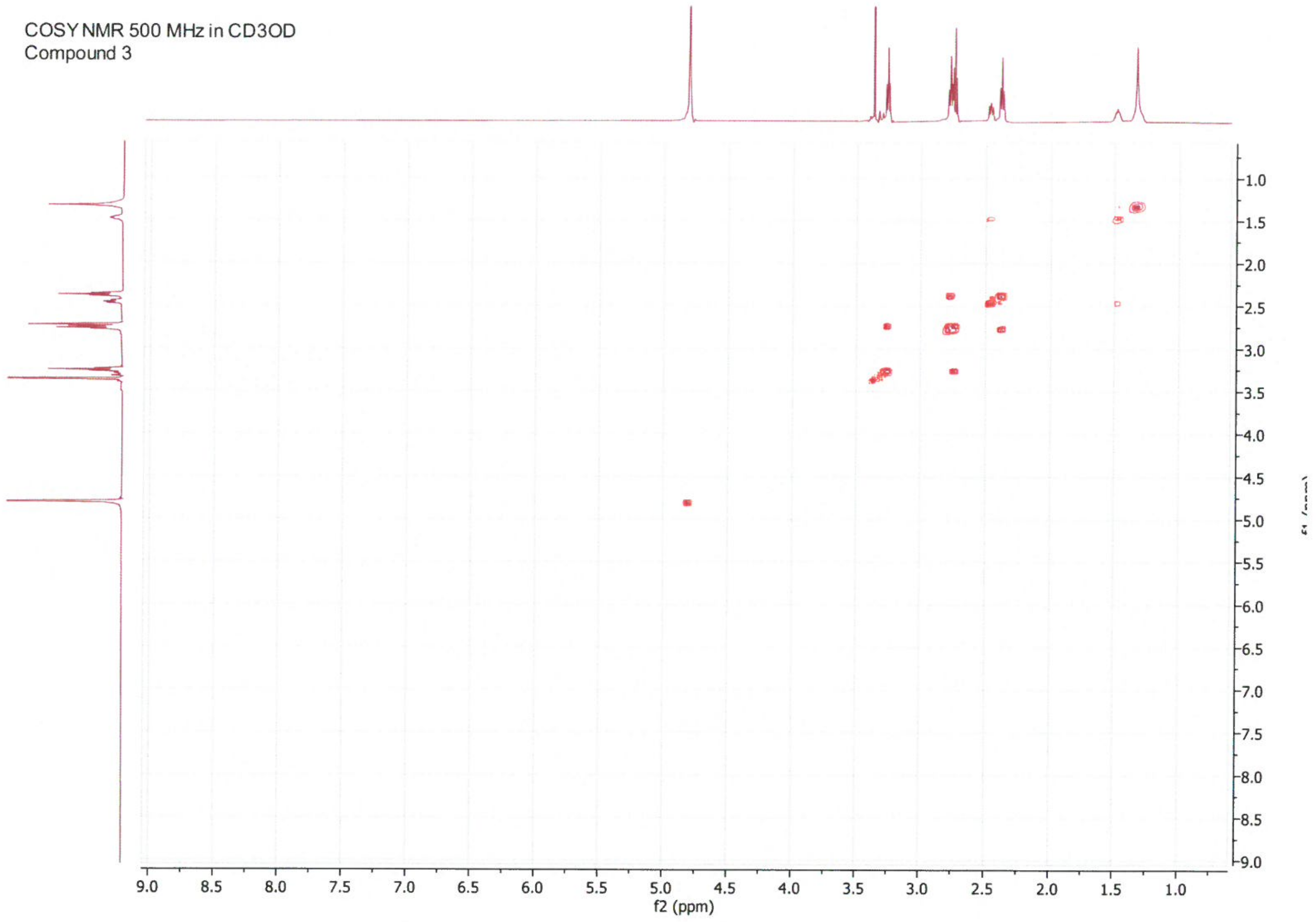

11

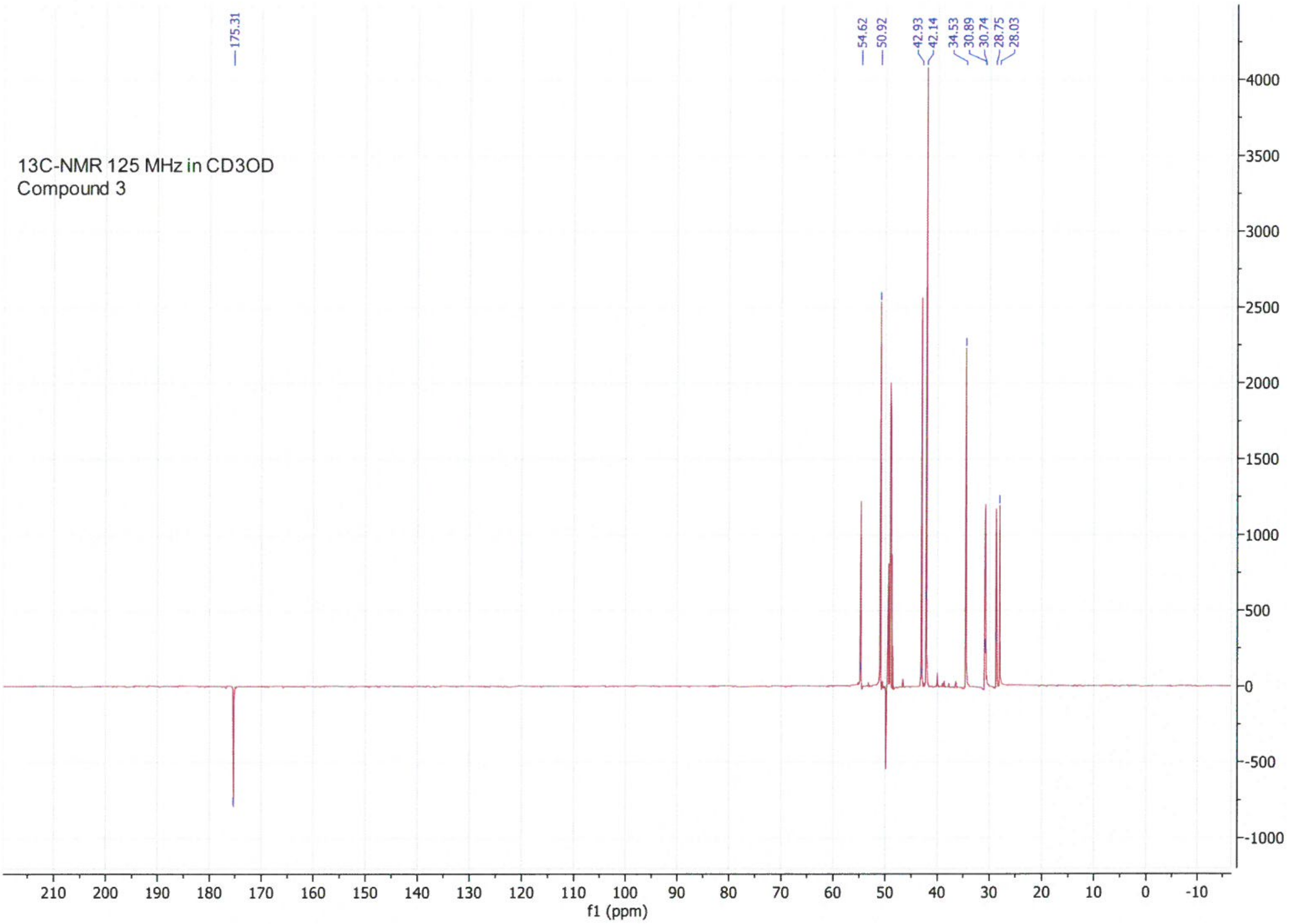
175.31
54.62
50.92
42.93
42.14
34.53
30.89
30.74
28.75
28.03
13C-NMR 125 MHz in CD3OD
Compound 3
4000
3500
3000
2500
2000
1500
1000
500
0
-500
-1000
210 200 190 180 170 160 150 140 130 120 110 100 90 80 70 60 50 40 30 20 10 0 -10
f1 (ppm)

Compound 3

Window Display Report

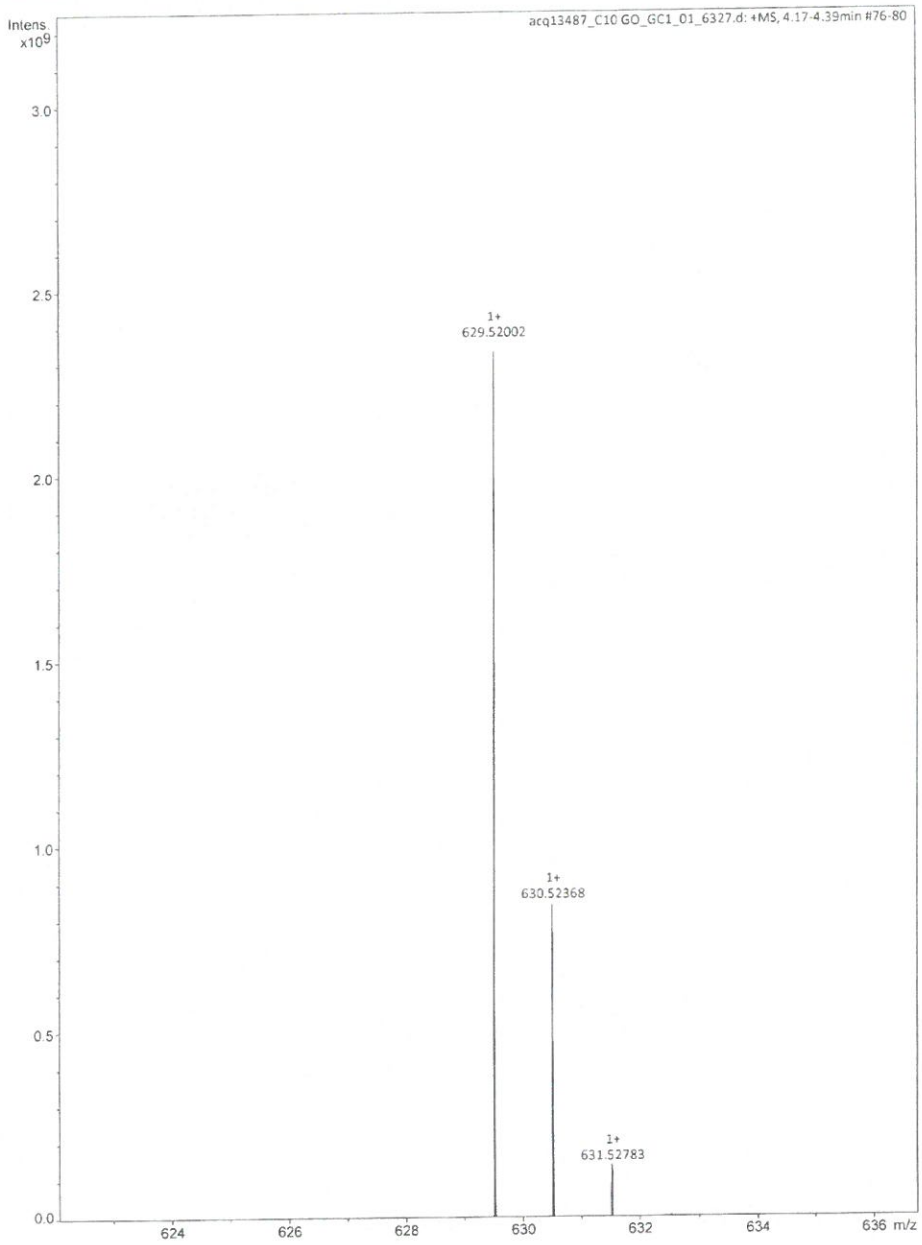

Bruker Compass DataAnalysis 5.0 printed: 10/9/2019 9:36:48 AM by: demo Page 1 of 1

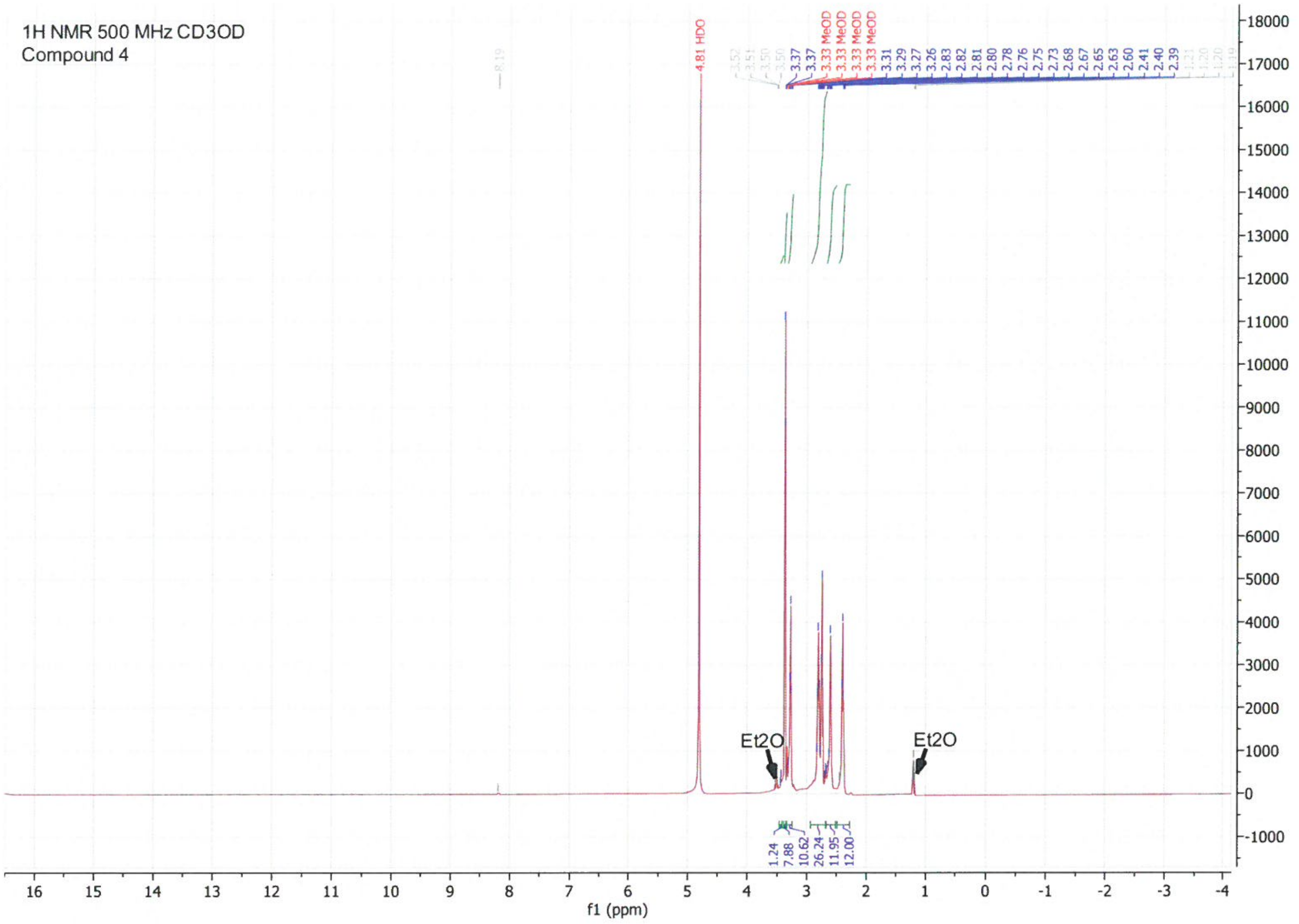

1H NMR 500 MHz CD3OD
Compound 4
4.81 HDO
Et2O
Et2O
f1 (ppm)

COSY NMR 500 MHz CD3OD
Compound 4

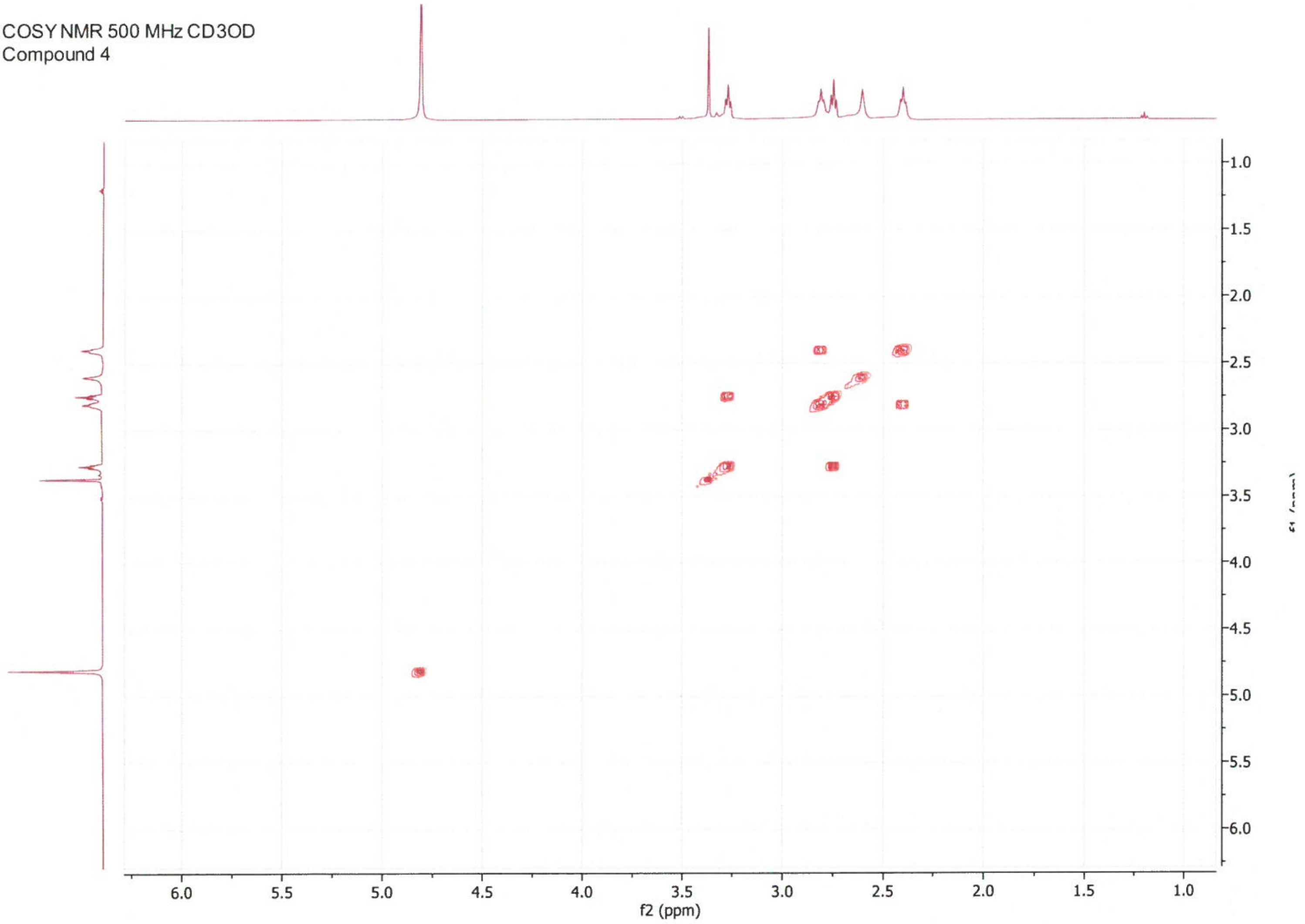

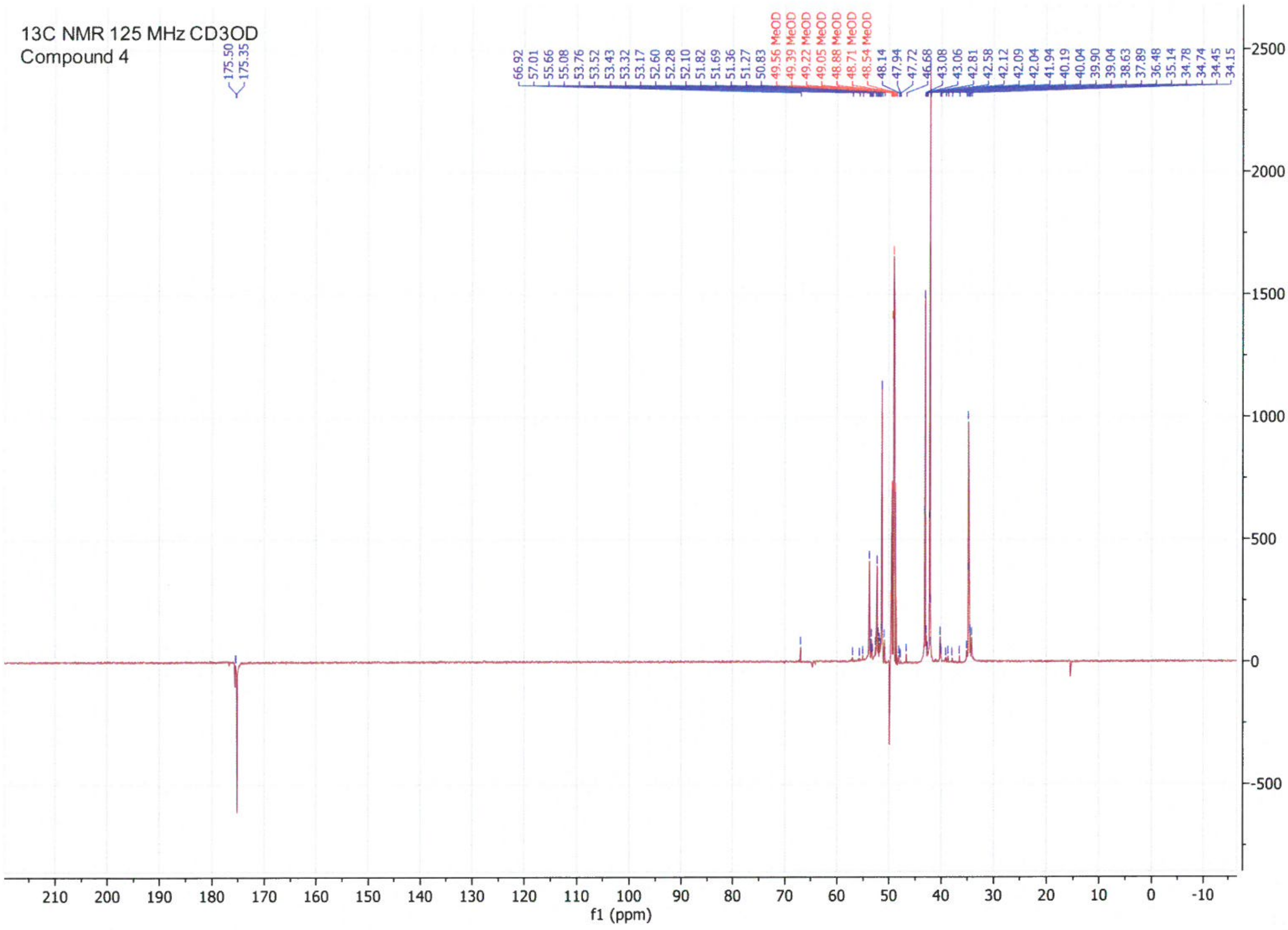
13C NMR 125 MHz CD3OD
Compound 4
175.50
175.35
66.92
57.01
55.66
55.08
53.76
53.52
53.43
53.32
53.17
52.60
52.28
52.10
51.82
51.69
51.36
51.27
50.83
49.56 MeOD
49.39 MeOD
49.22 MeOD
49.05 MeOD
48.88 MeOD
48.71 MeOD
48.54 MeOD
48.14
47.94
47.72
46.68
43.08
43.06
42.81
42.58
42.12
42.09
42.04
41.94
40.19
40.04
39.90
39.04
38.63
37.89
36.48
35.14
34.78
34.74
34.45
34.15
f1 (ppm)

Compound 4

Window Display Report

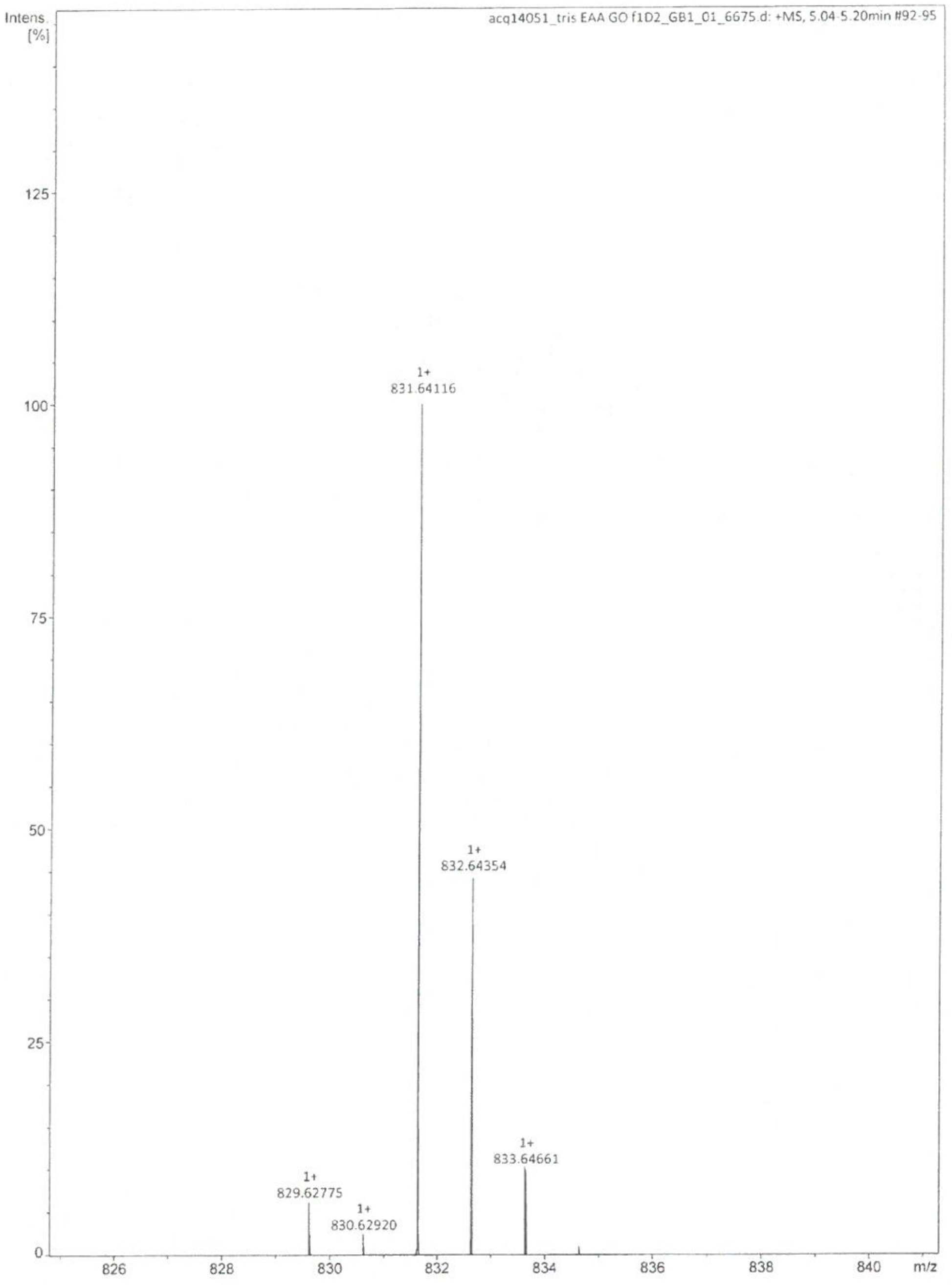

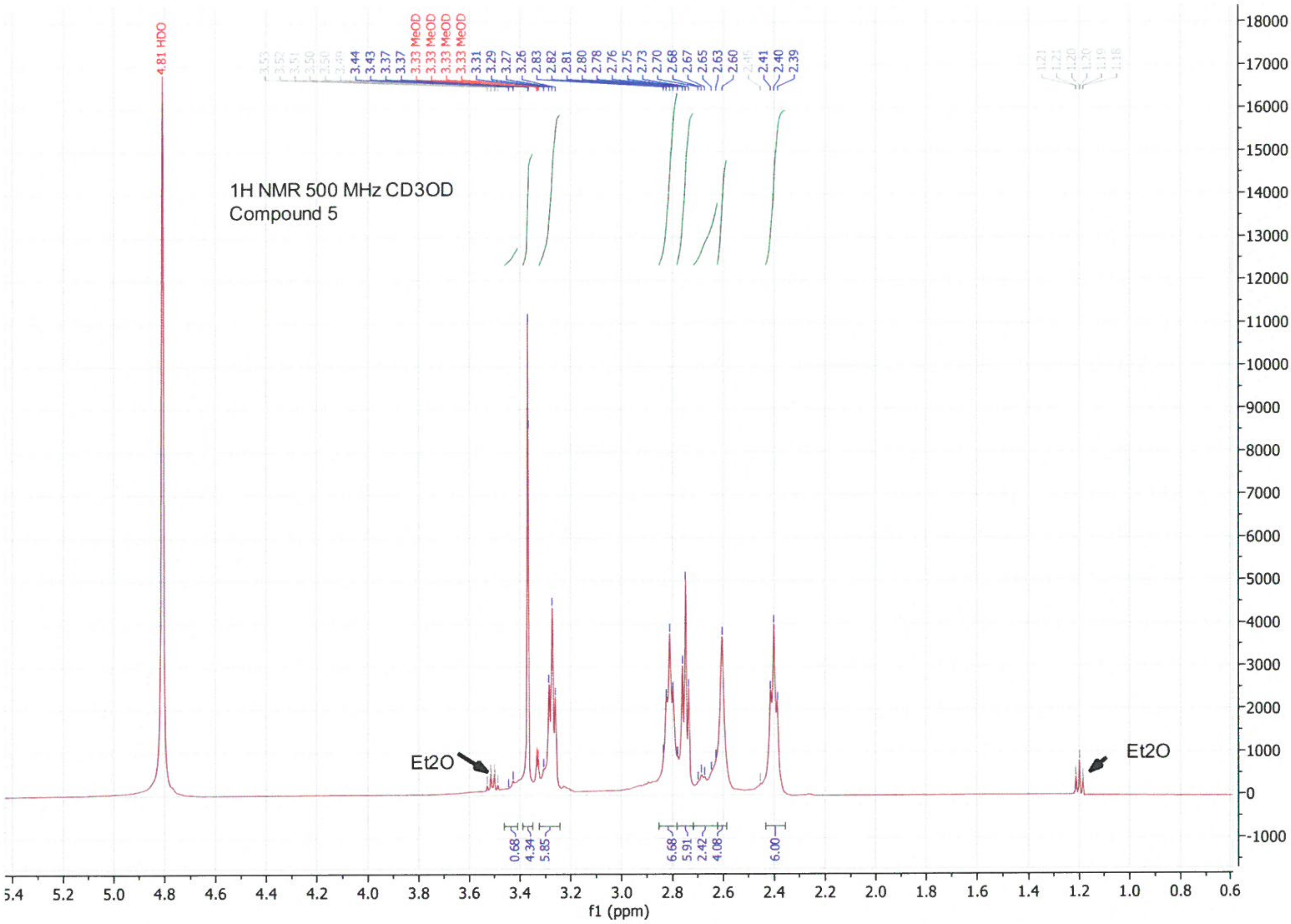
1H NMR 500 MHz CD3OD
Compound 5
4.81 HDO
3.33 MeOD
Et2O
Et2O
f1 (ppm)

COSY NMR 500 MHz CD3OD
Compound 5

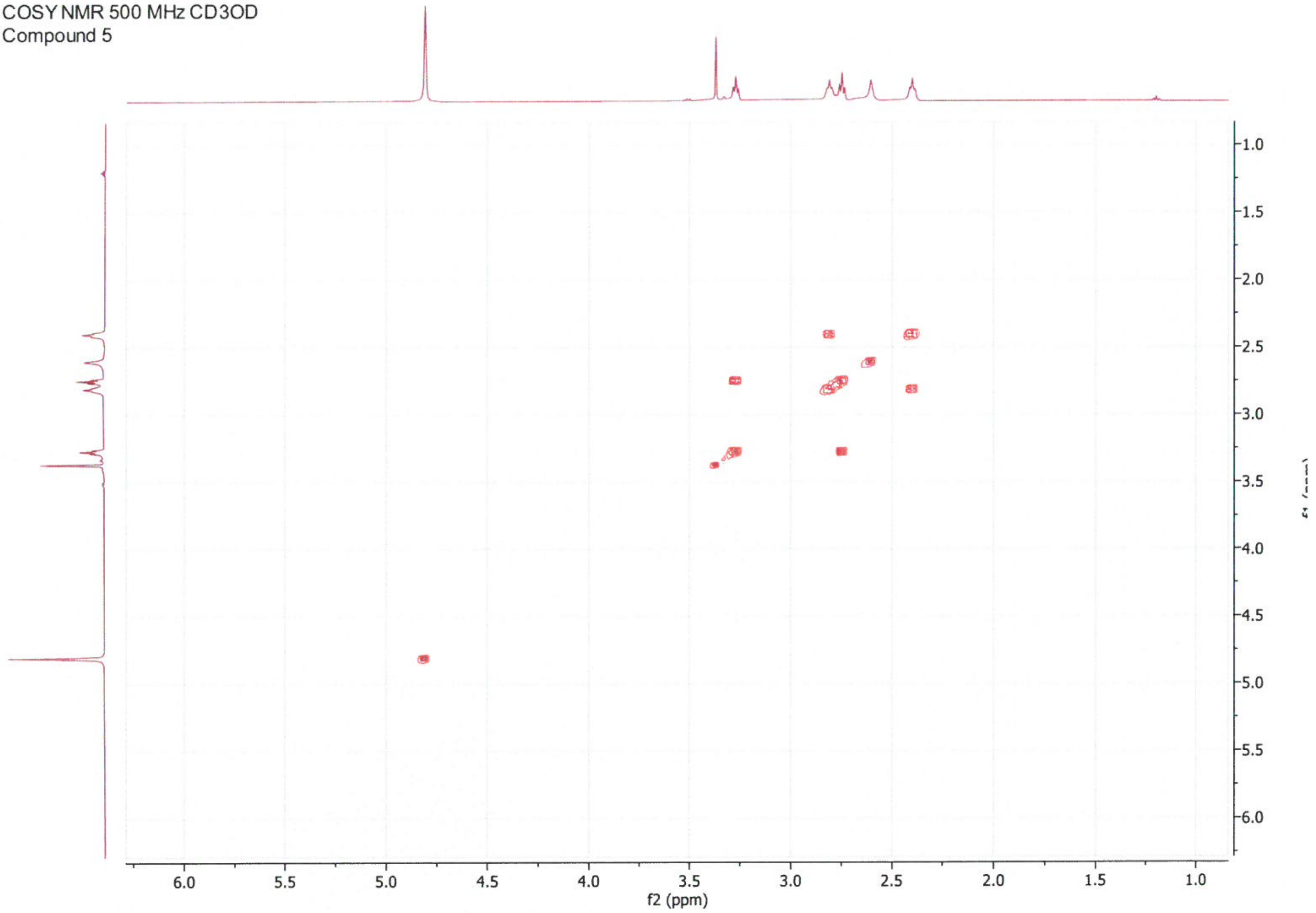

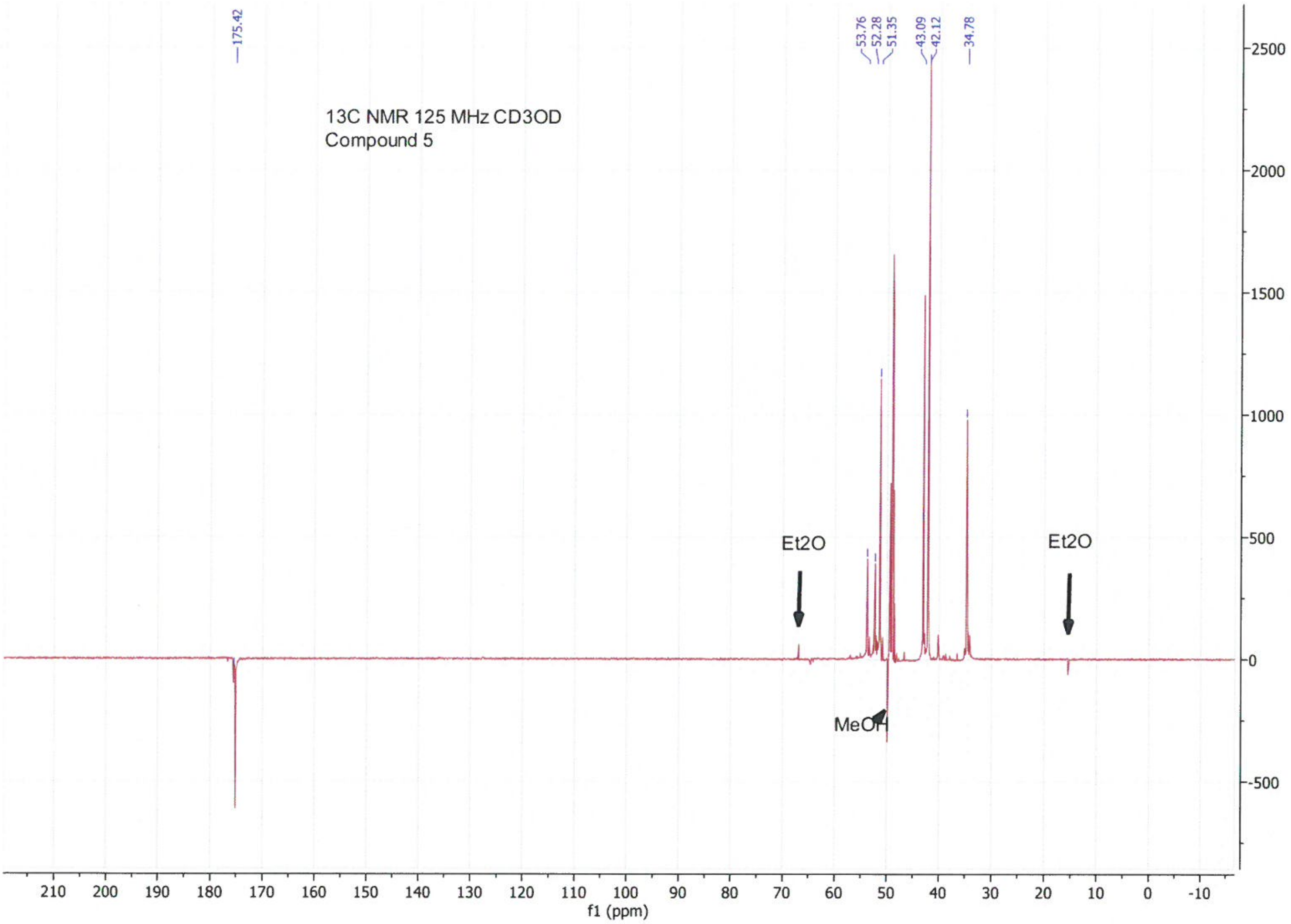
13C NMR 125 MHz CD3OD
Compound 5
175.42
53.76
52.28
51.35
43.09
42.12
34.78
Et2O
Et2O
MeOH
f1 (ppm)

Compound 5

Window Display Report

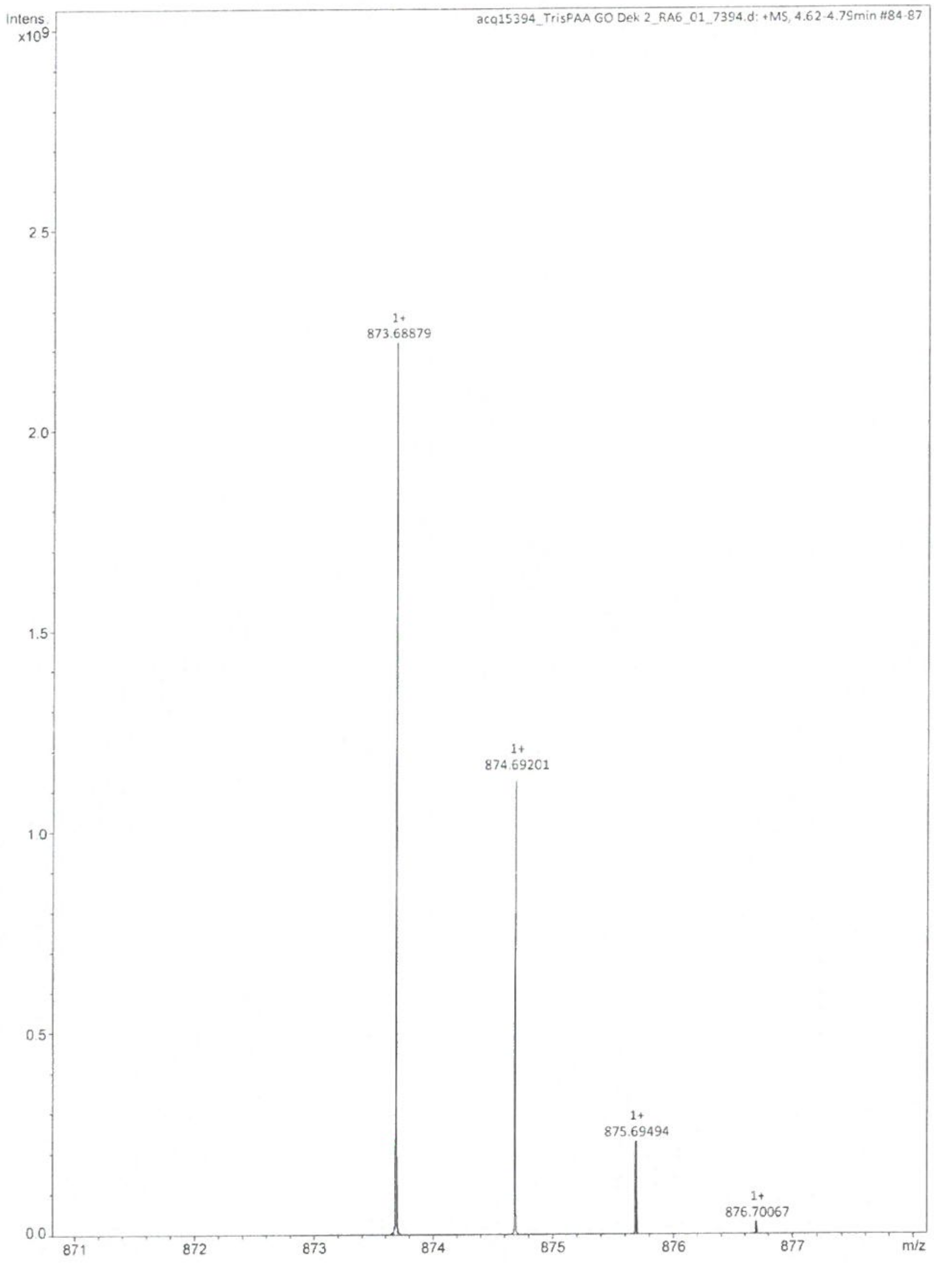

Bruker Compass DataAnalysis 5.0 printed: 7/14/2020 10:04:03 AM by: demo Page 1 of 1

1H NMR 500 MHz CD3OD
Compound 6

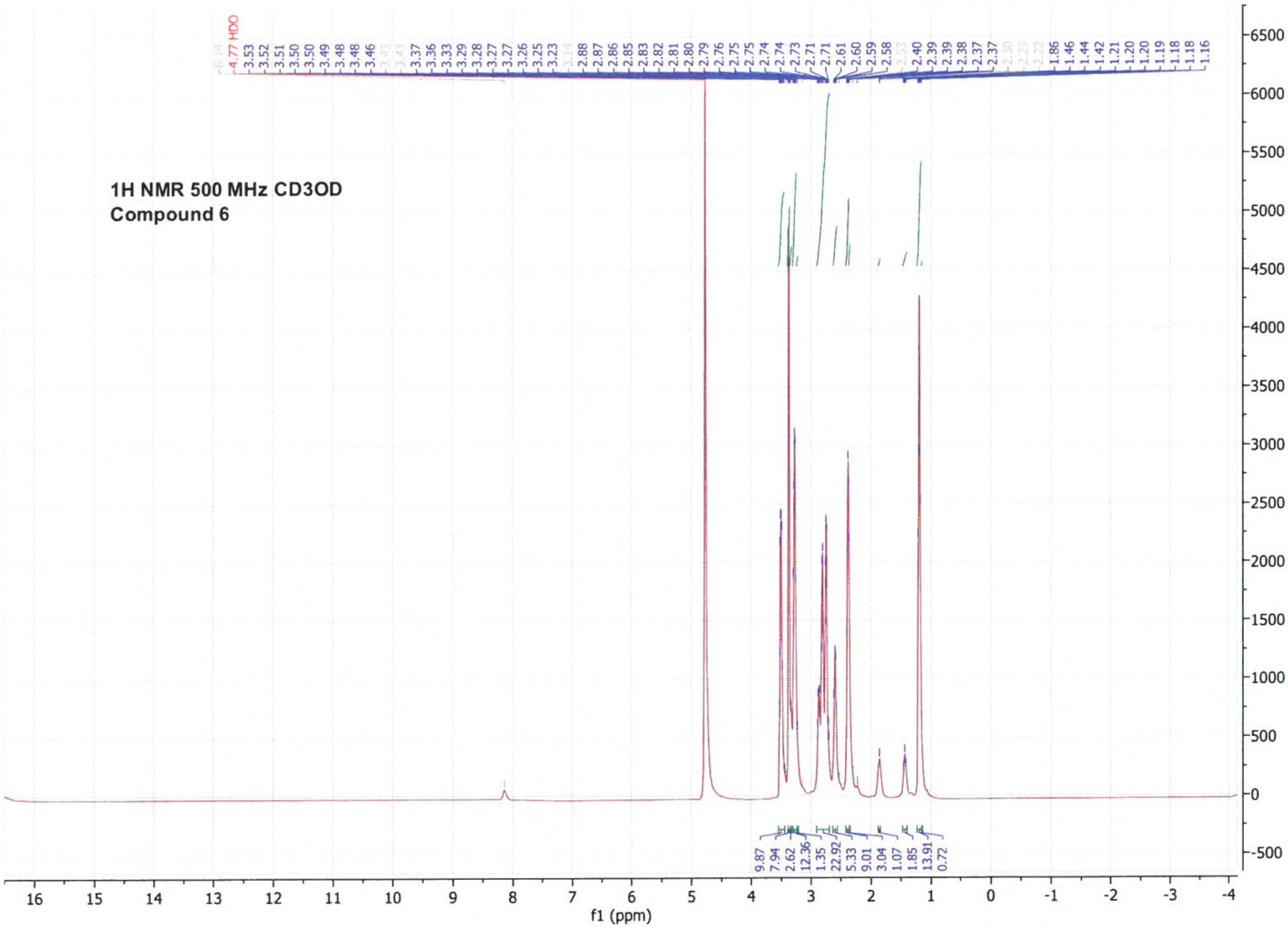

COSY NMR 500 MHz CD3OD
Compound 6

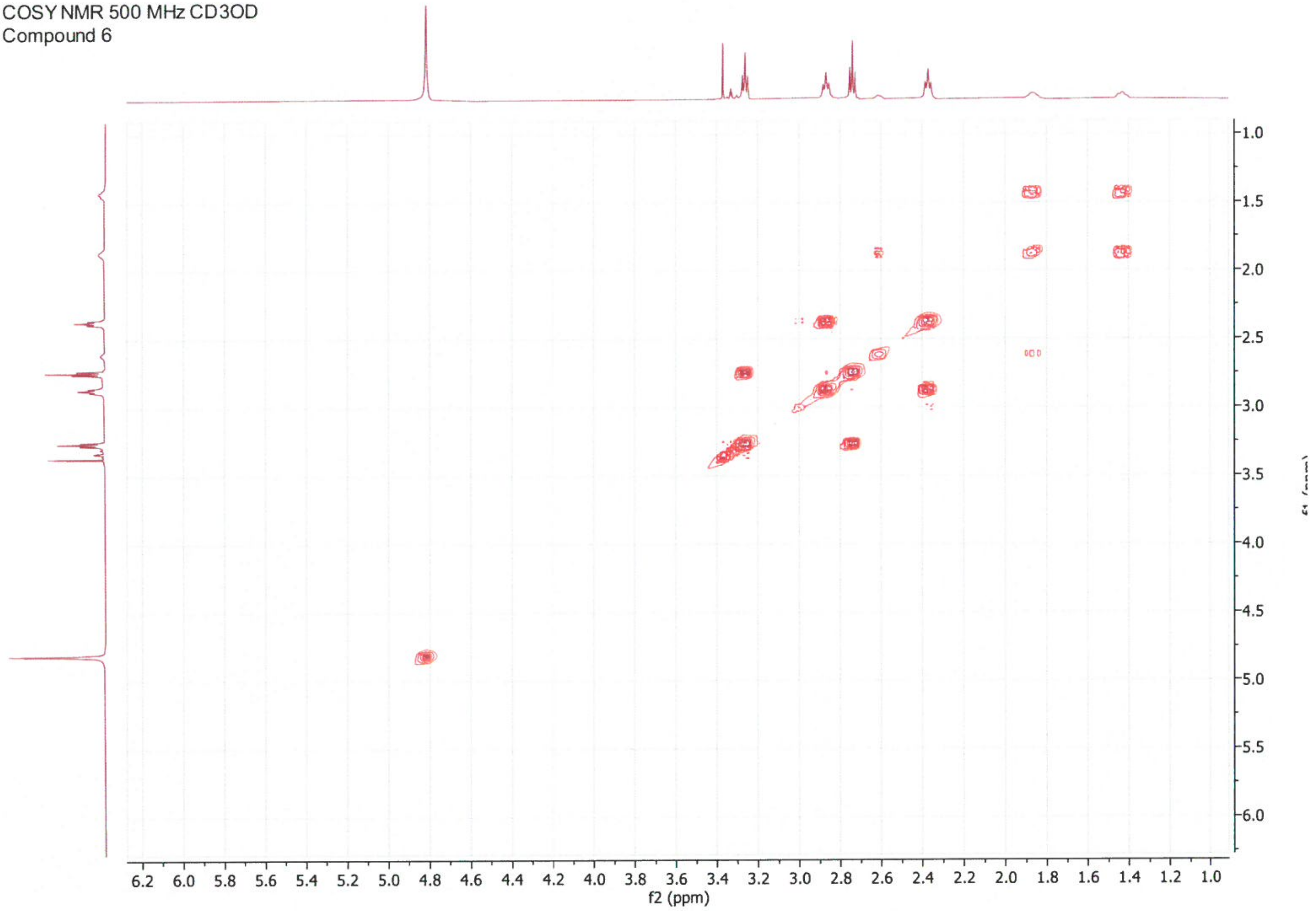

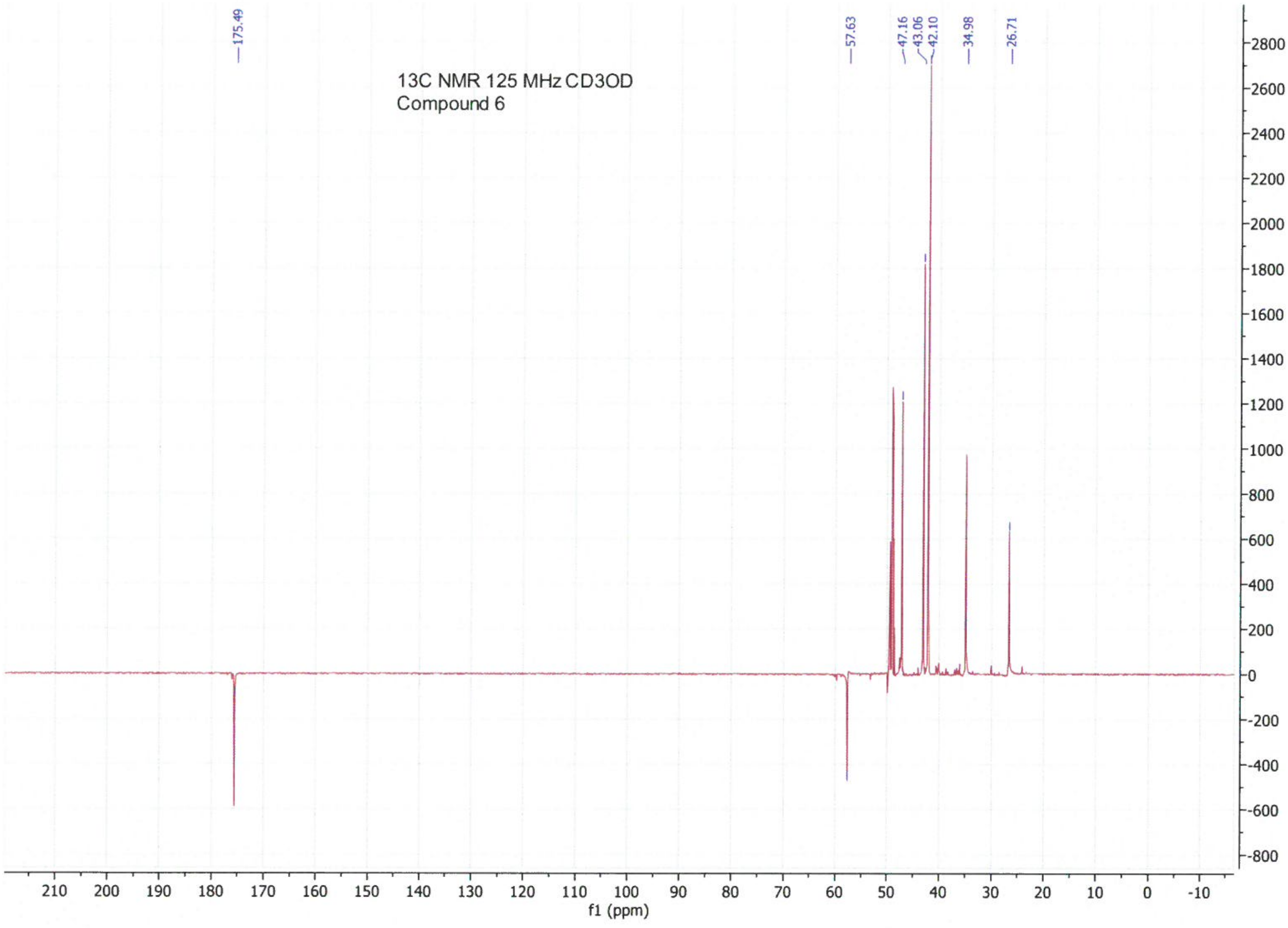

175.49
57.63
47.16
43.06
42.10
34.98
26.71
13C NMR 125 MHz CD3OD
Compound 6
2800
2600
2400
2200
2000
1800
1600
1400
1200
1000
800
600
400
200
0
-200
-400
-600
-800
210 200 190 180 170 160 150 140 130 120 110 100 90 80 70 60 50 40 30 20 10 0 -10
f1 (ppm)

Compound 6

Window Display Report

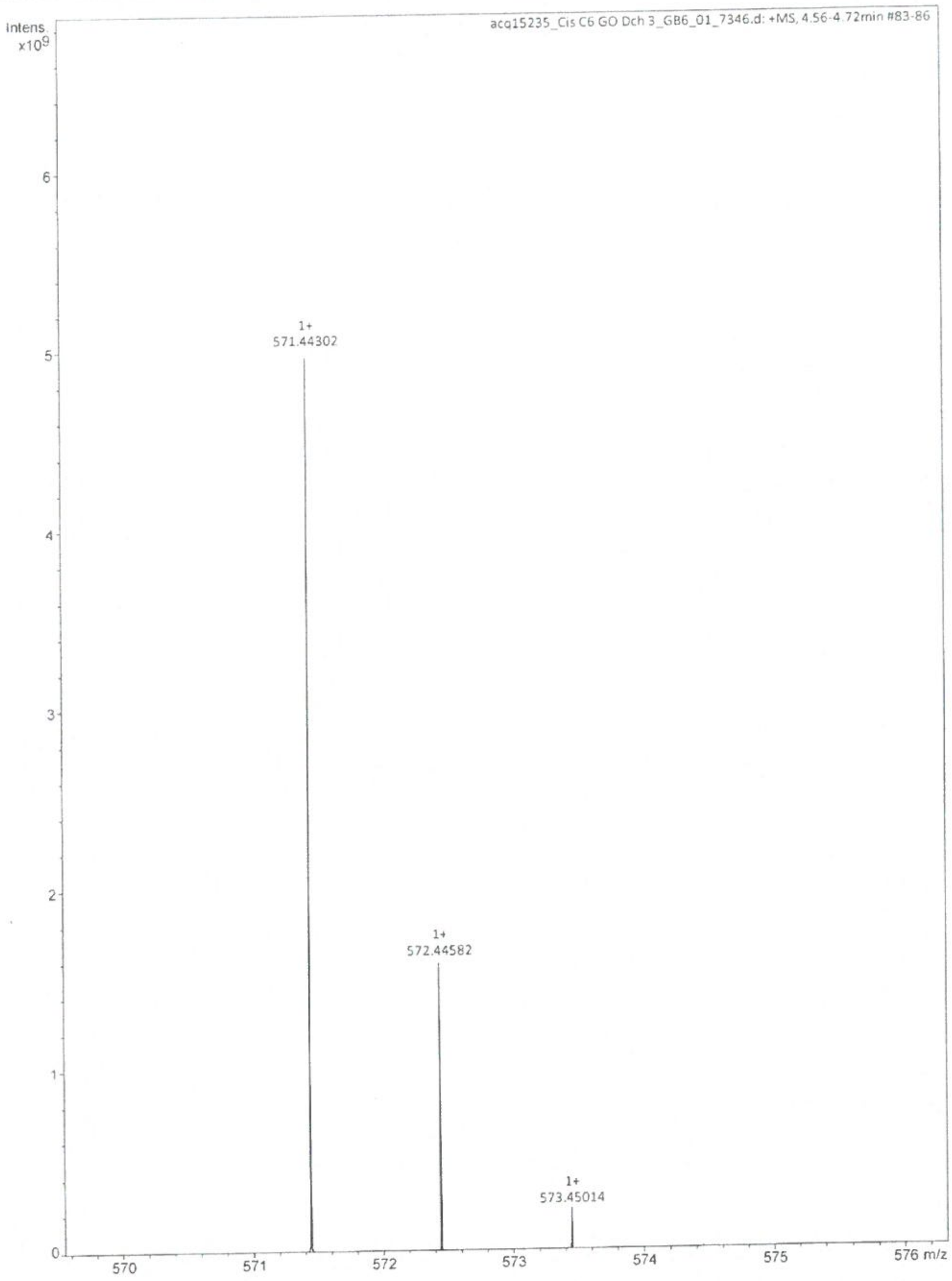

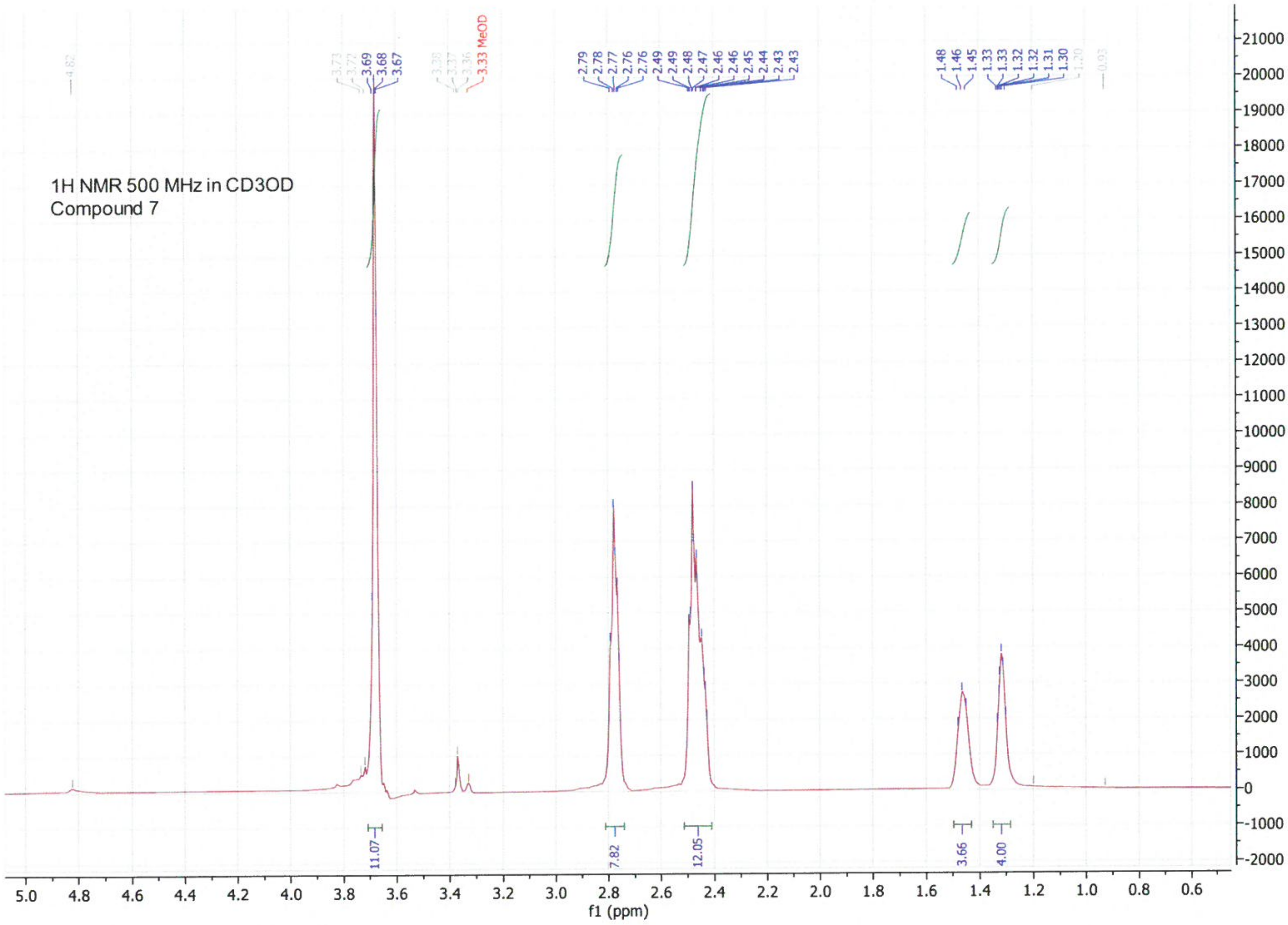
1H NMR 500 MHz in CD3OD
Compound 7
3.33 MeOD
f1 (ppm)

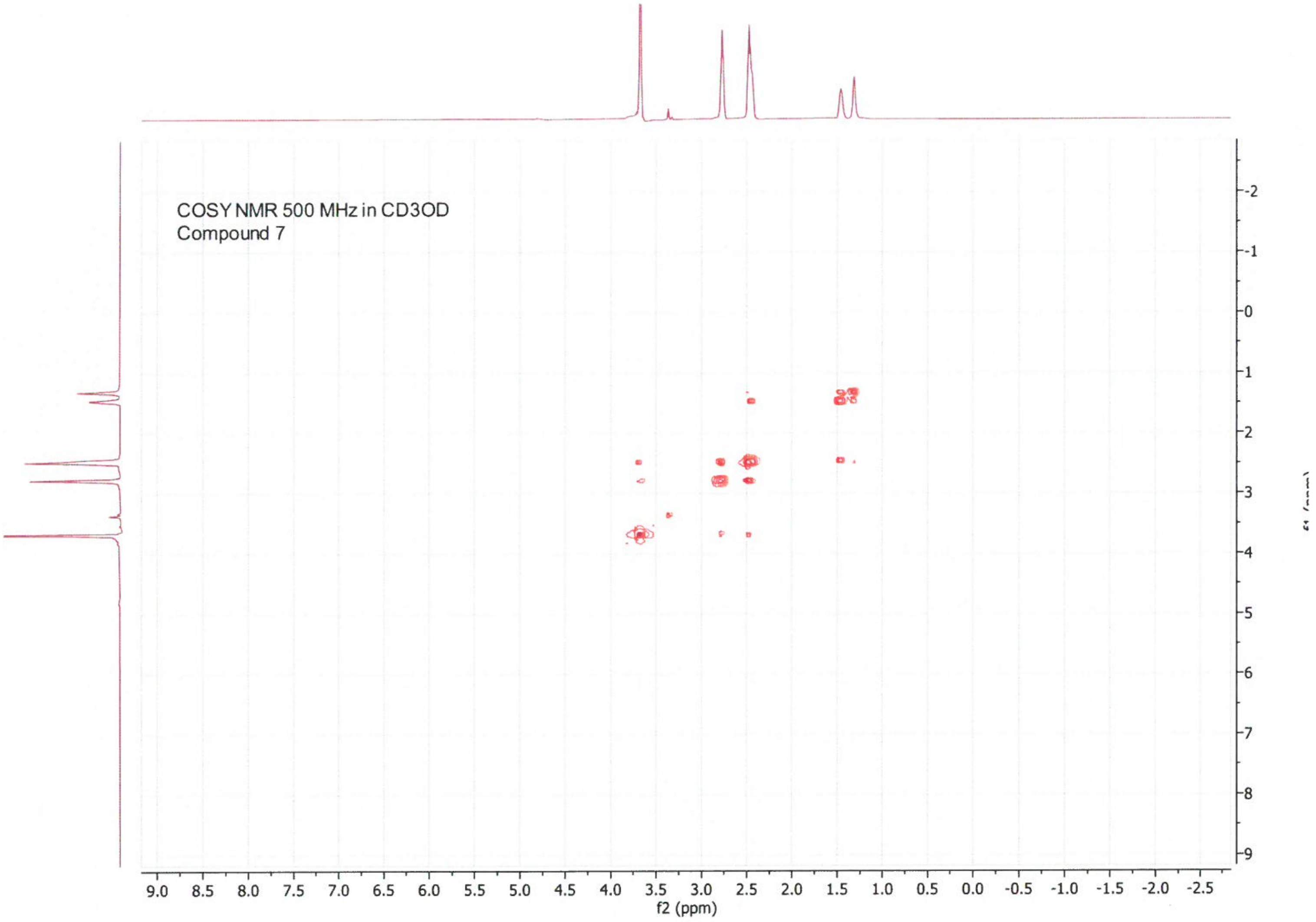
COSY NMR 500 MHz in CD3OD
Compound 7
9.0 8.5 8.0 7.5 7.0 6.5 6.0 5.5 5.0 4.5 4.0 3.5 3.0 2.5 2.0 1.5 1.0 0.5 0.0 -0.5 -1.0 -1.5 -2.0 -2.5
f2 (ppm)
-2 -1 0 1 2 3 4 5 6 7 8 9
f1 (ppm)

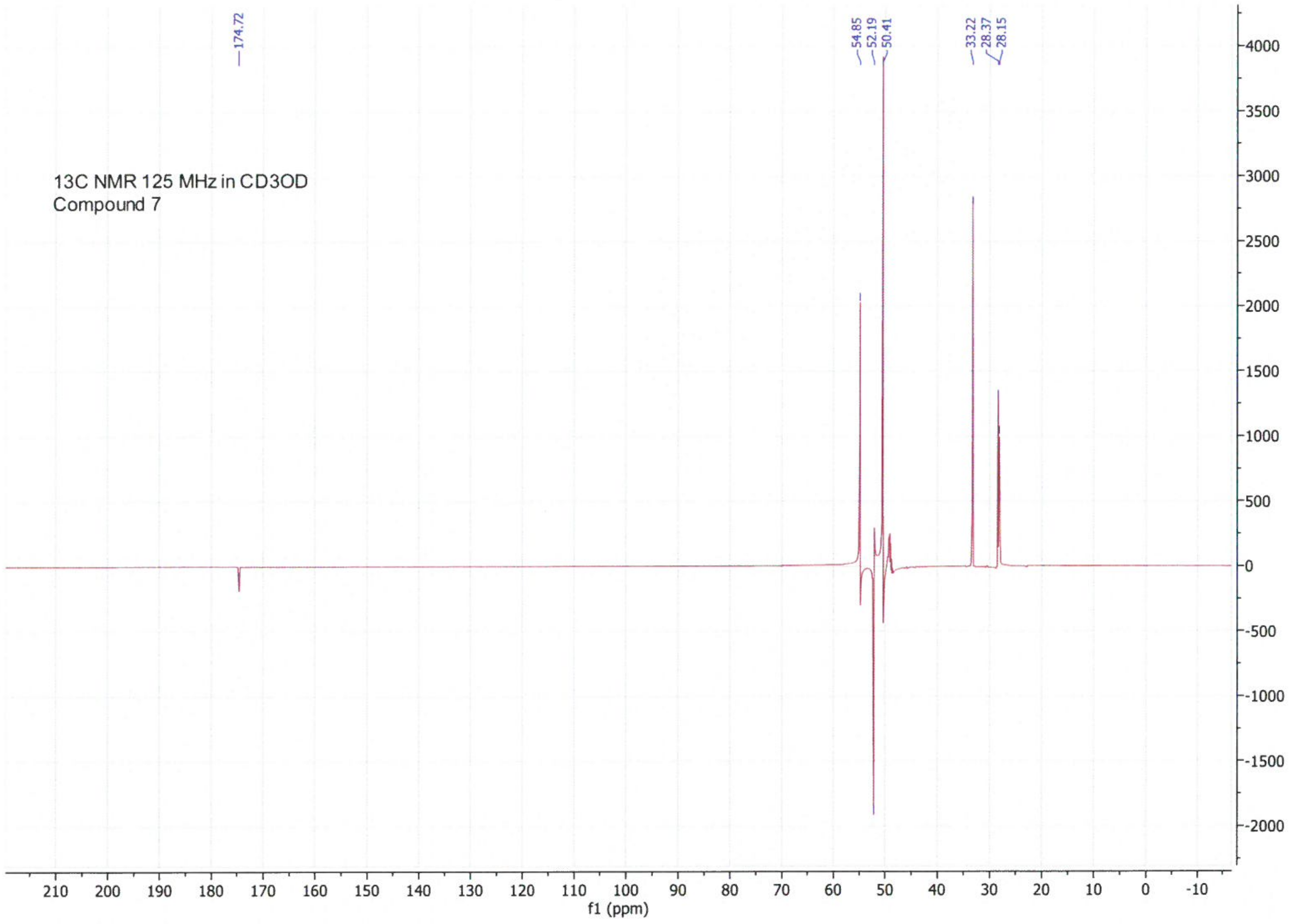

174.72
54.85
52.19
50.41
33.22
28.37
28.15
13C NMR 125 MHz in CD3OD
Compound 7
4000
3500
3000
2500
2000
1500
1000
500
0
-500
-1000
-1500
-2000
210 200 190 180 170 160 150 140 130 120 110 100 90 80 70 60 50 40 30 20 10 0 -10
f1 (ppm)

Compound 7

Window Display Report

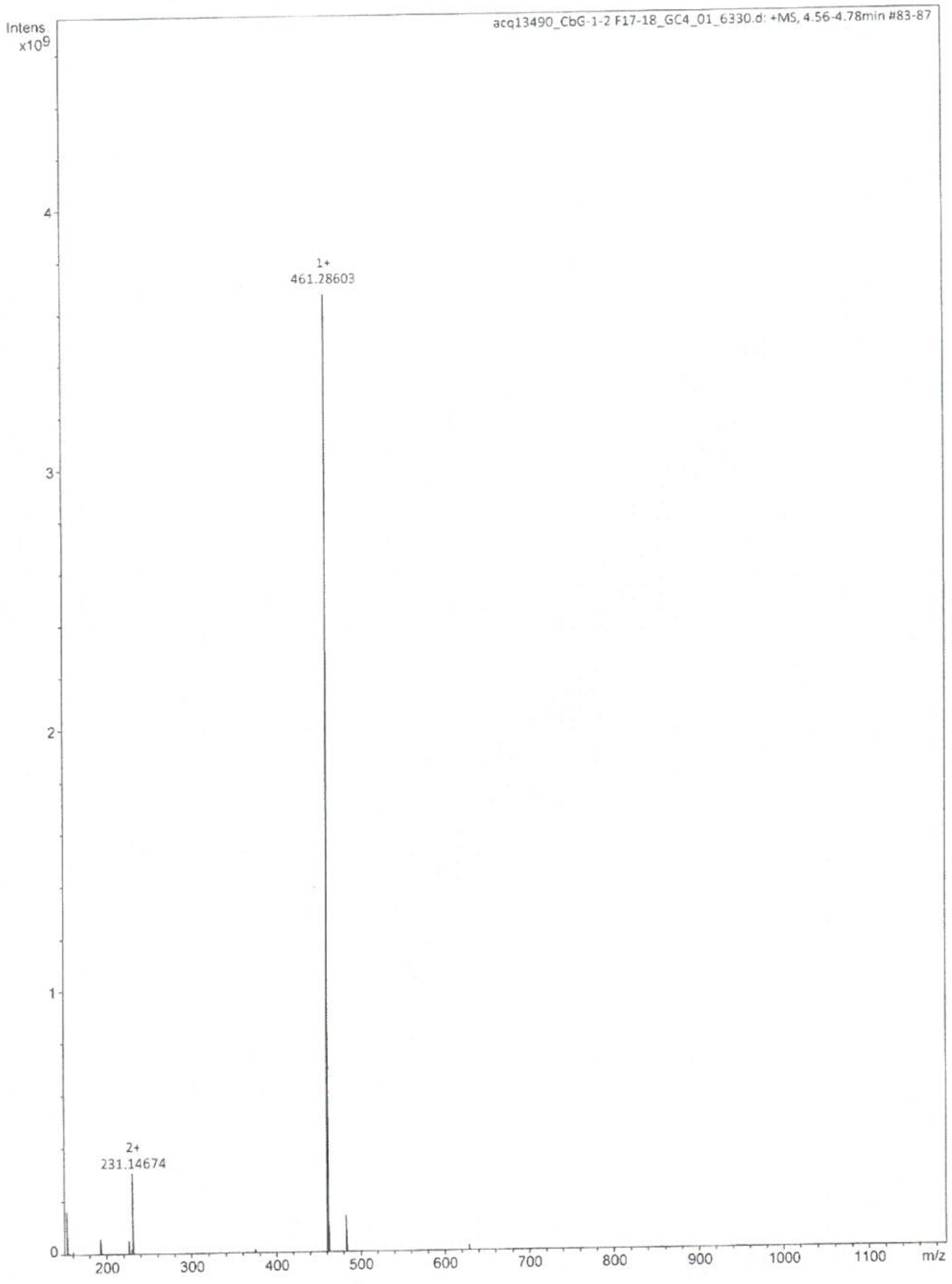

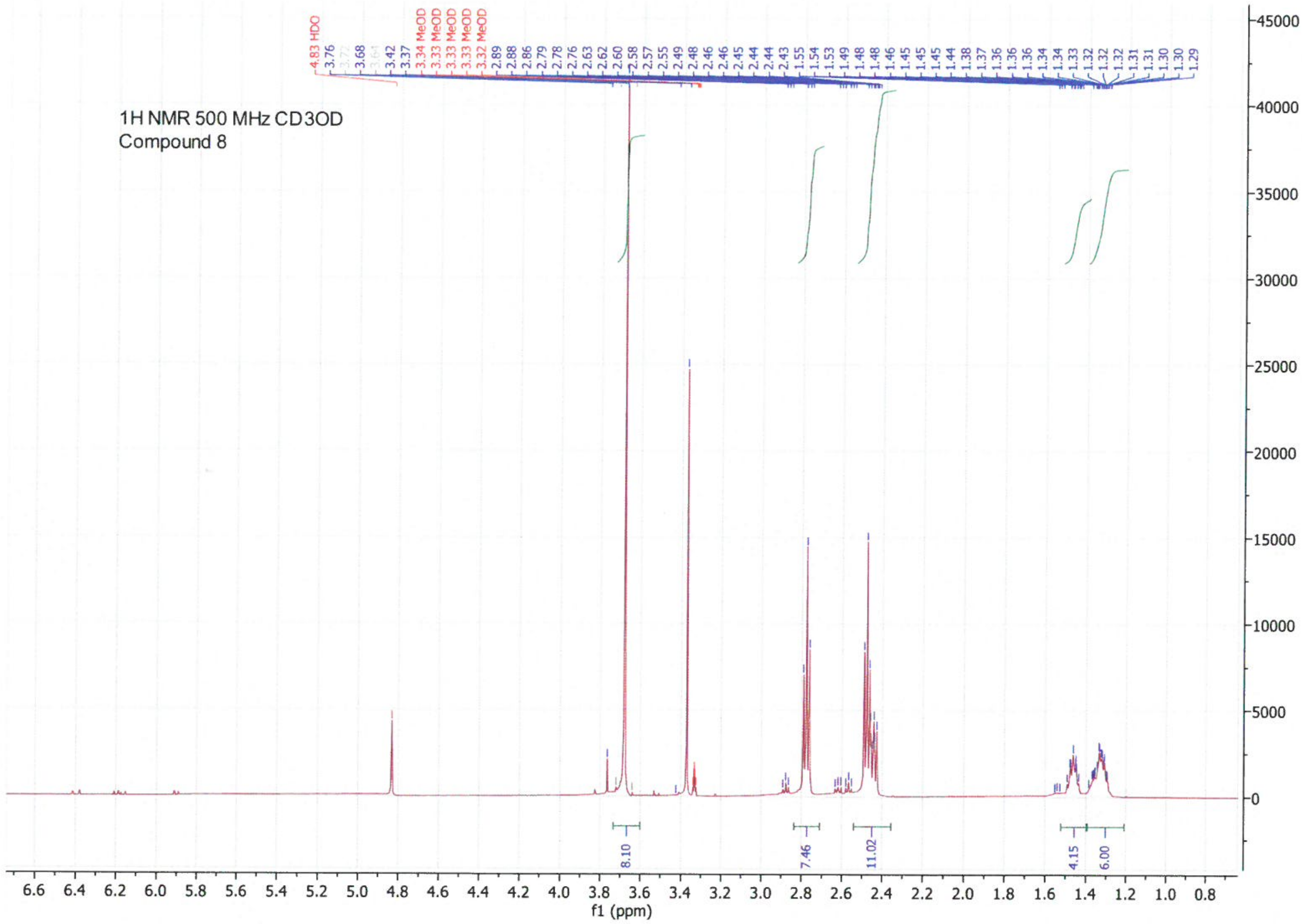
1H NMR 500 MHz CD3OD
Compound 8
f1 (ppm)

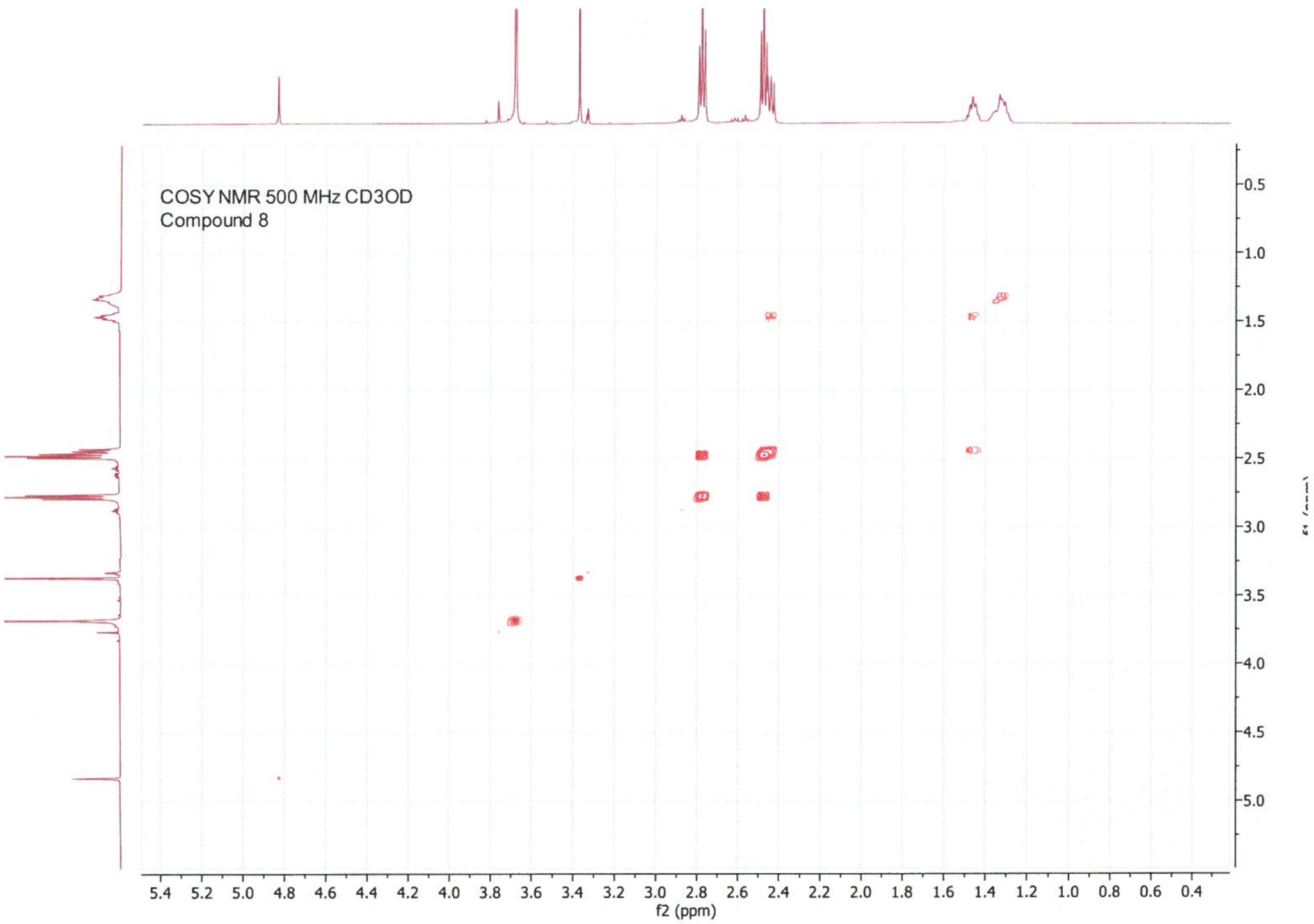

COSY NMR 500 MHz CD3OD
Compound 8
f2 (ppm)
f1 (ppm)

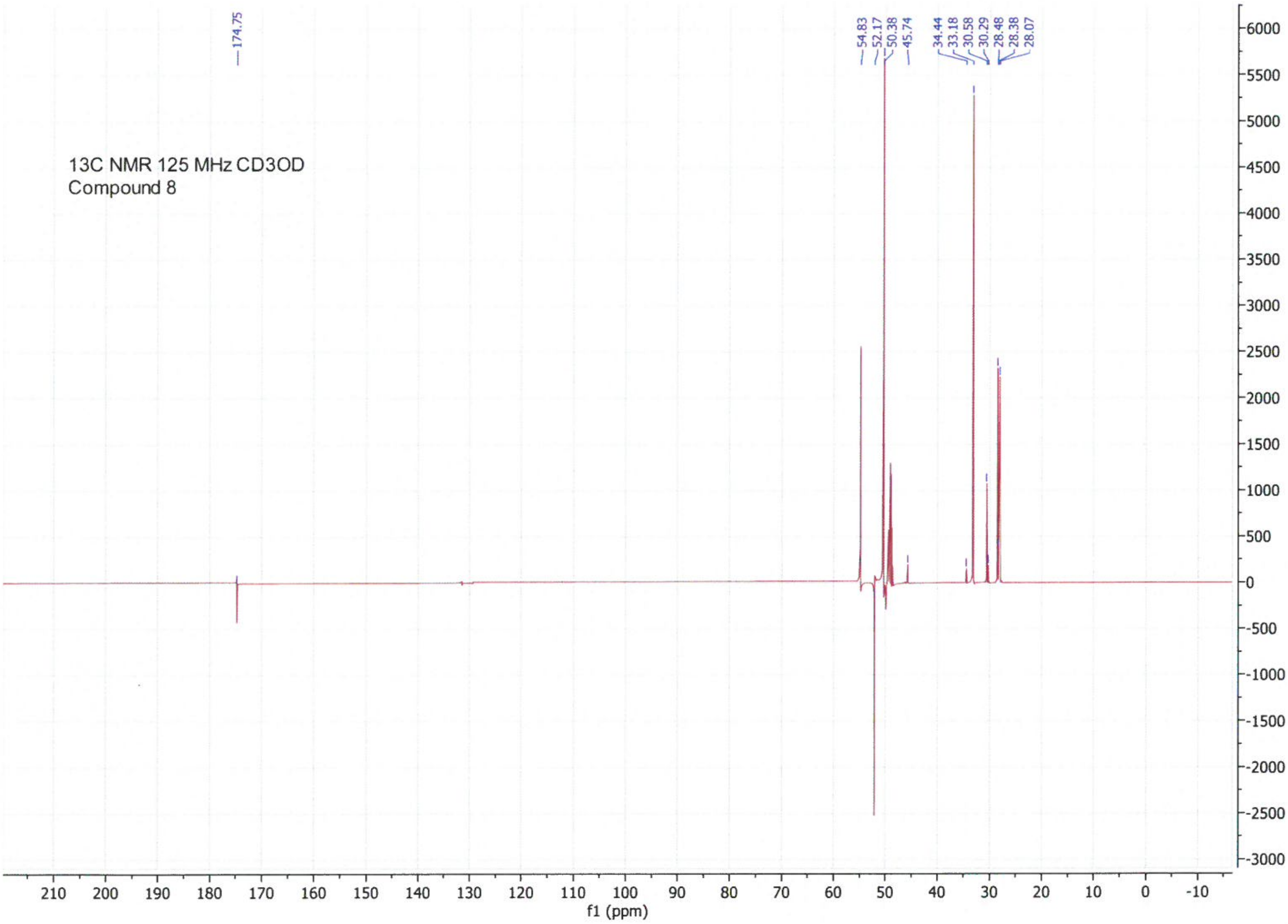

174.75
54.83
52.17
50.38
45.74
34.44
33.18
30.58
30.29
28.48
28.38
28.07
13C NMR 125 MHz CD3OD
Compound 8
f1 (ppm)

Compound 8

Window Display Report

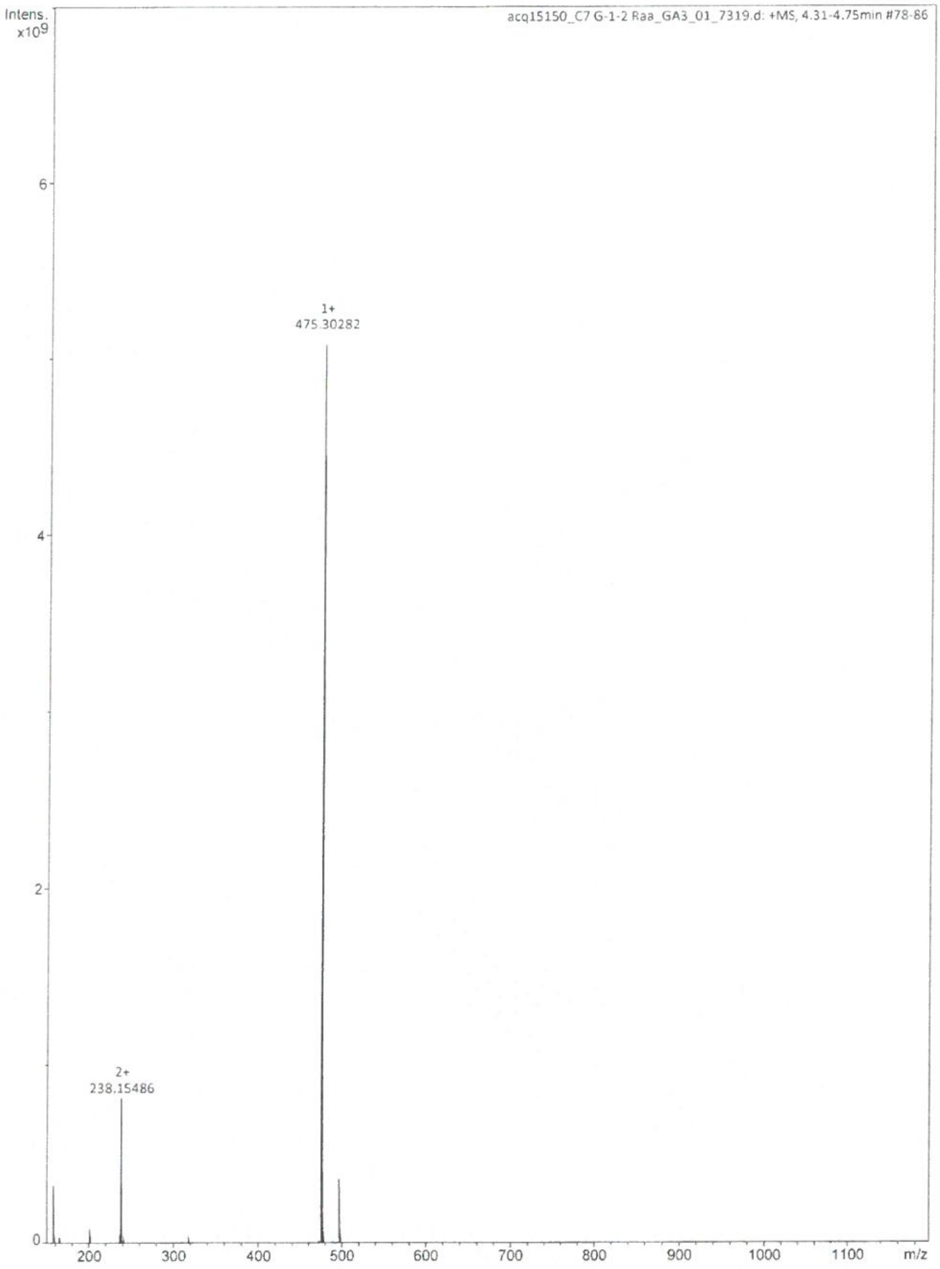

Bruker Compass DataAnalysis 5.0 printed: 6/23/2020 8:18:08 AM by: demo Page 1 of 1

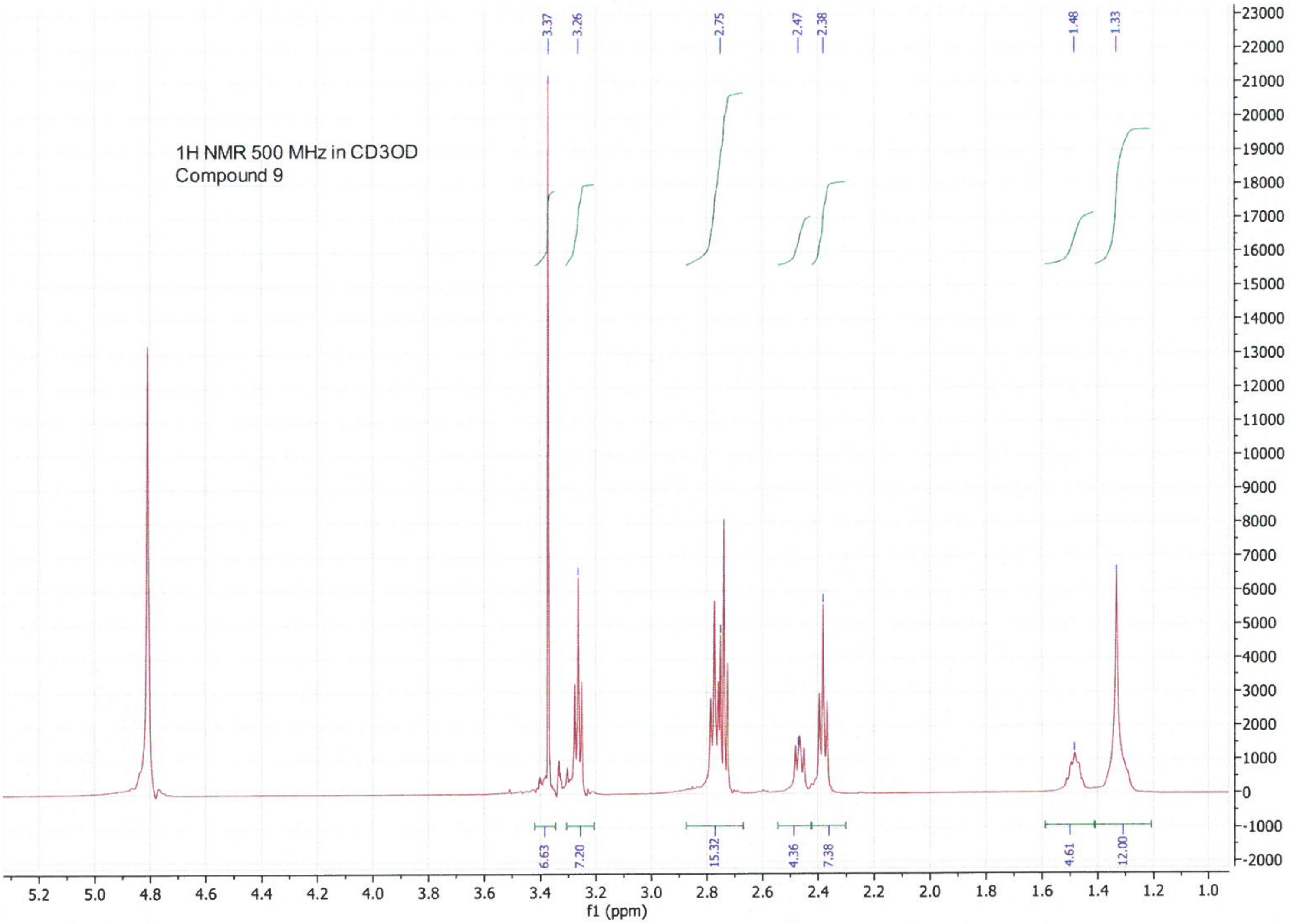
1H NMR 500 MHz in CD3OD
Compound 9
3.37
3.26
2.75
2.47
2.38
1.48
1.33
6.63
7.20
15.32
4.36
7.38
4.61
12.00
f1 (ppm)

COSY NMR 500 MHz in CD3OD
Compound 9

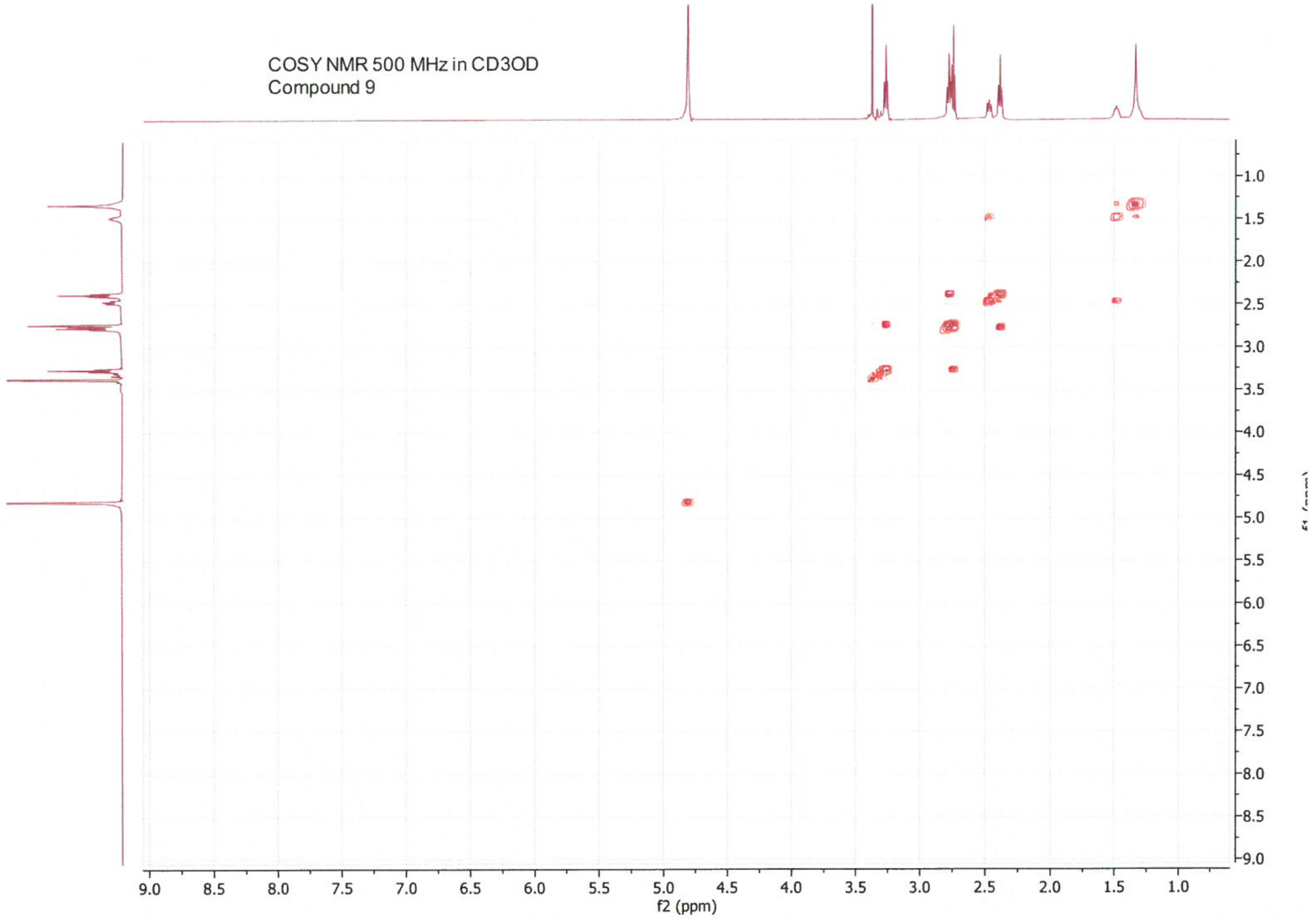

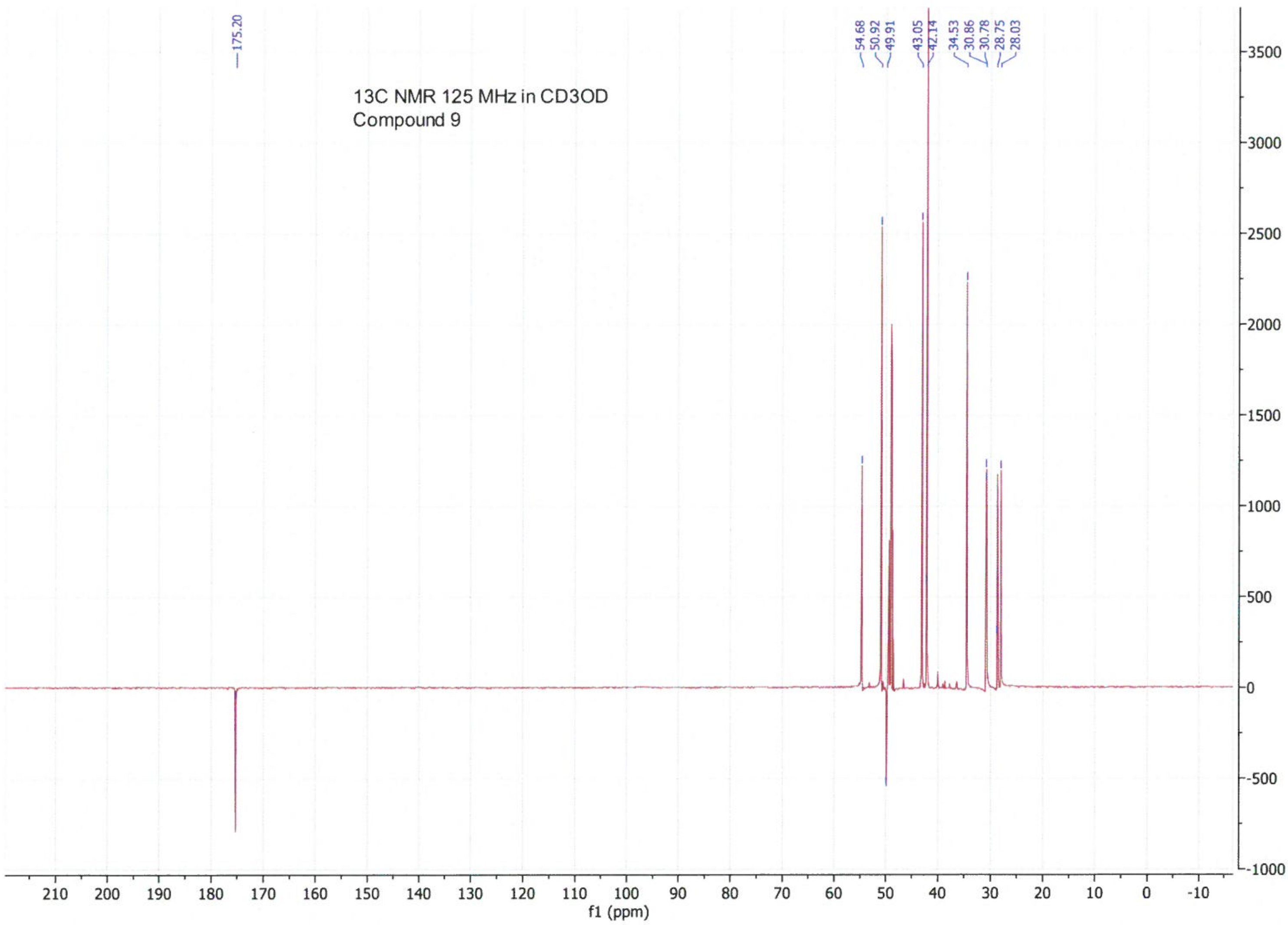

175.20
54.68
50.92
49.91
43.05
42.14
34.53
30.86
30.78
28.75
28.03
13C NMR 125 MHz in CD3OD
Compound 9
3500
3000
2500
2000
1500
1000
500
0
-500
-1000
210 200 190 180 170 160 150 140 130 120 110 100 90 80 70 60 50 40 30 20 10 0 -10
f1 (ppm)

Compound 9

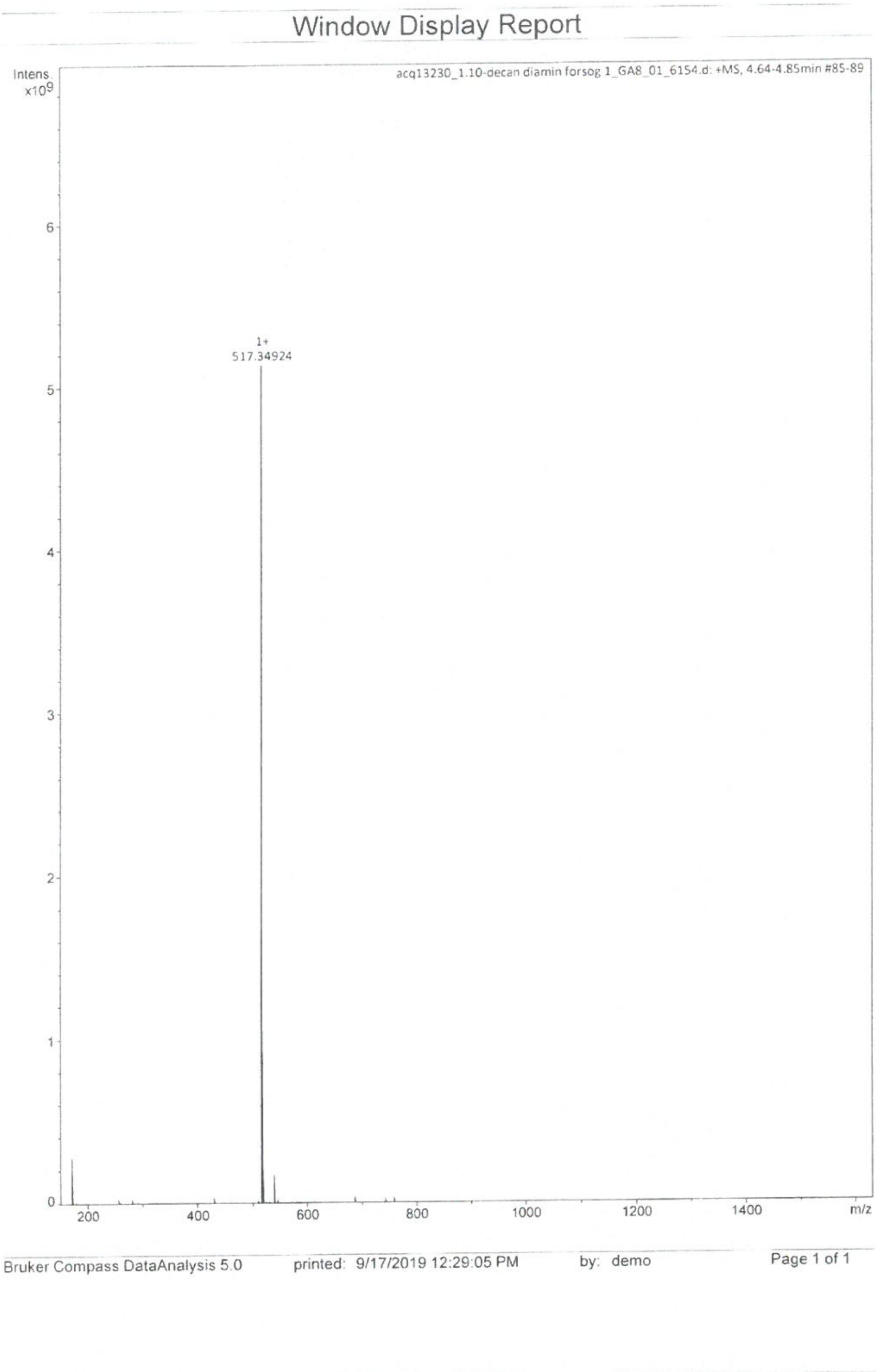
Window Display Report
Intens.
x10^9
acq13230_1.10-decan diamin forsog 1_GA8_01_6154.d: +MS, 4.64-4.85min #85-89
1+
517.34924
0
1
2
3
4
5
6
200
400
600
800
1000
1200
1400
m/z
Bruker Compass DataAnalysis 5.0
printed: 9/17/2019 12:29:05 PM
by: demo
Page 1 of 1

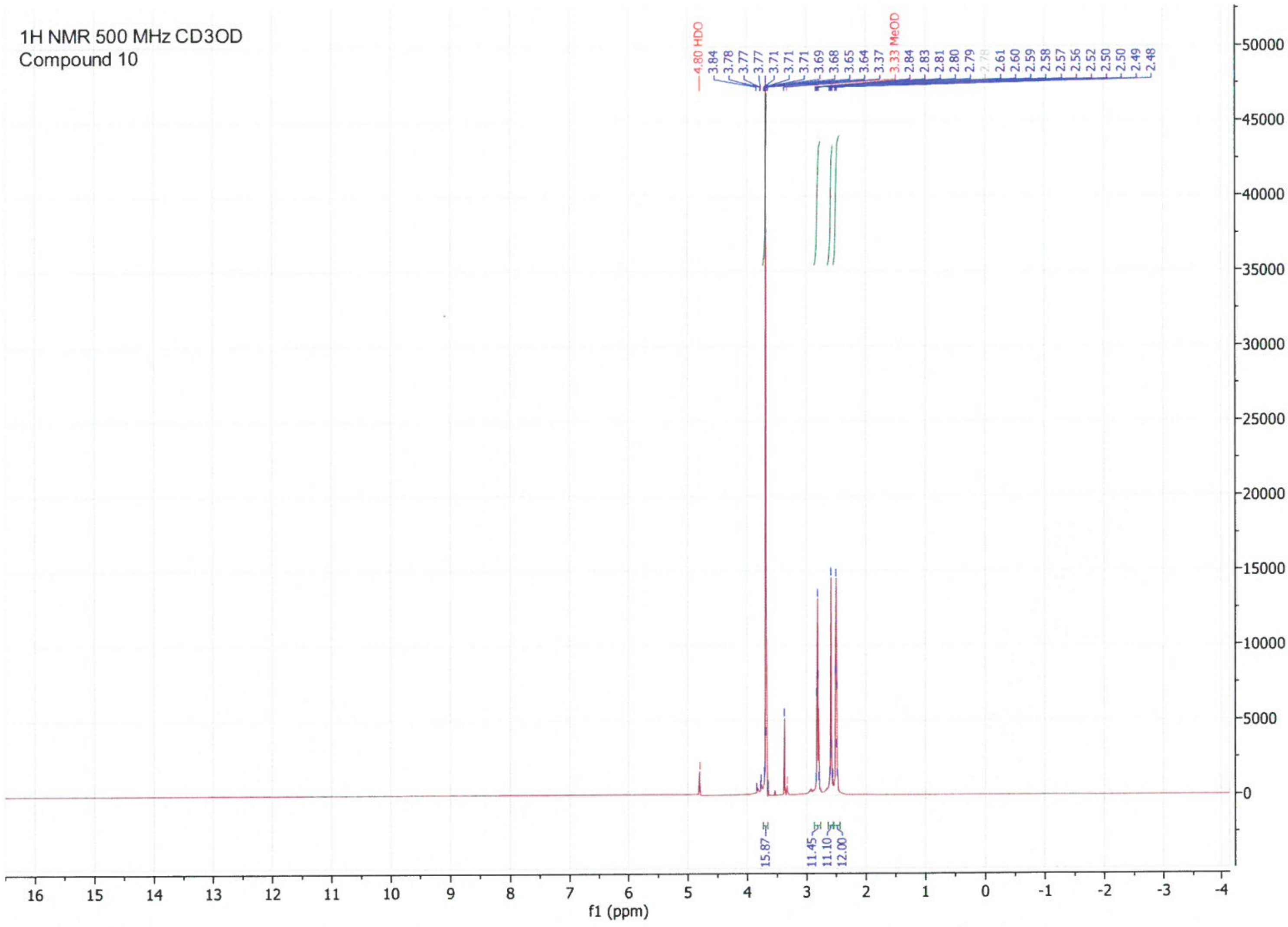
1H NMR 500 MHz CD3OD
Compound 10
4.80 HDO
3.84
3.78
3.77
3.77
3.71
3.71
3.71
3.69
3.68
3.65
3.64
3.37
3.33 MeOD
2.84
2.83
2.81
2.80
2.79
2.78
2.61
2.60
2.59
2.58
2.57
2.56
2.52
2.50
2.50
2.49
2.48
15.87
11.45
11.10
12.00
f1 (ppm)

COSY NMR 500 MHz CD3OD
Compound 10

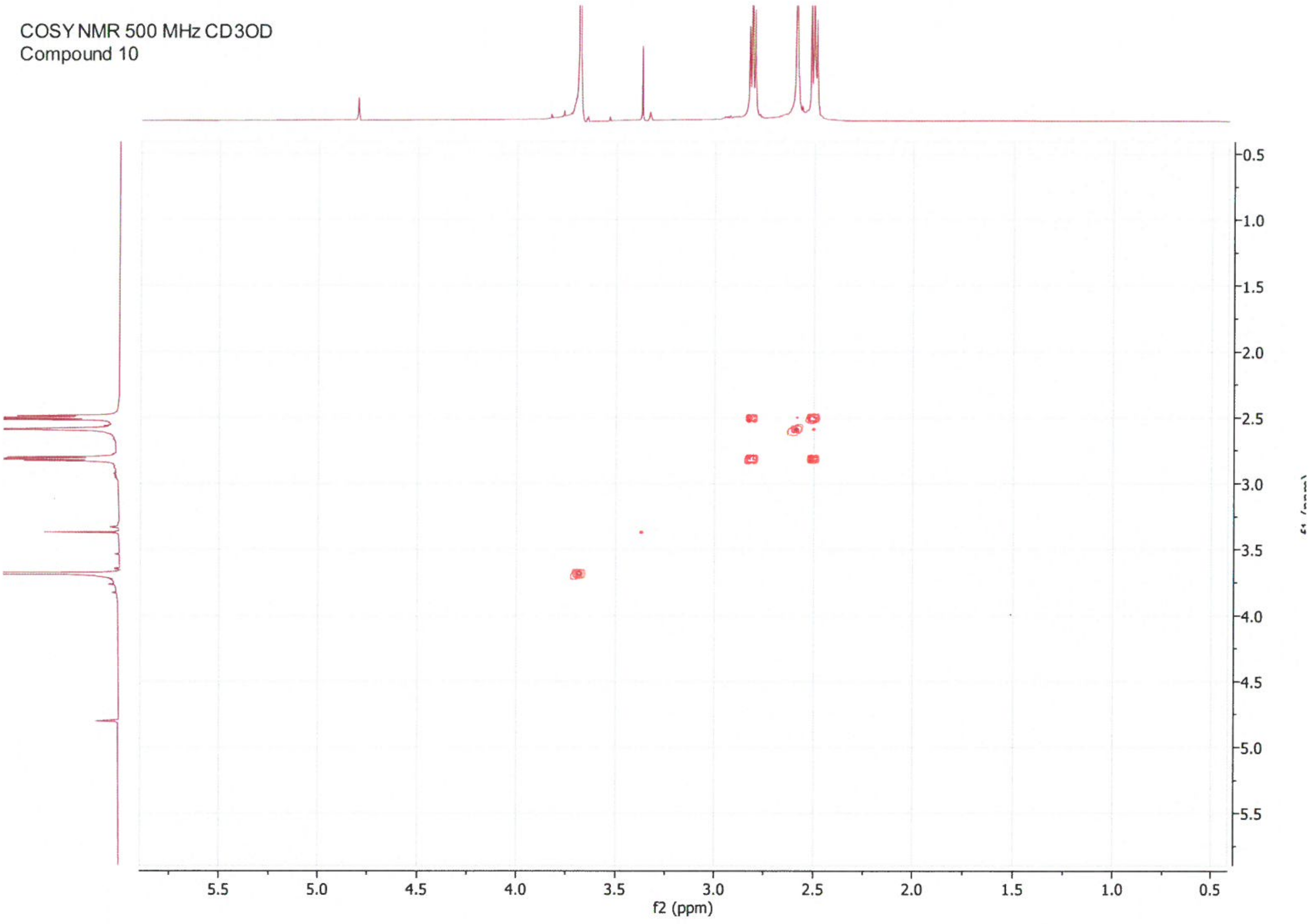

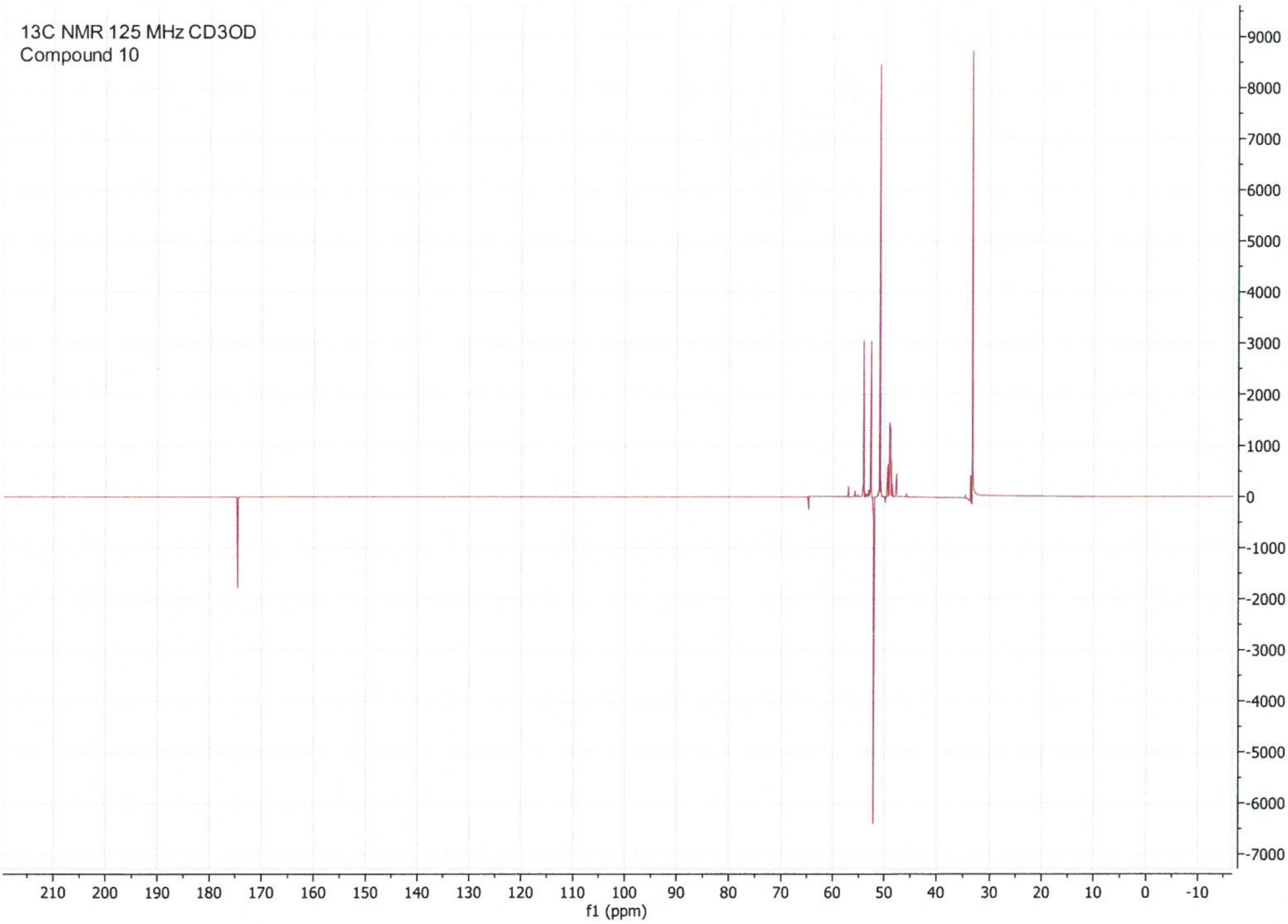

13C NMR 125 MHz CD3OD
Compound 10
9000
8000
7000
6000
5000
4000
3000
2000
1000
0
-1000
-2000
-3000
-4000
-5000
-6000
-7000
210 200 190 180 170 160 150 140 130 120 110 100 90 80 70 60 50 40 30 20 10 0 -10
f1 (ppm)

Compound 10

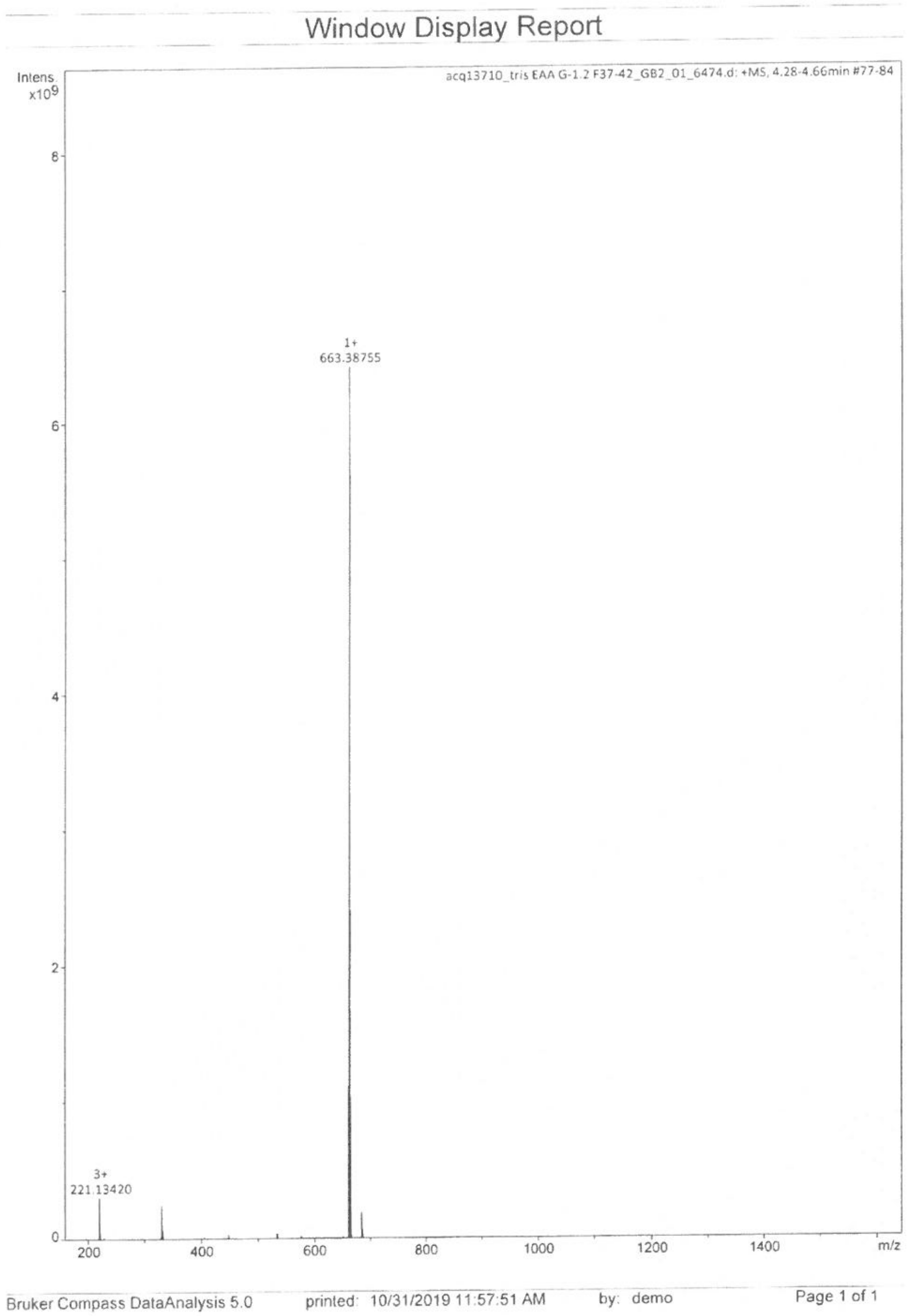
Window Display Report
Intens.
x10^9
acq13710_tris EAA G-1.2 F37-42_GB2_01_6474.d: +MS, 4.28-4.66min #77-84
1+
663.38755
3+
221.13420
m/z
Bruker Compass DataAnalysis 5.0
printed: 10/31/2019 11:57:51 AM
by: demo
Page 1 of 1

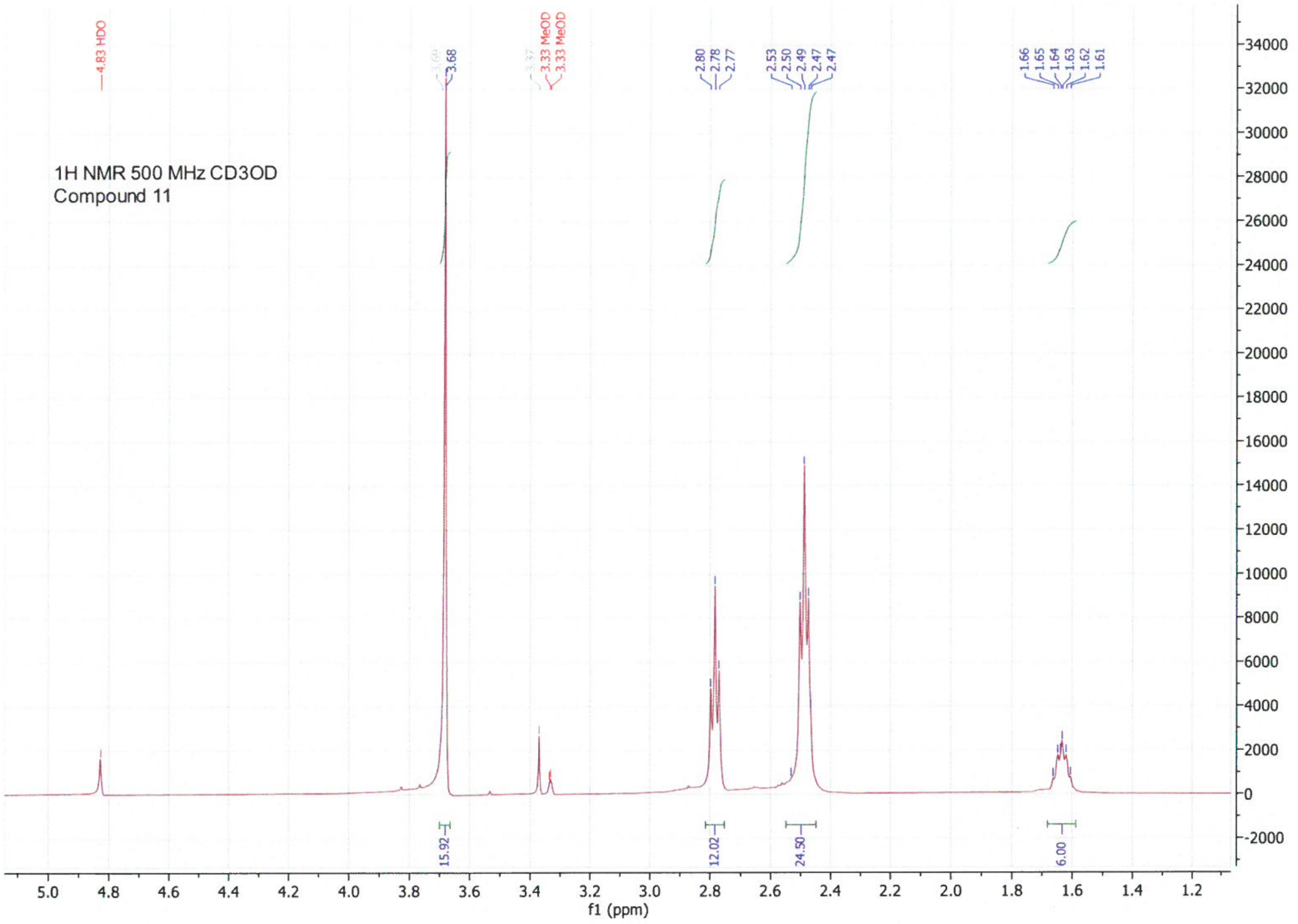

1H NMR 500 MHz CD3OD
Compound 11
4.83 HDO
3.69
3.68
3.37
3.33 MeOD
3.33 MeOD
2.80
2.78
2.77
2.53
2.50
2.49
2.47
2.47
1.66
1.65
1.64
1.63
1.62
1.61
15.92
12.02
24.50
6.00
f1 (ppm)

COSY NMR 500 MHz CD3OD
Compound 11

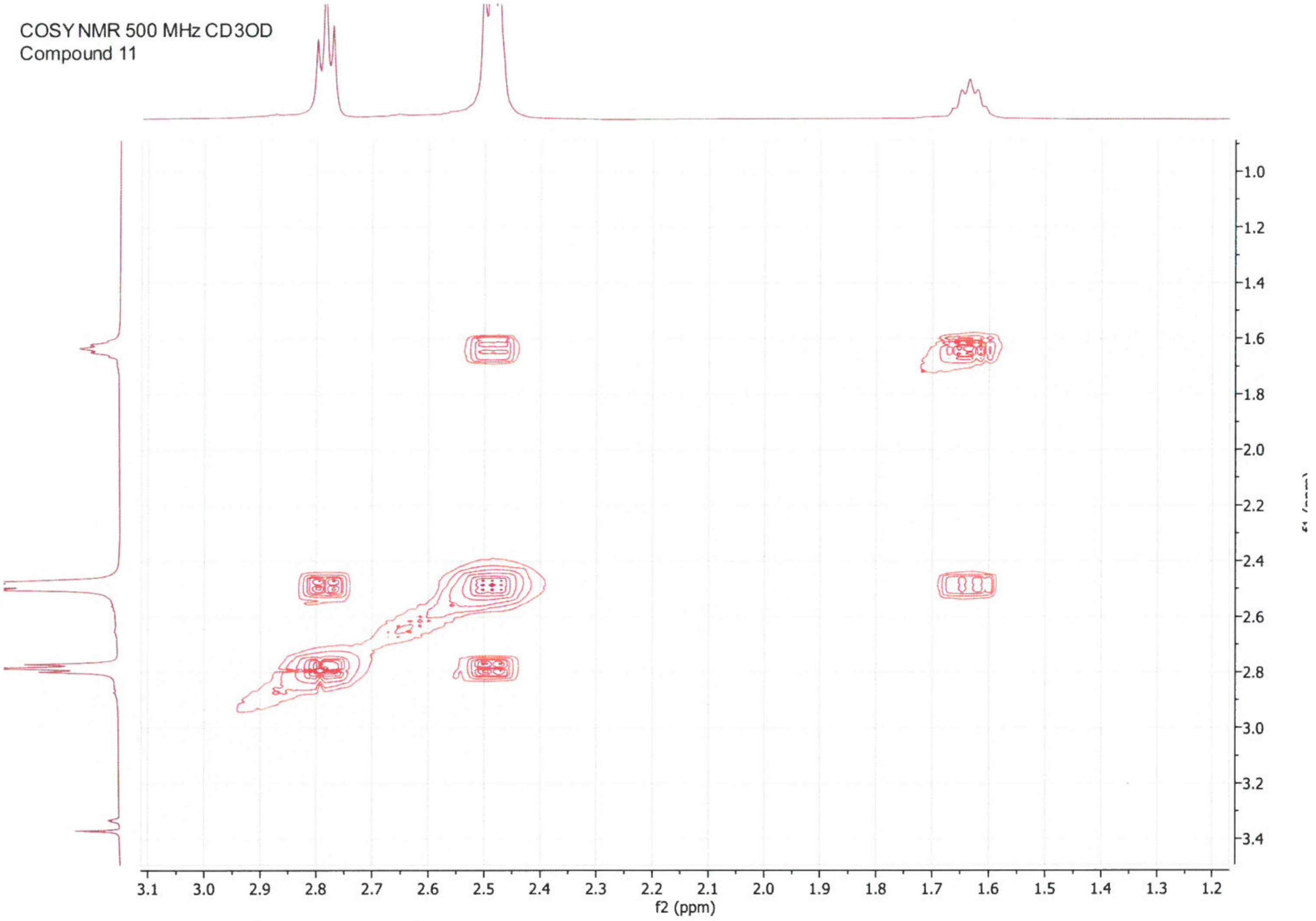

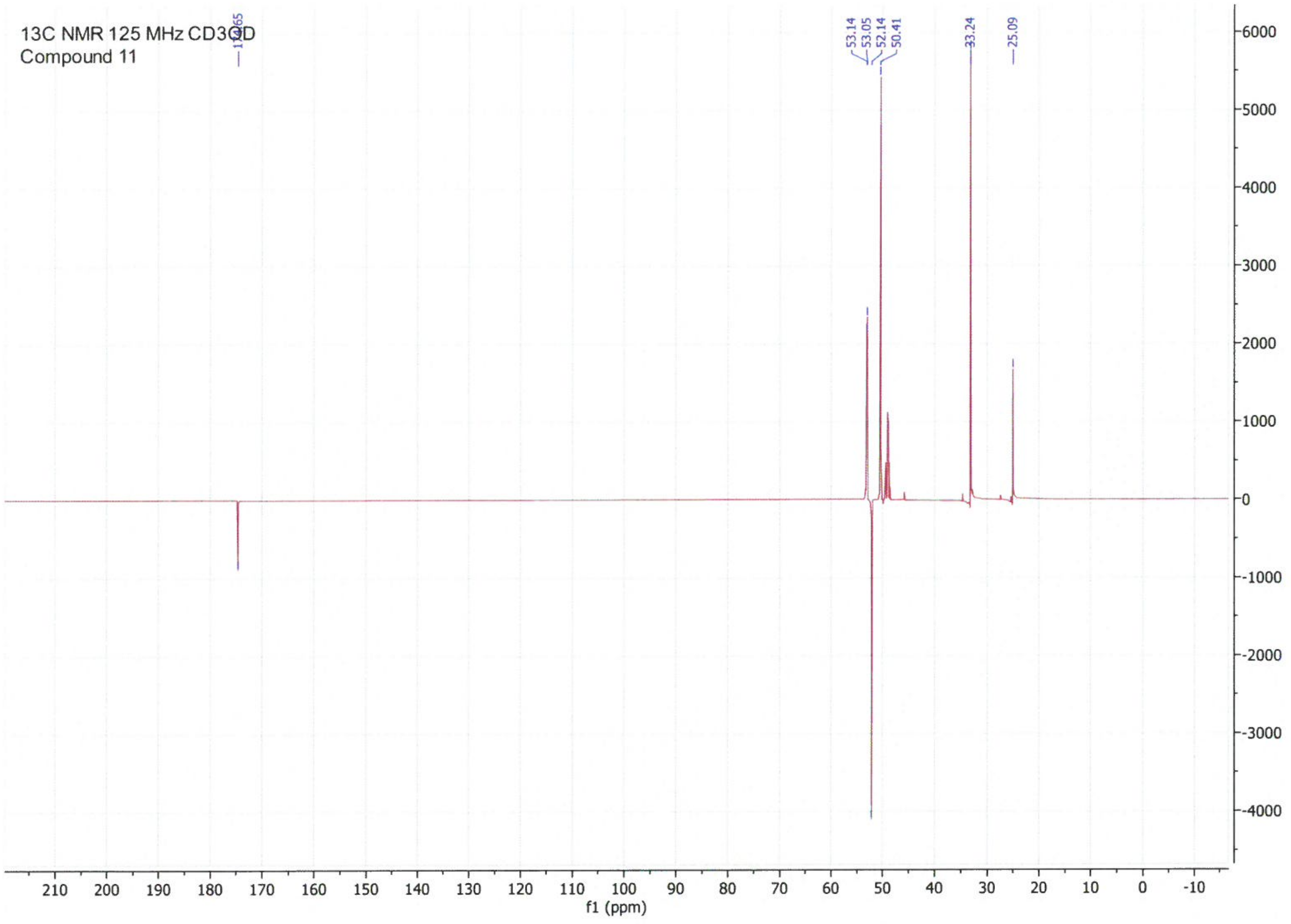
13C NMR 125 MHz CD3OD
Compound 11
174.65
53.14
53.05
52.14
50.41
33.24
25.09
6000
5000
4000
3000
2000
1000
0
-1000
-2000
-3000
-4000
210 200 190 180 170 160 150 140 130 120 110 100 90 80 70 60 50 40 30 20 10 0 -10
f1 (ppm)

Compound 11

Window Display Report

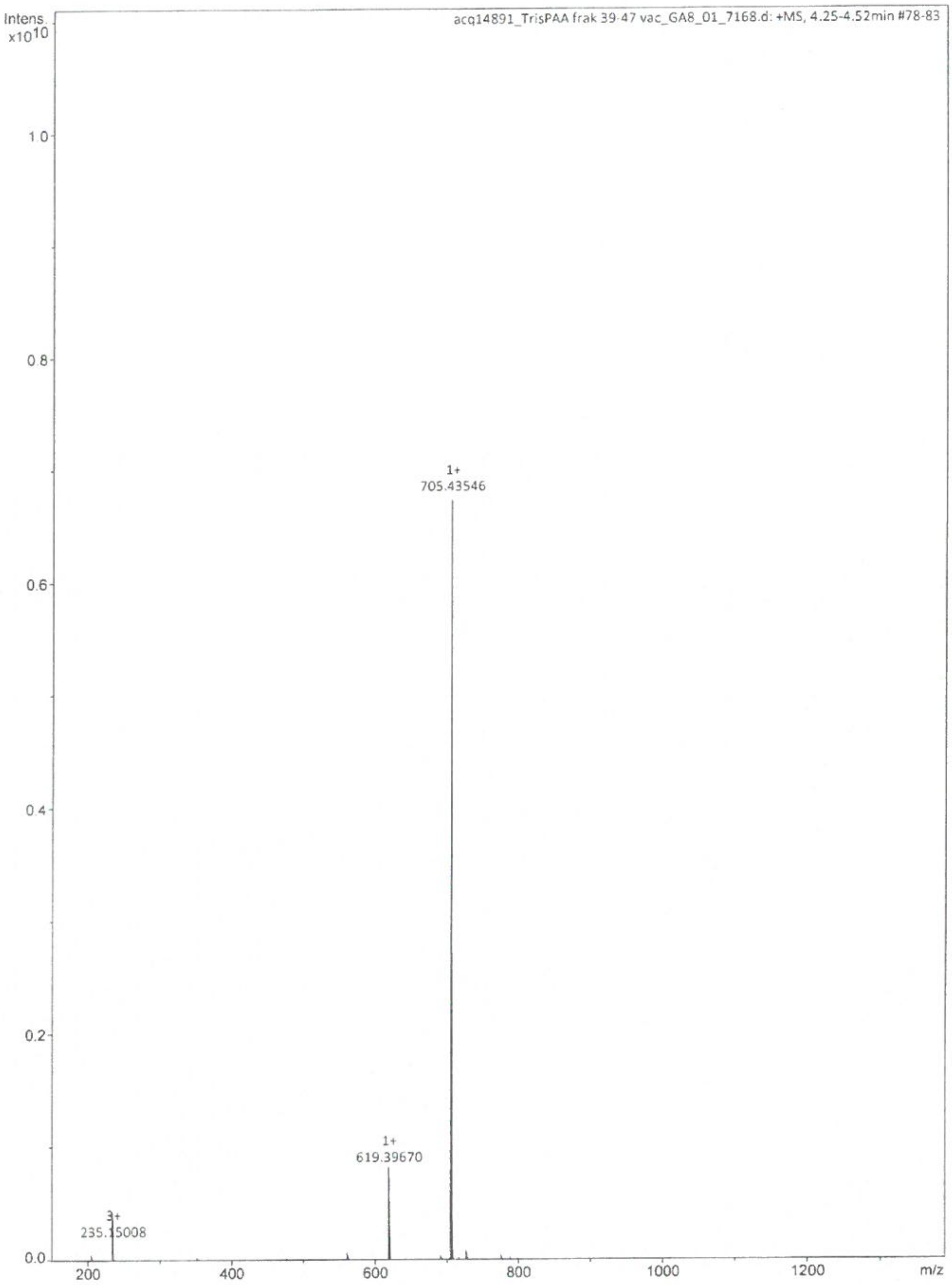

Bruker Compass DataAnalysis 5.0 printed: 5/27/2020 11:44:16 AM by: demo Page 1 of 1

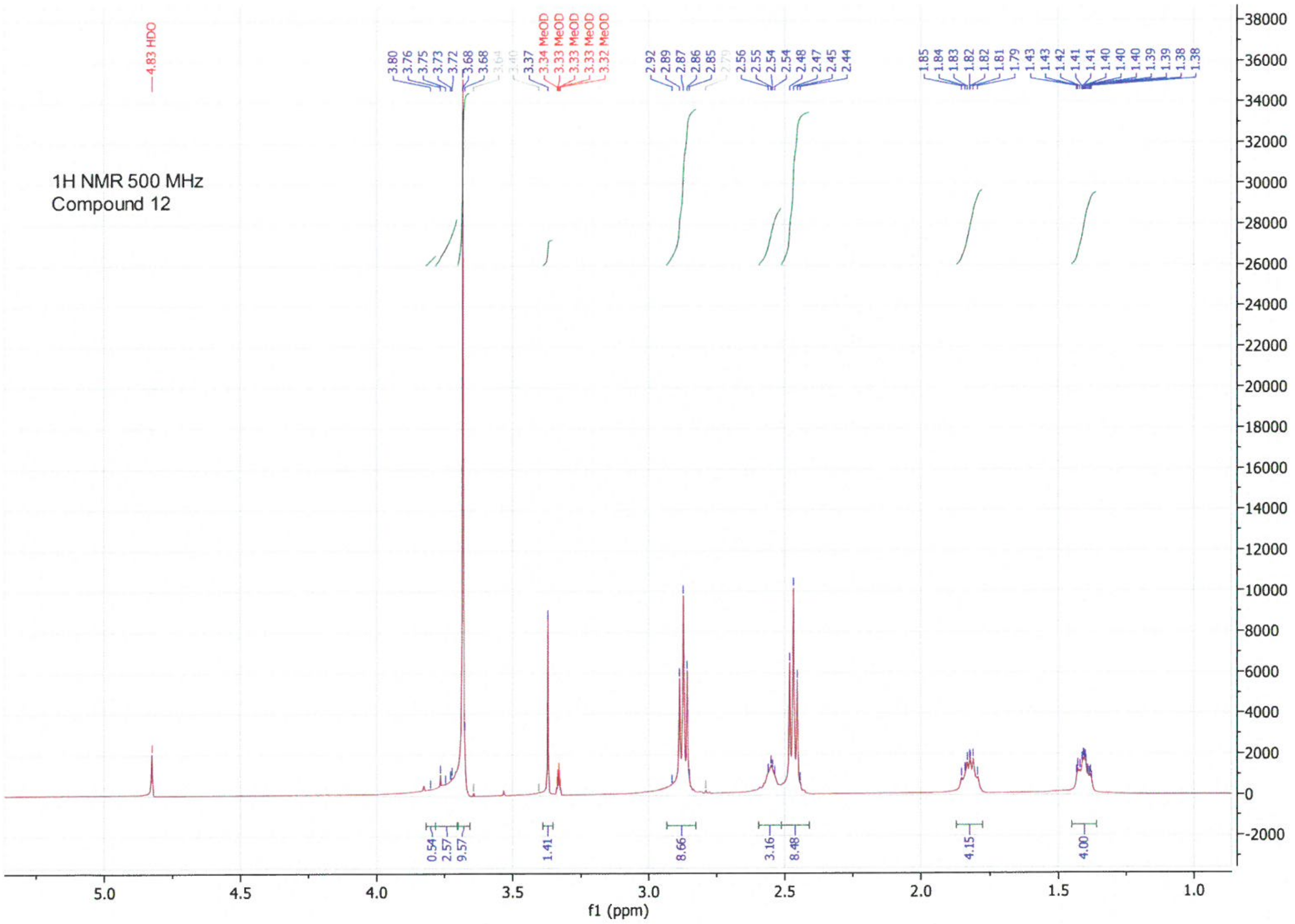

1H NMR 500 MHz
Compound 12
4.83 HDO
3.80 3.76 3.75 3.73 3.72 3.68 3.68 3.64 3.40 3.37
3.34 MeOD 3.33 MeOD 3.33 MeOD 3.33 MeOD 3.32 MeOD
2.92 2.89 2.87 2.86 2.85 2.79 2.56 2.55 2.54 2.54 2.48 2.47 2.45 2.44
1.85 1.84 1.83 1.82 1.82 1.81 1.79 1.43 1.43 1.42 1.41 1.41 1.40 1.40 1.39 1.39 1.38 1.38
0.54 2.57 9.57 1.41 8.66 3.16 8.48 4.15 4.00
f1 (ppm)

COSY NMR 500 MHz
Compound 12

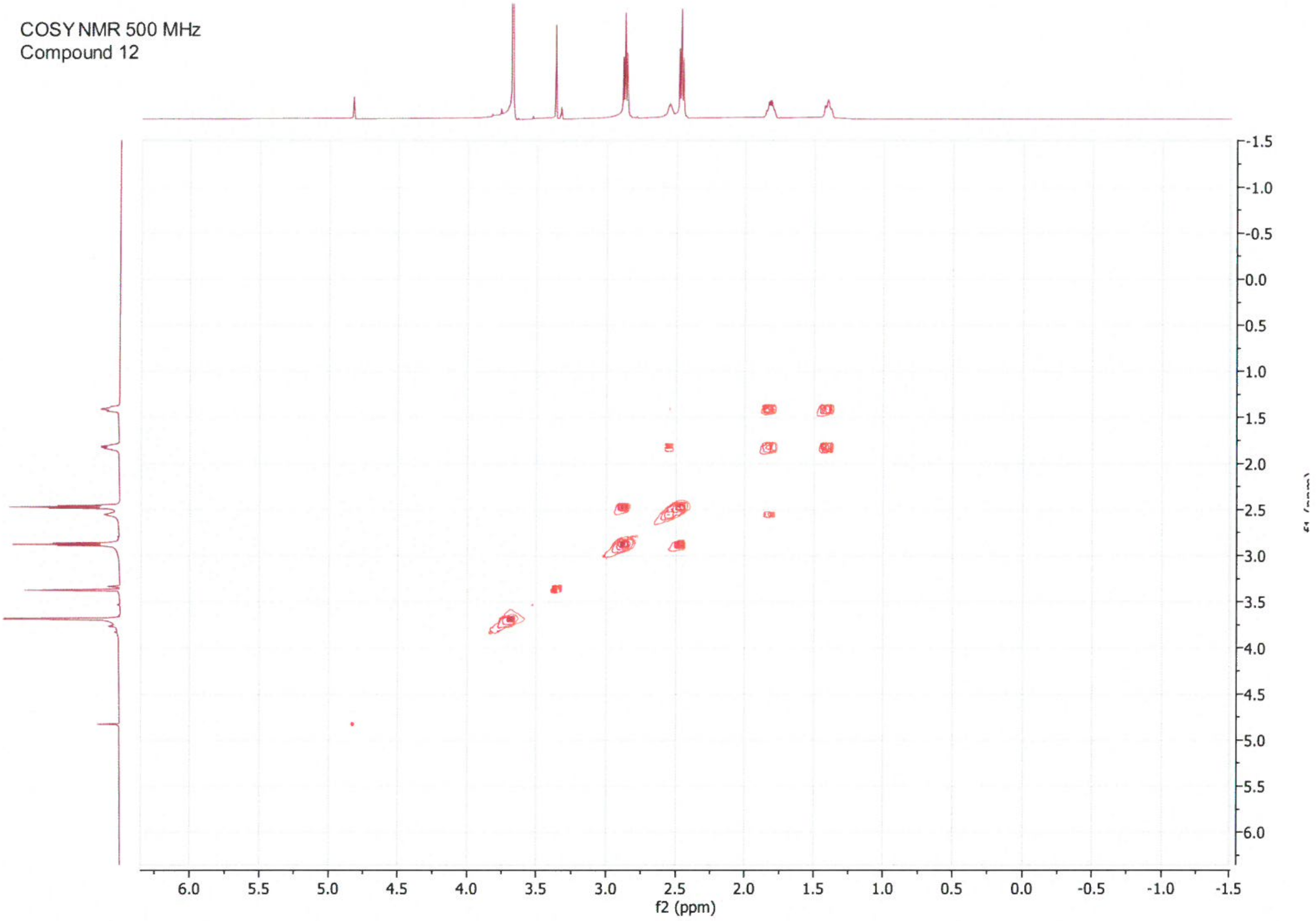

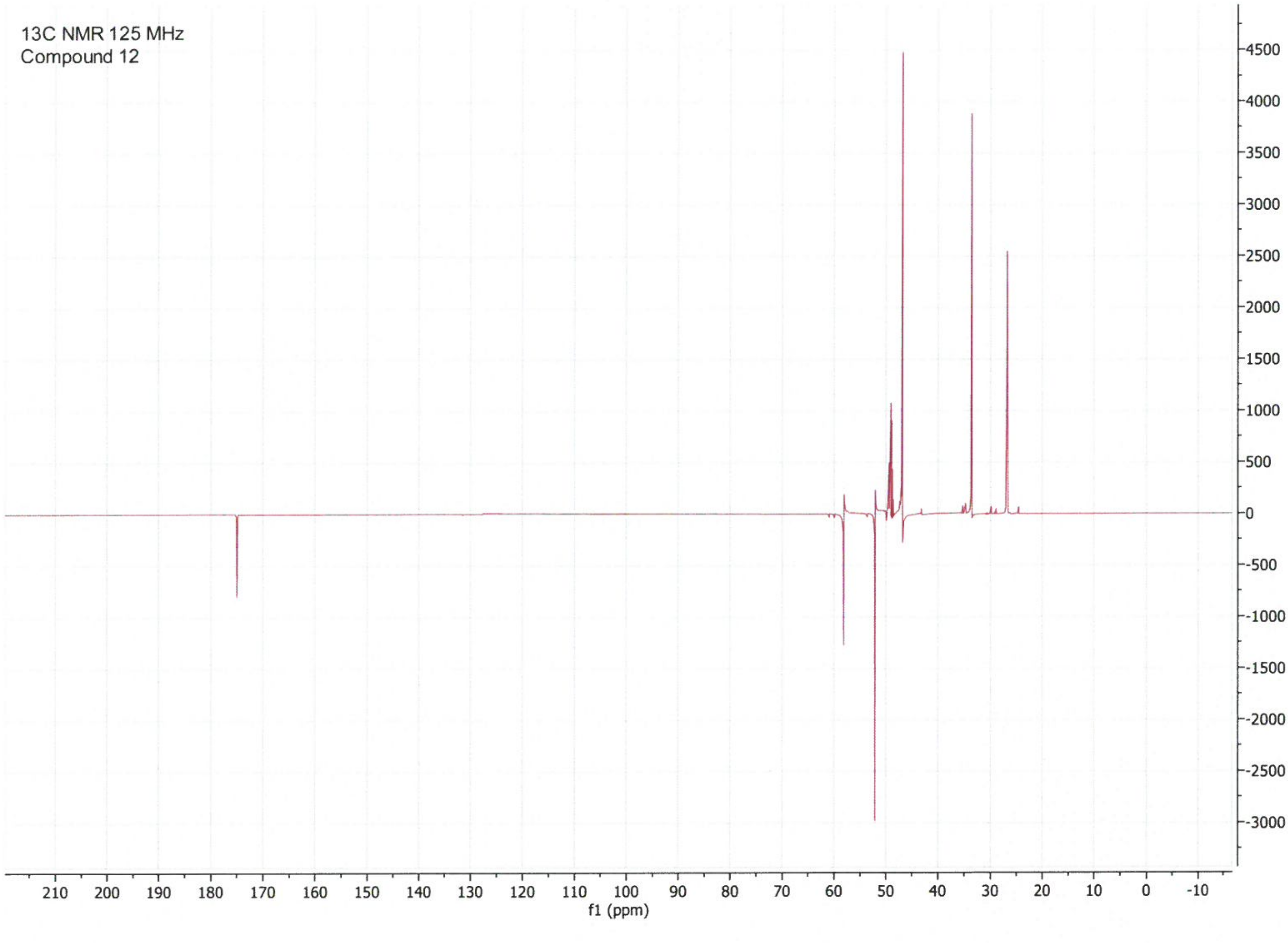
13C NMR 125 MHz
Compound 12
4500
4000
3500
3000
2500
2000
1500
1000
500
0
-500
-1000
-1500
-2000
-2500
-3000
210 200 190 180 170 160 150 140 130 120 110 100 90 80 70 60 50 40 30 20 10 0 -10
f1 (ppm)

48

Compound 12

Window Display Report

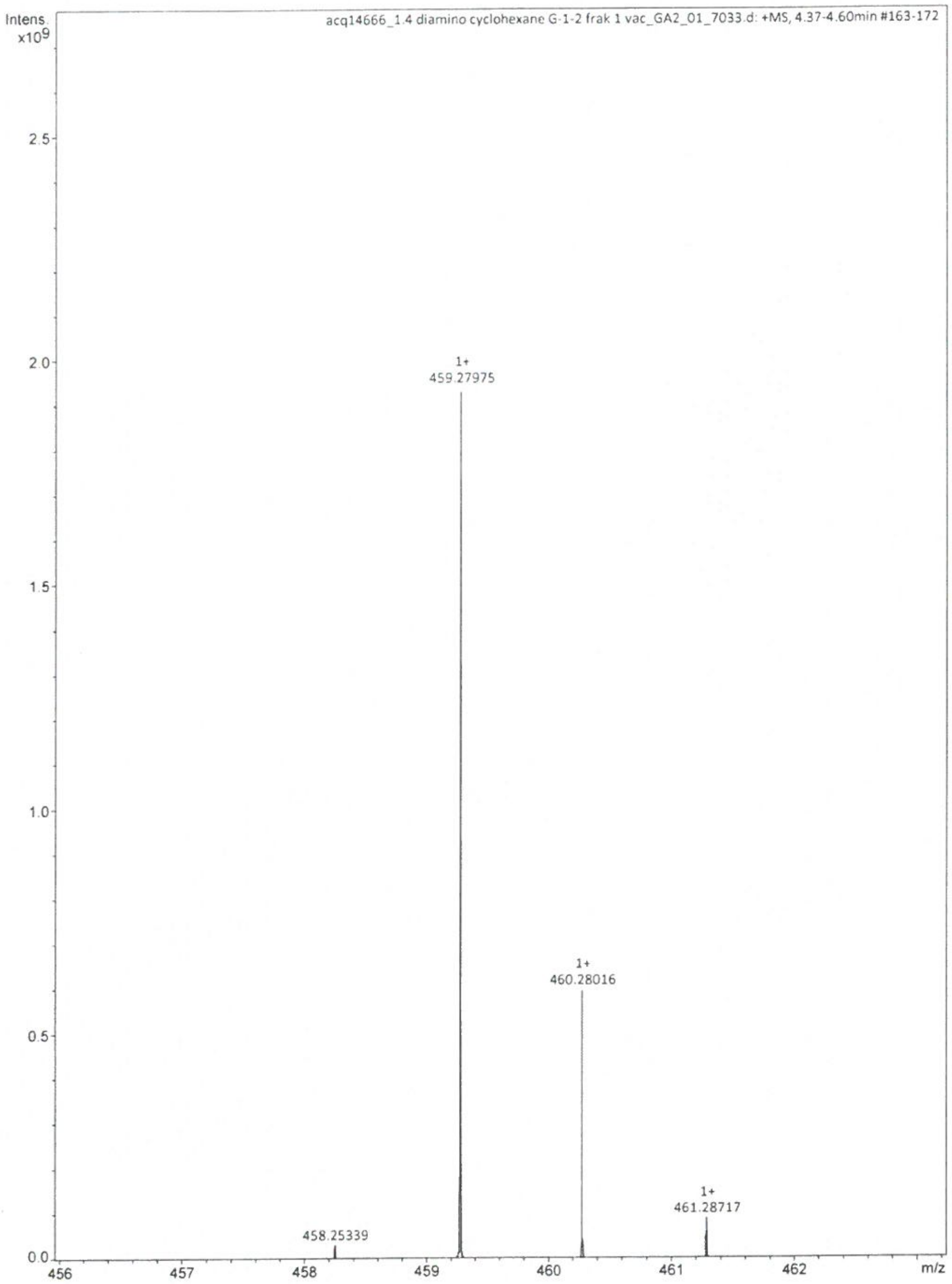

Supplement: RA-014-D4RA02020K-s001 [file RA-014-D4RA02020K-s001.pdf]
